# Targeting ferroptosis: novel therapeutic approaches and intervention strategies for kidney diseases

**DOI:** 10.3389/fimmu.2025.1700004

**Published:** 2025-12-05

**Authors:** Yanfang Luo, Muyang Long, Xueqin Wu, Liuting Zeng

**Affiliations:** 1 Department of Nephrology, The Central Hospital of Shaoyang, Shaoyang, Hunan, China; 2 Department of Rheumatology and Clinical Immunology, Peking Union Medical College Hospital, Chinese Academy of Medical Sciences and Peking Union Medical College, National Clinical Research Center for Dermatologic and Immunologic Diseases (NCRC-DID), Key Laboratory of Rheumatology and Clinical Immunology, Ministry of Education, Beijing, China

**Keywords:** ferropotosis, AKI, CKD, renal tumor, therapeutic translation, oxidative stress, iron metabolism

## Abstract

Chronic kidney disease (CKD), characterized by structural, functional, and metabolic derangements, remains a leading cause of end-stage renal disease (ESRD) with profound global health burdens. The kidney’s high oxygen demand for blood filtration renders it exquisitely sensitive to redox imbalance—an aberration common to both CKD and acute kidney injury (AKI) that, when coupled with iron dysregulation, unleashes ferroptosis: a non-apoptotic, iron-dependent form of regulated cell death driven by iron accumulation, lipid peroxidation, and antioxidant defense impairment (e.g., GPX4/SLC7A11 dysfunction), cascades to which the redox-sensitive kidney is uniquely predisposed. While ferroptosis has been linked to AKI, diabetic nephropathy (DN), and renal fibrosis, existing reviews largely suffer from two limitations: they either focus on single kidney disease entities (e.g., only AKI or DN) or reiterate generic ferroptosis mechanisms, lacking a unified pathophysiological framework that bridges acute insults, chronic fibrosis, and even renal carcinogenesis. Addressing this gap, this review offers three integrated contributions: first, it positions ferroptosis as a convergent metabolic executioner across a broader spectrum of kidney diseases—encompassing AKI, DN, renal interstitial fibrosis, systemic lupus erythematosus (SLE) nephritis, autosomal dominant polycystic kidney disease (ADPKD), renal cell carcinoma (RCC), and contrast-induced nephropathy (CIN)—while emphasizing cell type-specific vulnerabilities: tubular epithelial cells (susceptible via mitochondrial dysfunction), podocytes (via iron overload), and immune cells (e.g., neutrophils/macrophages in SLE nephritis) exhibit context-dependent ferroptosis regulation, governed by cell type-specific modulators [e.g., Nrf2 in tubules, heme oxygenase-1 (HO-1) in macrophages, and sirtuins in podocytes]. Second, it reconciles seemingly disparate findings through a redox-metabolic lens—e.g., dual roles of HO-1 (protective via heme degradation *vs*. pro-ferroptotic via iron release) or iron overload (driving injury in AKI *vs*. targeted therapy in RCC)—by clarifying disease-specific regulatory mechanisms: PKD1 mutation-driven mitochondrial defects in ADPKD, DPP9-Nrf2-mediated sorafenib resistance in RCC, and PPARα–FABP1 axis dysregulation in IgA nephropathy, alongside shared core pathways (e.g., GPX4/SLC7A11 as central checkpoints). Third, it integrates translational insights rarely synthesized in prior work: mapping natural compounds (icariin II and artesunate), repurposed drugs (sorafenib and melatonin), and novel modulators to disease stages (e.g., Lip-1 for fibrosis and salinomycin for RCC stem cells); highlighting strategies to reverse ferroptosis-related drug resistance (targeting DPP9 in RCC); and identifying ferroptosis-related genes (ACSL4 and PDIA4) as prognostic biomarkers. Accumulating clinical and experimental evidence confirms ferroptosis as a pivotal driver of kidney disease onset and progression. This review not only synthesizes ferroptosis pathophysiology and research advances but also delineates disease-tailored therapeutic strategies. By addressing key knowledge gaps—crosstalk between ferroptosis and other cell death modalities (e.g., pyroptosis), lack of kidney-specific clinical biomarkers, and underexplored roles in autoimmune nephritides—it provides a conceptual roadmap for mechanism-based diagnostics, precision therapeutics, and rational drug combinations, transcending traditional disease boundaries to advance clinical translation for both primary and secondary kidney diseases.

## Highlights

- This article connects ferroptosis to various major kidney diseases, highlighting the critical role of ferroptosis in the onset and progression of renal disorders.
- An in-depth analysis of the molecular mechanisms of ferroptosis reveals multiple potential therapeutic targets and diverse regulatory pathways, providing directions for novel therapeutic strategies in kidney disease treatment.
- Multiple cell death pathways are involved in the pathogenesis of acute and chronic kidney injury; ferroptosis is one such mechanism regulating necrotic cell death, characterized by the pronounced oxidation of phospholipids containing polyunsaturated fatty acids.
- In cells lacking glutathione peroxidase 4 (GPX4) catalytic activity, iNOS, NO•, Ca^2+^-independent phospholipase A2β, and FSP1 can eliminate lipid peroxidation products associated with ferroptosis, thereby preventing ferroptosis. Elevated urinary levels of ferroptosis-specific phospholipid hydroperoxides are associated with non-recovery of renal function in patients with AKI.
- The role of ferroptosis in the pathogenesis of kidney diseases suggests its potential as a therapeutic target. Careful consideration of the phospholipid peroxidation process and its underlying mechanisms is required when designing and developing ferroptosis-selective inhibitors that do not interfere with other critical regulatory cascades.

## 1 Introduction

Kidney disease is a major global non-communicable disease (NCD), with a global prevalence exceeding 10% [affecting ~850 million people when including acute kidney injury (AKI), kidney failure, and dialysis/transplant recipients] (1, 2). It is the third fastest-growing cause of death worldwide—unique among NCDs in that mortality rises with age—and is projected to become the fifth leading cause of life-years lost globally by 2040 (3). CKD, the most prevalent form, progresses irreversibly to kidney failure, requiring renal replacement therapy (RRT; dialysis or transplantation) for survival. However, RRT is non-curative, leaving kidney failure-associated mortality and morbidity high; further, limited early detection programs in many regions lead to the significant underestimation of early-stage CKD burden (4, 5). Understanding CKD pathogenesis is critical to addressing this gap, with key mechanisms driving irreversible renal parenchyma damage (fibrosis, tubular atrophy, and interstitial fibrosis) including intravascular neutrophil extracellular trap formation (NETosis), immunothrombosis, endothelial/mesangial cell proliferation, glomerular leukocyte infiltration, capillary loop necrosis, periglomerular lymphocyte infiltration, type I interferon-induced podocyte death, and programmed cell death (6–8).

Cell death is a finely regulated process that occurs through various molecular pathways (9). Ferroptosis, a novel form of cell death, was first proposed by Dixon et al. in 2012. It is an iron-dependent, non-apoptotic form of cell death characterized by the accumulation of intracellular iron and lipid peroxidation (10). The discovery of this mode of cell death originated from studies on RAS-mutant cancer cells, particularly investigations involving the compound erastin. Erastin selectively kills cancer cells expressing RAS, but its mechanism of cell death differs from previously observed patterns: there are no changes in nuclear morphology, DNA fragmentation, or caspase activation, and the process cannot be reversed by caspase inhibitors (11). Subsequently, Yang (12) and Yagoda (13) identified another compound, RSL3, which also induces this mode of cell death. Based on its characteristics—namely, the accumulation of iron and lipid peroxidation during cell death—this process was formally named ferroptosis. Iron, as an essential trace element in the human body, plays a critical role in fundamental biological processes such as energy metabolism, redox balance, oxygen transport, and inflammatory responses (14). However, excessive free iron promotes the production of free radicals and participates in the pathological progression of various chronic diseases. Therefore, systemic iron homeostasis exerts pleiotropic effects on renal function and the progression of kidney diseases (15).

**Figure 1:**
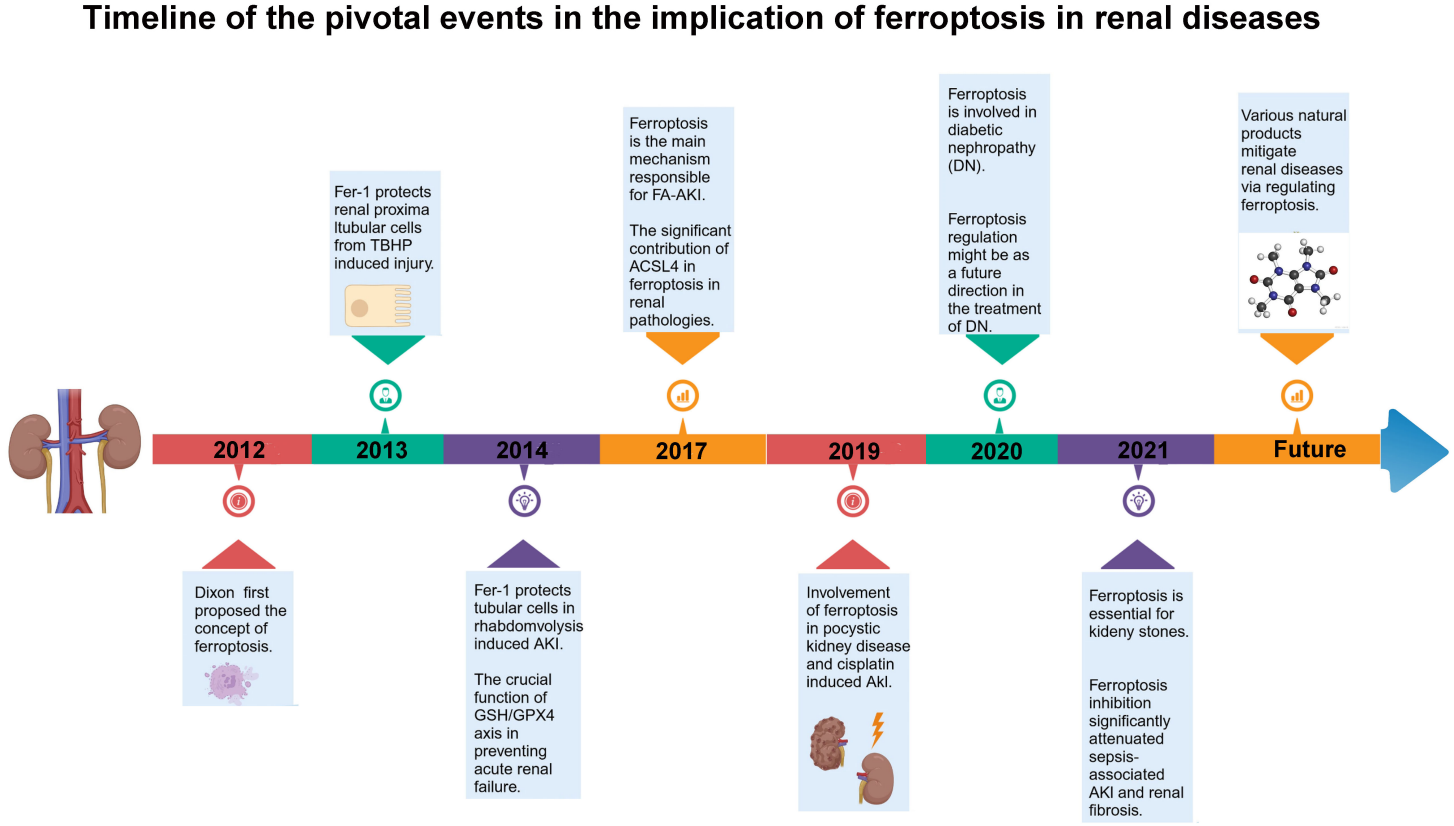
Timeline of ferroptosis in kidney diseases: a chronological overview of key events involving ferroptosis in various renal disorders. TBHP, *tert*-butyl hydroperoxide; AKI, acute kidney injury; GSH, glutathione; GPX4, glutathione peroxidase 4; FA-AKI, folic acid-induced acute kidney injury; ACSL4, acyl-coenzyme A synthetase long-chain family member 4; DN, diabetic nephropathy.

Recent studies have confirmed that the kidney is particularly susceptible to redox imbalance, and ferroptosis plays a significant role in the pathophysiology of various kidney diseases, emerging as a new research hotspot in the field of renal fibrosis (16). The renal tubules, a critical component of the kidney, are vulnerable to damage caused by factors such as hypoxia, toxins, metabolic disorders, and aging. In acute kidney disease, redox imbalance also occurs in AKI, leading to impaired defense systems such as mitophagy and triggering cell death programs like ferroptosis (17). Following injury, renal tubular epithelial cells (TECs) undergo morphological changes and secrete bioactive molecules, driving interstitial inflammation and fibrosis. Ferroptosis can also lead to the release of intracellular molecules with immunogenic and damage-associated molecular pattern (DAMP) functions, thereby promoting kidney injury and disease progression, ultimately resulting in the development of CKD and end-stage renal disease (ESRD) (18). Accumulating evidence suggests a link between ferroptosis and AKI induced by various stimuli such as ischemia–reperfusion, sepsis, or toxins, as well as its association with chronic kidney disease, indicating that ferroptosis may represent a novel therapeutic target for kidney diseases (19, 20). A detailed temporal overview of the key events involved in ferroptosis across different kidney diseases is illustrated in **Figure 1**. Therefore, in this review, we discuss the fundamental mechanisms of ferroptosis and its relationship with the pathophysiology of various kidney diseases, and we describe potential therapeutic approaches targeting ferroptosis.

## 2 Ferroptosis

At the cytological level of ferroptosis (20), cells undergoing ferroptosis typically exhibit morphological changes similar to necrosis, including loss of plasma membrane integrity, cytoplasmic swelling (oncosis), organelle swelling, and moderate chromatin condensation. In some cases, ferroptosis is also accompanied by cell detachment, rounding, and an increase in autophagosomes. At the ultrastructural level, ferroptosis often displays mitochondrial abnormalities, such as mitochondrial shrinkage or swelling, increased membrane density, reduced or absent cristae, and rupture of the outer membrane. Despite these significant changes in mitochondrial morphology, the role of mitochondria in ferroptosis remains controversial. At the biochemical level (21, 22), ferroptosis is associated with two major biochemical hallmarks: iron accumulation and lipid peroxidation. Activators of ferroptosis, such as erastin or RSL3, increase intracellular iron accumulation by inhibiting antioxidant systems. Iron stimulates the formation of reactive oxygen species (ROS) through the Fenton reaction, oxidizing phospholipids containing unsaturated fatty acid tails, thereby initiating lipid peroxidation. The degree of lipid unsaturation of glutathione (GSH) determines the sensitivity to ferroptosis. Cells primarily rely on two antioxidant systems to catalyze the reduction of lipid peroxides: the GSH/peroxidase 4 (GPX4) system and the coenzyme Q10 (CoQ10)/ferroptosis suppressor protein 1 (FSP1) system (22). Detecting changes in the expression and activity of these molecules is crucial for further research into ferroptosis.

### 2.1 Iron metabolism regulates ferroptosis

**Figure 2:**
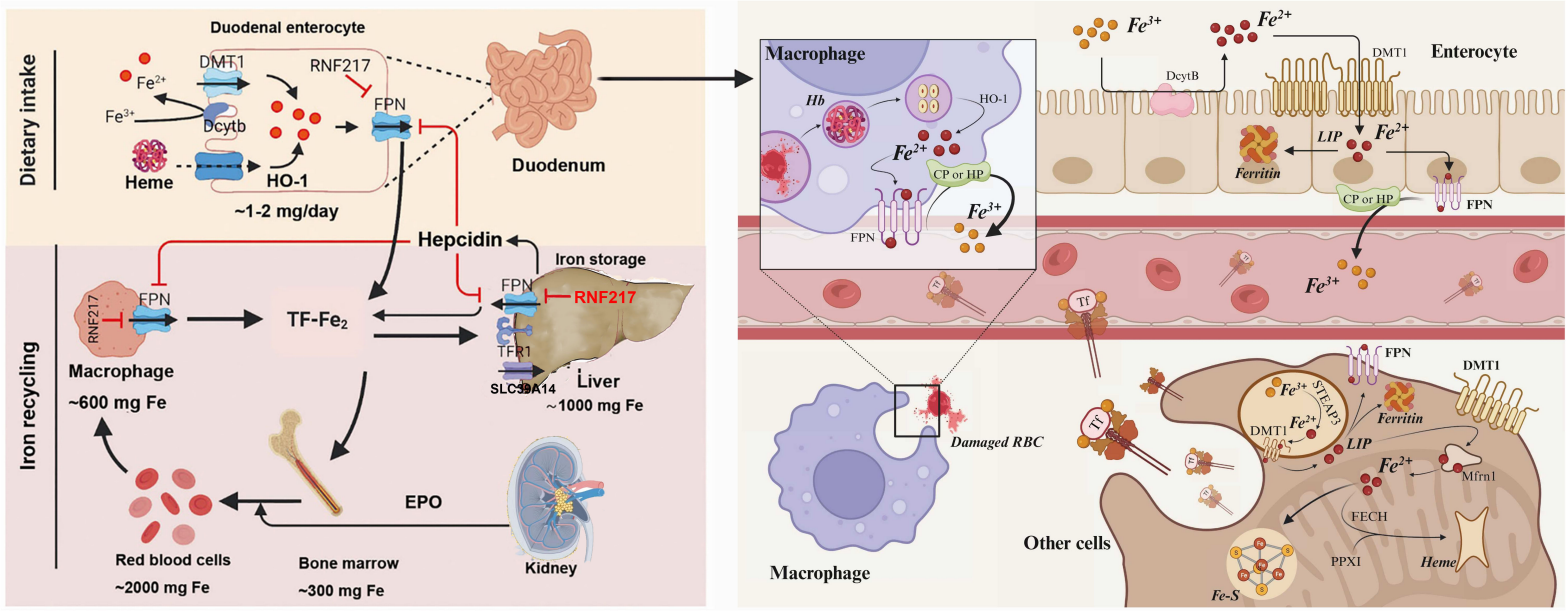
Iron homeostasis and cellular iron metabolism. Following dietary iron intake, Fe^3+^ is reduced by duodenal cytochrome *b* (DcytB) and subsequently transported into enterocytes via divalent metal transporter 1 (DMT1). Dietary heme is absorbed through an as-yet-undefined mechanism and degraded within enterocytes by heme oxygenase-1 (HO-1). Iron is exported from enterocytes, macrophages, and hepatocytes via the iron exporter ferroportin (FPN)—the sole known cellular iron efflux protein—and binds to transferrin in the plasma as ferrous iron (TF–Fe^2+^), forming diferric transferrin, which delivers iron to tissues, primarily for *de novo* hemoglobin synthesis in erythroid precursors. Macrophages recycle iron by phagocytosing senescent red blood cells and catabolizing heme through HO-1, releasing iron back into the circulation. When needed, erythropoietin (EPO), released by the kidneys, promotes erythropoiesis via the hypoxia-inducible factor (HIF) signaling pathway. Iron utilization in the bone marrow and its recycling by macrophages constitute the major physiological iron cycle. Excess iron is stored in hepatocytes via transferrin receptor 1 (TFR1)-mediated uptake of TF–Fe^2+^ or through uptake of non-transferrin-bound iron (NTBI) involving SLC39A14. Systemic iron homeostasis is primarily regulated by hepcidin, a peptide hormone synthesized in hepatocytes. Recently, we identified RNF217 as a novel E3 ubiquitin ligase that mediates FPN degradation, thereby modulating iron efflux. In the duodenum and jejunum, Fe^2+^ can enter cells directly via DMT1, whereas Fe^3+^ must first be reduced to Fe^2+^ by DcytB before transport via DMT1 into the labile iron pool (LIP). A fraction of intracellular Fe^2+^ is exported to the circulation through membrane-localized FPN, where it is oxidized to Fe^3+^ by ceruloplasmin (CP) or hephaestin (Hp), and then loaded onto plasma TF for systemic distribution. The remainder is stored intracellularly in ferritin (Ftn). Transferrin-bound Fe^3+^ is internalized via transferrin receptor 1 (TFR1)-mediated endocytosis and released into endosomal compartments, where STEAP3 reduces Fe^3+^ to Fe^2+^, enabling its transport into the cytosol via DMT1 to replenish the LIP. Cytosolic labile iron can also be transported across the mitochondrial inner membrane via mitoferrin-1 (Mfrn1) for utilization in mitochondrial iron–sulfur (ISC) cluster biogenesis and heme synthesis. Within the mitochondria, iron is inserted into protoporphyrin IX (PPIX) by ferrochelatase (FECH) to generate heme. Macrophages engulf aged or damaged erythrocytes, degrade heme via HO-1, and release free iron, which is either reused or stored, thus completing the iron recycling loop.

Less than 1% of the body’s total iron exists extracellularly; most intracellular iron (>90%) in mammalian cells is present as a cofactor in heme (also known as ferrous heme), iron–sulfur clusters, and mono- or di-iron centers in enzymes. Iron supply primarily depends on dietary intake (23). Iron is absorbed by duodenal epithelial cells, and apart from the small intestine, the kidneys, liver, and macrophages also play significant roles in systemic iron homeostasis. Iron filtered by the glomerulus is actively reabsorbed to prevent urinary loss. Plasma iron levels are regulated by hepcidin, a peptide hormone predominantly synthesized by hepatocytes in the liver, with minor synthesis occurring in other cells such as macrophages and epithelial cells of the distal renal tubules (24). Hepcidin binds to ferroportin (distinct from transferrin, as ferroportin transports Fe^2+^, while transferrin in portal blood only binds Fe^3+^), leading to its degradation in enterocytes and macrophages, resulting in intracellular iron retention and reduced circulating iron levels. The synthesis of hepcidin is regulated by circulating iron levels, inflammatory stimuli such as IL-6, iron storage, and erythropoiesis. Erythropoietin (EPO), produced by the kidneys, stimulates erythropoiesis and the synthesis of erythroferrone in the bone marrow. Erythroferrone then inhibits hepcidin synthesis in hepatocytes. Iron can also be absorbed as heme by various cells, including enterocytes, hepatocytes, and macrophages, primarily through heme carrier protein 1 (HCP1) or heme-responsive gene 1 (HRG1) (25). Iron is an essential trace element for the survival of almost all organisms, participating in the synthesis of iron–sulfur clusters and heme, as well as other physiological activities. Maintaining its homeostasis is crucial for normal cellular function (26). The occurrence of ferroptosis is iron-dependent, and the regulation of iron metabolism in ferroptosis hinges on the control of the labile iron pool (LIP). Under physiological conditions, transferrin receptor 1 (TFR1) binds to transferrin (TF), mediating cellular iron uptake via endocytosis for the synthesis of heme, iron–sulfur clusters, or storage in ferritin. When TFR1 expression on the cell membrane is upregulated, more TF is recognized and bound, increasing the amount of Fe^3+^ entering the endosome. Excess Fe^3+^ is reduced to Fe^2+^ by the metalloreductase STEAP3 and transported into the cytoplasm via ZRT/IRT-like protein 14 (ZIP14) and solute carrier family 11 member 2 (SLC11A2/DMT1), forming the LIP and leading to iron metabolic disorders (27). Increased iron uptake elevates intracellular LIP, which reacts with hydrogen peroxide (H_2_O_2_) through the Fenton reaction to produce large amounts of hydroxyl radicals. These radicals cause oxidative damage to proteins, lipids, and DNA, increasing cellular sensitivity to ferroptosis (28). Another mechanism leading to LIP formation is ferritinophagy, initially proposed by Mancias et al., which is a selective autophagy process that releases iron by lysosomal degradation of ferritin (29). Ferritin, present in both the cytoplasm and mitochondria, oxidizes Fe^2+^ to Fe^3+^ and stores it within protein subunit complexes. Ferritinophagy releases iron through its selective cargo receptor, nuclear receptor coactivator 4 (NCOA4), providing bioavailable iron for cellular and mitochondrial functions. When NCOA4 expression is upregulated, it recognizes and binds to ferritin heavy chain 1 (FTH1) and mitochondrial ferritin (FTMT), interacting with autophagy-related factors and primary autophagosomes to form autophagosomes for lysosomal degradation, thereby releasing large amounts of Fe^2+^ and forming the LIP. This leads to cellular iron overload and induces ferroptosis (30). Studies have confirmed that the depletion of ferritinophagy results in intracellular iron exhaustion and reduced lipid peroxidation, promoting cell survival during erastin-induced ferroptosis (31). Additionally, Fe^2+^ can be released from heme through the activation of heme oxygenase-1 (HO-1), thereby accelerating ferroptosis (8). It can also increase the activity of lipoxygenases (LOXs), catalyzing the peroxidation of polyunsaturated fatty acids (PUFAs) and promoting the onset of ferroptosis (32). Despite these advances in elucidating iron metabolic pathways underlying ferroptosis, critical knowledge gaps persist that limit both mechanistic completeness and translational potential. First, the hierarchical priority of distinct iron metabolic pathways (e.g., TFR1-mediated uptake *vs*. ferritinophagy *vs*. heme degradation) in regulating LIP and ferroptosis remains undefined across cell types, tissues, and pathological contexts; current studies have often focused on individual pathways in isolation, lacking comparative analyses to identify context-dependent key regulators. Second, the direct crosstalk between systemic iron homeostasis (e.g., the hepcidin–ferroportin axis) and intracellular ferroptosis signaling is poorly characterized; how systemic iron disorders (e.g., iron overload or deficiency diseases) modulate cellular ferroptosis susceptibility, and vice versa, has not been systematically addressed. Third, mitochondrial iron metabolism—beyond the role of FTMT and ferritinophagy—remains an understudied frontier; the specific mechanisms governing mitochondrial LIP formation, its crosstalk with cytoplasmic LIP, and its unique contribution to ferroptosis (distinct from cytoplasmic iron overload) are largely unresolved. Finally, the functional redundancy or compensation between iron metabolic regulators in ferroptosis (e.g., whether inhibition of TFR1 can be rescued by enhanced ferritinophagy) has not been rigorously evaluated, which is essential for designing robust therapeutic strategies targeting iron-dependent ferroptosis. Filling these gaps will require integrated, multi-dimensional studies to reconcile cell-autonomous and systemic iron regulation and to establish a more comprehensive framework for ferroptosis modulation. Iron absorption and metabolism are illustrated in **Figure 2**.

### 2.2 Lipid metabolism and antioxidant systems regulate ferroptosis

Lipid synthesis and catabolism directly or indirectly influence lipid peroxidation in ferroptosis. PUFAs are among the primary targets of lipid peroxidation, and lipid synthesis-mediated production of PUFAs increases cellular sensitivity to ferroptosis (33). The production of PUFAs requires the activation of the ACSL4-LPCAT3 (acyl-CoA synthetase long-chain family member 4-lysophosphatidylcholine acyltransferase 3) pathway. ACSL4 is an enzyme that converts fatty acids into acyl-CoA esters, a lipid metabolism enzyme essential for lipid peroxidation and a key participant in ferroptosis (34, 35). Upon activation, ACSL4 catalyzes the binding of CoA to PUFAs such as arachidonic acid and adrenic acid through LPCAT3, forming acyl-CoA and participating in lipid signaling during ferroptosis. This promotes the esterification of arachidonic acid and adrenic acid into phospholipids, leading to ferroptosis (33). Lipids can also be peroxidized by LOXs, cytochrome P450 enzymes (CYP/CYP450), and prostaglandin-endoperoxide synthases (PTGS/COX). Arachidonate lipoxygenase (ALOX) is a non-heme iron dioxygenase that primarily mediates ROS-induced lipid peroxidation, inducing ferroptosis by oxidizing PUFA–phosphatidylethanolamine (PUFA–PE). Studies have shown that phosphatidylethanolamine-binding protein 1 (PEBP1) can act as an adaptor protein for ALOX15, enhancing its activity in promoting ferroptosis (36). Recent studies have identified FSP1 and dihydroorotate dehydrogenase (DHODH) as key regulators of ferroptosis. FSP1 and DHODH reduce coenzyme Q (CoQ) to dihydrocoenzyme Q (CoQH_2_) on the plasma membrane and mitochondrial inner membrane, respectively. CoQH_2_ neutralizes lipid peroxidation-derived ROS, acting as a radical-trapping antioxidant to prevent lipid peroxidation and thereby inhibit ferroptosis (37, 38). FSP1-mediated production of CoQ10 inhibits lipid peroxidation (39). The depletion of CoQ10 increases cellular sensitivity to ferroptosis. Statins promote lipid peroxidation and induce ferroptosis by inhibiting HMG-CoA (3-hydroxy-3-methylglutaryl coenzyme A) reductase, which blocks mevalonate-derived CoQ10 synthesis (40). Additionally, NADPH oxidases (NOXs) are a family of enzymes that produce ROS, using NADPH as an electron donor and oxygen as an electron acceptor to catalyze the reduction of oxygen molecules into superoxide or peroxide (41). NOX4 induces lipid peroxidation through oxidative stress, promoting hydrogen peroxide ferroptosis (42). Superoxide dismutase (SOD), an antioxidant metalloenzyme, catalyzes the dismutation of superoxide anion radicals into oxygen and H_2_O_2_. Catalase (CAT), an enzymatic scavenger, removes intracellular H_2_O_2_, protecting cells from H_2_O_2_ toxicity. These enzymes play crucial roles in combating oxidative stress and lipid peroxidation, thereby suppressing ferroptosis. Nevertheless, substantial gaps in current research hinder a comprehensive understanding of lipid metabolism and antioxidant system-mediated ferroptosis regulation, as well as their translational application. First, the hierarchical coordination between distinct lipid metabolic pathways (e.g., ACSL4-LPCAT3-mediated PUFA esterification *vs*. LOX/CYP450-driven peroxidation) remains unclear; whether there is a rate-limiting pathway or synergistic/antagonistic crosstalk across cell types and pathological conditions has not been systematically evaluated. Second, the functional contribution of non-PUFA lipids (e.g., sphingolipids and cholesterol esters) to ferroptosis is largely understudied; their potential as alternative peroxidation substrates or regulators of PUFA-dependent lipid peroxidation has not been rigorously explored. Third, the subcellular compartmentalization of antioxidant defenses against ferroptosis is incomplete; beyond the plasma membrane (FSP1) and mitochondria (DHODH), the specific antioxidant mechanisms in organelles such as the endoplasmic reticulum (a major site of lipid synthesis) and peroxisomes (involved in lipid oxidation) are poorly defined, and their crosstalk with cytoplasmic SOD/CAT remains elusive. Finally, clinical translation is impeded by the lack of disease-specific profiles of lipid metabolism and antioxidant system dysregulation; the potential off-target effects of therapies targeting these pathways (e.g., disrupting normal lipid homeostasis in healthy tissues) have not been adequately addressed, and biomarkers to predict ferroptosis susceptibility via lipid/antioxidant signatures are still lacking. Addressing these gaps will require integrated multi-omics and subcellular-resolution studies to establish a more precise and context-dependent regulatory framework for ferroptosis.

### 2.3 Amino acid metabolism and antioxidant systems regulate ferroptosis

Abnormal amino acid metabolism leads to the inactivation of antioxidant systems, and the accumulation of lipid peroxidation products is regulated by antioxidant systems, which are considered critical determinants of whether ferroptosis occurs. The cystine/glutamate antiporter system (xCT), a disulfide-linked heterodimer composed of SLC7A11 and SLC3A2, serves as the pathway for cystine uptake into cells, where it is reduced to cysteine for the synthesis of intracellular GSH. GPX4, a selenoenzyme that reduces phospholipid hydroperoxides (PLOOH) via GSH, prevents lipid peroxidation-induced ferroptosis (43). Early studies demonstrated that the ferroptosis inducer erastin disrupts the GSH–GPX4-dependent antioxidant system by inhibiting xCT, reducing cystine influx, and leading to insufficient intracellular GSH levels. This results in the inactivation of the lipid repair enzyme GPX4, preventing it from exerting its normal antioxidant capacity and thereby inducing ferroptosis (44). Glutamine, produced by the enzymatic action of glutamate-ammonia ligase (GLUL), is essential for cell growth. Studies have identified glutamine as a key regulator of ferroptosis (45). Cystine deprivation and glutaminolysis increase mitochondrial α-ketoglutarate dehydrogenase (α-KGDH) activity and Fe^2+^ levels, promoting lipid peroxidation, iron accumulation, and reactive oxygen species (45). Under conditions of abnormal amino acid metabolism, cell membranes containing phospholipids are highly susceptible to oxidative attack by ROS, generating end-products of lipid peroxidation such as malondialdehyde (MDA) and 4-hydroxynonenal (4-HNE). These compounds readily form adducts with proteins and DNA, exhibiting significant cytotoxicity and inducing ferroptosis (22, 46). Despite the established roles of cystine and glutamine metabolism in regulating the GSH–GPX4 axis and ferroptosis, critical knowledge gaps persist that limit a holistic understanding of amino acid-dependent ferroptosis regulation and its translational potential. First, the bidirectional crosstalk between the xCT-GSH–GPX4 pathway and glutamine metabolism remains poorly defined; whether they function as independent modules or synergistically/antagonistically tune ferroptosis susceptibility, and how this coordination is rewired across cancer subtypes, degenerative diseases, or inflammatory contexts, has not been systematically interrogated. Second, the contribution of other amino acids (e.g., glycine, serine, or glutamate beyond glutaminolysis) to ferroptosis regulation is largely underexplored; their potential to modulate GSH synthesis, iron homeostasis, or lipid peroxidation directly or indirectly has not been rigorously evaluated, leaving a gap in the amino acid-ferroptosis regulatory network. Third, the subcellular compartmentalization of amino acid metabolism-linked antioxidant defense is incomplete; whether mitochondrial or endoplasmic reticulum-localized amino acid metabolic enzymes (e.g., mitochondrial glutaminase) independently regulate local ferroptosis signaling, and how these subcellular pathways crosstalk with cytoplasmic GSH–GPX4, remains elusive. Finally, clinical translation is hindered by the lack of robust biomarkers that reflect amino acid metabolism-antioxidant system dysregulation in ferroptosis-related diseases; moreover, the off-target effects of therapies targeting xCT or glutaminolysis (e.g., disrupting essential amino acid homeostasis in normal tissues) have not been adequately characterized, and strategies to enhance therapeutic specificity are still lacking. Addressing these gaps will require integrated metabolomic, proteomic, and subcellular-resolution studies to establish a context-dependent, multi-dimensional regulatory framework for amino acid-mediated ferroptosis.

### 2.4 Mitochondrial pathways

Mitochondria, the energy-producing centers of cells, play a role in regulating energy metabolism, lipid metabolism, and iron metabolism (47). Specific alterations in mitochondria are also important characteristics that distinguish ferroptosis from other forms of cell death, including mitochondrial double-membrane rupture, reduced mitochondrial volume, and decreased or absent mitochondrial cristae (48). It has been discovered that mitochondria can drive or suppress ferroptosis through various mechanisms. Mitochondria are a major source of ROS, and during oxidative phosphorylation, the electron transport chain generates large amounts of ROS. When the mitochondrial antioxidant system is impaired, excessive ROS can lead to lipid peroxidation via the Fenton reaction, inducing ferroptosis (49). Voltage-dependent anion channels (VDACs), channel proteins located on the outer mitochondrial membrane, can be activated by the ferroptosis inducer erastin, promoting their opening. This results in increased mitochondrial membrane potential and excessive ROS production, leading to calcium overload and ultimately mitochondrial energy depletion and damage (50). DHODH, an enzyme located on the inner mitochondrial membrane, oxidizes dihydroorotate to orotate while reducing CoQ to CoQH_2_, thereby scavenging mitochondrial oxygen radicals and exerting anti-ferroptotic effects. The inactivation of DHODH exacerbates ferroptosis (38). FTMT regulates mitochondrial iron metabolism and inhibits oxidative damage (51). Studies have shown that increasing FTMT expression enhances mitochondrial iron storage, thereby reducing cellular ROS levels and suppressing erastin-induced ferroptosis (52). Despite growing insights into the mitochondrial regulation of ferroptosis, critical knowledge gaps remain that impede a comprehensive mechanistic understanding and translational progress. First, the hierarchical coordination among distinct mitochondrial ferroptosis regulators (e.g., ROS production, VDACs, DHODH, and FTMT) is undefined; whether these factors act sequentially, synergistically, or antagonistically across cell types, tissues, or pathological contexts (e.g., cancer *vs*. ischemia–reperfusion injury) has not been systematically dissected. Second, the role of understudied mitochondrial structures and processes in ferroptosis is largely unaddressed; for example, mitochondrial autophagy (mitophagy) mediated by PINK1/Parkin, mitochondrial dynamics (fusion/fission), or mitochondrial DNA (mtDNA) damage—all of which are linked to oxidative stress—have not been rigorously evaluated for their direct contribution to ferroptosis initiation or suppression. Third, the cross-compartmental crosstalk between mitochondria and the cytoplasm/nucleus in ferroptosis signaling is poorly characterized; how mitochondrial-derived signals (e.g., specific lipids, peptides, or ROS subsets) propagate to regulate cytoplasmic iron metabolism, lipid peroxidation, or antioxidant systems remains elusive. Finally, clinical translation is hindered by the lack of mitochondria-targeted ferroptosis modulators with high specificity; current strategies often disrupt normal mitochondrial function in healthy tissues, and biomarkers to stratify patients likely to respond to such therapies (based on mitochondrial ferroptosis signatures) are still absent. Addressing these gaps will require integrated structural biology, subcellular proteomics, and preclinical studies to establish a mitochondria-centric, context-dependent framework for ferroptosis regulation.

### 2.5 p53 pathway

p53 is a critical tumor suppressor that regulates cell growth, apoptosis, and DNA repair. Mutations in the p53 gene result in a loss of control over cell proliferation, leading to cellular transformation and cancer (53). Recent studies have revealed that p53 plays a dual role in ferroptosis, either promoting or inhibiting ferroptosis by regulating different target genes. p53 can directly bind to the promoter region of SLC7A11 in relevant cells, suppressing its transcriptional activity, thereby inhibiting cystine uptake, reducing GSH synthesis, and inducing ferroptosis (54, 55). The p53 target gene spermidine/spermine N1-acetyltransferase 1 (SSAT1) can activate the expression of ALOX15, increase ROS production, trigger lipid peroxidation, and induce cell death (56). Conversely, p53 also exhibits an inhibitory effect on ferroptosis. Dipeptidyl peptidase-4 (DPP4), which interacts with NADPH oxidase 1 to promote lipid peroxidation, can form a complex with p53. This interaction facilitates the nuclear translocation of DPP4, thereby preventing lipid peroxidation and suppressing ferroptosis (57). Increased p53 activity has been shown to mitigate erastin-induced ferroptosis in fibrosarcoma, renal cell carcinoma (RCC), and osteosarcoma (58). Despite the recognition of p53’s dual role in ferroptosis, critical knowledge gaps and unresolved contradictions hinder a precise mechanistic framework and translational application. First, the context-dependent regulatory switch that dictates whether p53 promotes or inhibits ferroptosis remains undefined; how cellular microenvironments (e.g., nutrient availability and oxidative stress levels), tissue types, or cancer subtypes toggle p53’s interaction with target genes (SLC7A11/SSAT1 *vs*. DPP4) has not been systematically elucidated. Second, the crosstalk between p53 and other ferroptosis-regulating pathways (e.g., iron metabolism, lipid peroxidation machinery, and mitochondrial signaling) is poorly characterized; whether p53 acts upstream of these pathways, converges with them, or is modulated by them to exert its dual effects remains largely unexplored. Third, the role of p53 mutations (the most frequent mutations in human cancers) in ferroptosis is largely overlooked; current studies have focused primarily on wild-type p53, while the impact of hotspot p53 mutations (e.g., R175H and R273H) on ferroptosis susceptibility, and whether mutant p53 retains or rewires the dual regulatory function, is unknown. Finally, translational progress is bottlenecked by the lack of strategies to selectively manipulate p53’s ferroptosis-regulating activity: activating its pro-ferroptotic function in cancer cells while preserving its anti-ferroptotic role in normal tissues remains a major challenge, and no validated biomarkers exist to predict which tumors will respond to p53-targeted ferroptosis therapies. Addressing these gaps requires integrated genomic, proteomic, and preclinical studies to dissect the molecular determinants of p53’s dual role and resolve its clinical utility in ferroptosis-based cancer treatment.

### 2.6 Other mechanisms

**Figure 3:**
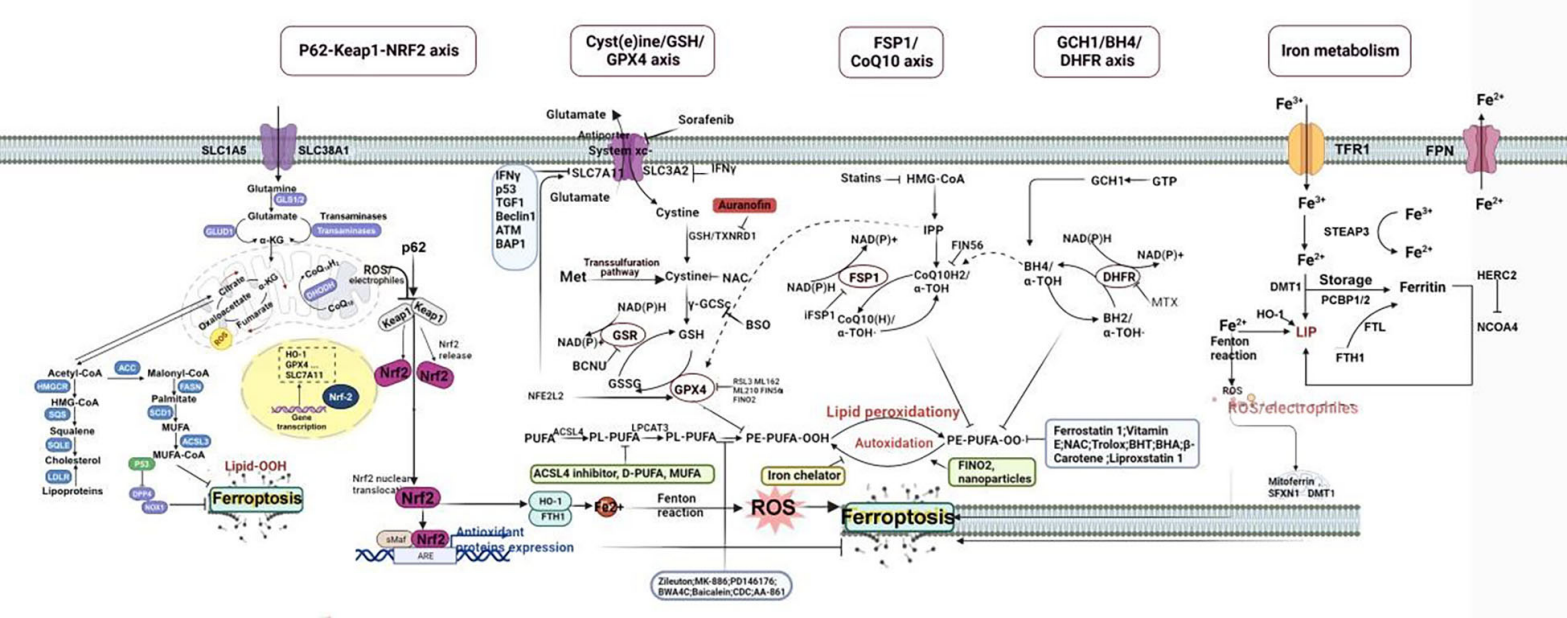
Molecular mechanisms of ferroptosis. This schematic illustrates the metabolic pathways associated with iron-dependent cell death. Iron-dependent lipid peroxidation drives ferroptosis at the cellular level. Multiple aspects of iron metabolism—including iron absorption, storage, and utilization—play critical roles in regulating ferroptotic sensitivity. In lipid metabolism, activation of ACSL4, lysophosphatidylcholine acyltransferase 5 (LPCAT3, also known as LPLAT5), lipoxygenases (LOX), or NADPH oxidases (NOXs) promotes the peroxidation of phospholipids containing polyunsaturated fatty acids (PUFA-PL), thereby facilitating ferroptosis. The canonical ferroptosis suppression pathway involves the cystine–glutamate antiporter System Xc^−^, which imports cystine (Cys) to support the synthesis of glutathione (GSH). As a cofactor, GSH enables glutathione peroxidase 4 (GPX4) to reduce phospholipid hydroperoxides to their corresponding alcohols, thereby preventing lethal lipid peroxidation. Phospholipid peroxidation can also be inhibited by the FSP1–CoQ10 system. Furthermore, ferroptosis is tightly regulated by cellular iron homeostasis, encompassing iron uptake, transport, storage, and utilization. At the cellular level, non-heme iron enters the cell either through transferrin receptor 1 (TFR1)-mediated uptake of transferrin (TF)-bound iron or via transferrin-independent iron import mediated by solute carrier family 39 member 14 (SLC39A14, also known as ZIP14). Additionally, heme degradation and NCOA4-mediated ferritinophagy increase the labile iron pool (LIP), rendering cells more susceptible to ferroptosis through Fenton chemistry. FPN, ferritin; Glu, glutamate; GSSG, oxidized glutathione; HO-1, heme oxygenase-1; KEAP1, Kelch-like ECH-associated protein 1; NRF2, nuclear factor erythroid 2-related factor 2; PUFA, polyunsaturated fatty acid; PUFA-CoA, polyunsaturated fatty acyl–coenzyme A; PUFA-PL, polyunsaturated fatty acid-containing phospholipid; STEAP3, six-transmembrane epithelial antigen of the prostate 3 (a metalloreductase).

Different types of cell death can induce distinct immune and inflammatory responses by releasing and activating DAMP signals. Ferroptosis is associated with the release of DAMPs (59). The lipid peroxidation product 4-HNE can activate nuclear factor kappa-B (NF-κB), triggering inflammatory responses (60). Additionally, evidence suggests that ferroptosis crosstalks with other forms of programmed cell death, such as apoptosis, autophagy, and pyroptosis, indicating interactions between ferroptosis and these cell death pathways (61, 62). During autophagy, ferritin degradation releases iron, further exacerbating ferroptosis. Oxidative stress and lipid peroxidation are also linked to the activation of apoptotic signaling. A recent study (63) demonstrated that ferroptosis propagates in a ROS-triggered wave-like manner, enabling large-scale cell death within cell populations. This study also revealed the potential mechanisms of ferroptosis in cellular remodeling and organogenesis, providing new insights into the interplay between developmental signaling and ferroptosis. Several key regulatory points and potential targets have been identified, including strategies to inhibit iron uptake, modulate antioxidant systems, and prevent lipid peroxidation (64). Two recent studies in *Nature* (65, 66) simultaneously identified 7-dehydrocholesterol (7-DHC) as a potent anti-ferroptotic metabolite and an endogenous inhibitor of ferroptosis. 7-DHC mitigates ferroptosis by diverting the peroxidation pathway from phospholipids, protecting cells from phospholipid peroxidation on cell membranes and mitochondria. However, high levels of 7-DHC can lead to more aggressive cancer phenotypes and promote cancer metastasis. Additionally, ergosterol, a 7-DHC analog, was found to inhibit ferroptosis. These findings reveal a novel mechanism for regulating ferroptosis and hold significant implications for developing treatments for ferroptosis-related diseases, such as cancer and ischemia–reperfusion injury (67). Current research shows that phospholipid peroxidation occurs spontaneously during normal cellular metabolism and stress responses. Under physiological conditions, cells require surveillance mechanisms to resist unintended ferroptosis triggered by phospholipid peroxidation. Two known regulatory mechanisms for monitoring ferroptosis have been identified: 1) GPX4 mediates the reduction of phospholipid hydroperoxides (PL-OOH) to their corresponding phospholipid alcohols (PL-OH) (68); and 2) enzymes such as FSP1, DHODH, NOS2, MBOAT1/2, and GCH1 produce radical-trapping antioxidants (RTAs, e.g., CoQ10, NO, and BH4), which terminate phospholipid peroxidation to block ferroptosis (38, 69–71). These surveillance mechanisms are highly active in various cancer cells, allowing them to evade ferroptosis. Inducing ferroptosis by inhibiting these surveillance mechanisms is a current research focus in cancer therapy. Discovering ferroptosis-monitoring mechanisms independent of GPX4 and RTAs will provide critical guidance for developing combination therapies aimed at inducing ferroptosis in tumors. Furthermore, the use of iron chelators or specific ferroptosis inhibitors can effectively block ferroptosis. In summary, ferroptosis is inseparably linked to intracellular iron overload and lipid peroxidation. Iron accumulation and lipid deposition create favorable conditions for the occurrence of ferroptosis, while oxidative stress promotes lipid peroxidation. The mechanisms underlying ferroptosis induced by different pathological factors may vary, meaning that the mechanisms of ferroptosis occurrence may differ to some extent across different diseases. In summary, ferroptosis is inseparably linked to intracellular iron overload and lipid peroxidation. Iron accumulation and lipid deposition create favorable conditions for the occurrence of ferroptosis, while oxidative stress promotes lipid peroxidation. The mechanisms underlying ferroptosis induced by different pathological factors may vary, meaning that the mechanisms of ferroptosis occurrence may differ to some extent across different diseases. However, critical gaps remain in translating these mechanistic insights into clinical applications and a comprehensive understanding. First, the specific molecular switches that determine the divergence of ferroptosis pathways across distinct diseases (e.g., cancer subtypes and ischemia–reperfusion injury in different organs) have not been systematically delineated, leading to a lack of disease-specific therapeutic targets. Second, the dual role of metabolites like 7-DHC—simultaneously inhibiting ferroptosis and promoting cancer metastasis—poses a major conundrum, yet the regulatory networks that balance these opposing effects remain poorly defined, hindering safe therapeutic development. Third, non-GPX4/non-RTA ferroptosis surveillance mechanisms are largely underexplored; current studies are scattered and lack a unified framework to classify their functional hierarchy or crosstalk with known pathways. Finally, the causal relationship between iron overload and lipid peroxidation in pathological contexts is often assumed rather than rigorously validated, with limited understanding of how other cellular stressors (e.g., metabolic reprogramming and extracellular matrix remodeling) modulate this core axis. Addressing these gaps will be essential to elevate the translational potential of ferroptosis research and overcome current limitations in targeted therapy. The molecular mechanisms of ferroptosis and the associated regulatory signaling pathways are depicted in **Figure 3**.

## 3 Roles of ferroptosis in the pathogenesis of renal diseases

### 3.1 Revisiting the distinctive link between ferroptosis and acute kidney disease

Ischemia–reperfusion (I/R) is a common cause of AKI, often occurring in kidney transplantation, shock, trauma, and urological and cardiovascular surgeries. It is characterized by initial reduced blood supply to tissues (ischemia) and subsequent reoxygenation via restored blood flow (reperfusion) (71). Ischemia induces cellular metabolic imbalance and reduced tissue oxygen utilization; reperfusion generates excessive free radicals, triggering inflammatory responses and ROS overproduction that exacerbate tissue damage or cause secondary injury (71). Ferroptosis, a novel iron- and ROS-dependent cell death, has attracted significant attention in I/R-related research. Recent renal I/R animal and cell models confirm its involvement in I/R-induced AKI pathogenesis. Ferroptosis inhibitors or iron chelators can notably regulate ferroptosis-related pathway protein expression in post-reperfusion renal TECs, enhance cell viability, and effectively alleviate renal I/R injury (72). Current research on ferroptosis in I/R-induced AKI mainly focuses on GSH-related amino acid metabolism, lipid metabolism, and iron metabolism. These studies clarify ferroptosis’s role in pathogenesis, illuminate renal I/R injury mechanisms, and identify potential therapeutic targets, highlighting the importance of understanding ferroptosis in developing novel renal I/R injury treatments.

#### 3.1.1 The specific role of the system Xc^−^–GSH–GPX4 axis in I/R AKI

System Xc^−^, a key cellular antioxidant system, comprises two subunits (SLC7A11, the primary activity regulator, and SLC3A2) and localizes to the phospholipid bilayer. It mediates the 1:1 exchange of glutamate and cystine; intracellular cystine is reduced to cysteine, a rate-limiting precursor for GSH biosynthesis. GSH maintains the antioxidant function of GPX4—a central ferroptosis regulator that catalyzes GSH oxidation to oxidized glutathione (GSSG) while reducing cytotoxic lipid peroxides to non-toxic alcohols (L-OH), thereby mitigating oxidative damage (33). Inhibition of System Xc^−^ blocks cystine import, disrupting GSH synthesis, impairing cellular antioxidant capacity, and inactivating GPX4, ultimately driving intracellular ROS accumulation and cell death. This System Xc^−^/GSH–GPX4 axis is thus a major ferroptosis pathway, with direct relevance to renal I/R injury. For instance, erastin, a selective System Xc^−^ inhibitor, induces ferroptosis and AKI in renal TECs (73). Wang et al. (74) further linked this axis to I/R AKI pathogenesis: renal GPX4 levels were markedly reduced in I/R-injured mice, while quercetin administration upregulated SLC7A11 and GPX4 to alleviate renal damage. At the cellular level, ATF3 knockout enhanced SLC7A11/GPX4 expression and mitigated injury, identifying ATF3 as a core ferroptosis driver that regulates the System Xc^−^/GSH–GPX4 axis in I/R AKI. Legumain—an asparaginyl endopeptidase with unclear roles in renal homeostasis—also modulates this pathway: Legumain knockout reduced I/R-induced renal ferroptosis and increased GPX4 protein (but not mRNA) levels. Mechanistically, Legumain mediates the chaperone-mediated autophagy of GPX4 via HSP90/HSC70, decreasing GPX4 abundance and promoting AKI-associated ferroptosis (75). Similarly, ALR gene knockout in an *in vitro* I/R model alleviated ferroptosis by regulating the GSH–GPX4 pathway (76). Collectively, these studies position the System Xc^−^/GSH–GPX4 axis as a critical therapeutic node in renal I/R injury: interventions targeting pathway-related genes or enhancing key protein levels boost renal antioxidant capacity, offering novel strategies to mitigate I/R-induced kidney damage.

#### 3.1.2 The distinct mechanisms of lipid peroxidation in I/R AKI

Excessive lipid peroxidation is a defining hallmark of ferroptosis, with its dysregulation directly contributing to renal I/R-induced AKI. This process relies on three key enzymes: LPCAT3, ACSL4, and ALOX. PUFAs are first converted to PUFA-CoAs by ACSL4 (a phospholipid metabolism-related enzyme) and then esterified to phospholipid-PUFAs (PLPUFAs) by LPCAT3; PLPUFAs are subsequently oxidized by ALOX or other oxidases (e.g., CYP450 and prostaglandin-endoperoxide synthase 2) to form lipid peroxides—key signaling molecules that drive ferroptosis. Modulating the expression or activity of ACSL4, LPCAT3, or ALOX directly alters cellular sensitivity to ferroptosis. In renal I/R AKI, lipid peroxidation is a pathogenic driver: lipid peroxide inhibitors ameliorate renal tissue injury and reduce ferroptosis in I/R-induced AKI rats and *in vitro* hypoxia/reoxygenation models, confirming its causal role (77). Wang et al. (78) further pinpointed ACSL4 via RNA sequencing: ACSL4 was highly expressed in renal I/R tissues, and ACSL4 knockout or ACSL inhibition significantly suppressed ferroptosis in renal TECs of I/R AKI mice, positioning ACSL4 as a critical therapeutic target. Beyond enzymes, upstream regulators also link lipid peroxidation to renal I/R ferroptosis: Zhao et al. (79) showed that the DAMP molecule HMGB1 induces ferroptosis by binding to ACSL4 in renal I/R injury. Liu et al. (80) found that prostaglandin E2 (PGE2, an arachidonic acid precursor) accumulates during renal I/R, promoting ferroptosis in renal tissues; PGE2 blockade alleviates this effect. Additionally, aryl hydrocarbon receptor (AHR) activation during renal tubular epithelial cell reoxygenation induces ROS production, lipid peroxidation, and ferroptosis via AHR-mediated CYP enzyme overexpression, identifying AHR as another potential therapeutic target (81). Collectively, these findings establish lipid peroxidation and its regulatory network (enzymes, HMGB1, PGE2, and AHR) as central to renal I/R AKI pathogenesis, offering actionable targets to mitigate ferroptosis-driven kidney damage.

#### 3.1.3 Profound links between dysregulation of iron metabolism and I/R AKI

Iron metabolic homeostasis—governed by absorption, utilization, and recycling—is critical for suppressing ferroptosis: disruption of this balance elevates free iron, which accelerates ROS accumulation via the Fenton reaction and renders cells more susceptible to ferroptosis (45, 82). Clinically, reduced iron-binding protein levels during surgery indirectly reflect impaired catalytic iron regulation during extracorporeal circulation, contributing to renal injury (83), underscoring the relevance of iron homeostasis to renal I/R pathophysiology. In renal I/R models, iron chelators prevent tubular cell death (84), confirming iron ions as key mediators of I/R-induced AKI-associated ferroptosis; targeting free iron via chelation thus mitigates I/R-driven renal damage. Beyond basic iron handling, specific regulators link iron metabolism to renal ferroptosis: Pannexin1 (Panx1), a known promoter of renal injury-associated apoptosis, was identified by Su et al. (85) to additionally regulate ferroptosis in I/R AKI. Other critical players include NCOA4 (a ferritin degradation receptor that maintains iron homeostasis via ferritinophagy; its inhibition eliminates labile iron and ROS to block ferroptosis), HO-1 (a heme catabolism enzyme with ferroptosis-suppressive protective effects), and ELAVL1 (a ferritinophagy regulator that promotes ferroptosis). Under hypoxia/reoxygenation conditions, mimicking I/R-ELAVL1 inhibition or knockdown reverses CIRBP-enhanced ferritinophagy and ferroptosis in renal TECs, implicating CIRBP–ELAVL1 crosstalk in driving ferritinophagy-mediated ferroptosis in renal I/R. Iron metabolism, together with the System Xc^−^/GSH–GPX4 axis and lipid peroxidation pathways, constitutes the classical ferroptosis machinery. Upregulating GSH-dependent antioxidant defenses, suppressing lipid peroxidation, and preserving iron homeostasis collectively reduce ferroptosis to alleviate I/R AKI. Extensive studies of these pathways have uncovered upstream regulators (e.g., Legumain, ALR, and Panx1), deepening the mechanistic understanding of renal I/R ferroptosis and expanding therapeutic opportunities for targeting ferroptosis in I/R AKI.

#### 3.1.4 Roles of epigenetically mediated ferroptosis in I/R AKI

Ubiquitination regulates signaling via mediating protein turnover, with deubiquitination reversing this process by detaching ubiquitin from substrates; both processes govern ferroptosis in renal I/R injury. Pan et al. (86) showed that ubiquitin-specific protease 14 (USP14) is upregulated in hypoxia/reoxygenation (H/R)-exposed renal TECs and I/R mouse renal tissues; USP14 inhibition (via siRNA or small-molecule inhibitor IU1) alleviated renal I/R injury by reducing COX2/ACSL4/NOX1 upregulation and preventing GPX4/FTH1 downregulation. Ubiquitin-specific protease 7 (USP7) is also upregulated in H/R-treated cells: USP7 intervention enhanced renal tubular epithelial cell proliferation, increased GPX4/SLC7A11 levels, and reduced iron accumulation/oxidative stress. Mechanistically, USP7 inhibition suppressed ferroptosis by decreasing TANK-binding kinase 1 (TBK1) ubiquitination and promoting DNMT1-mediated FMR1 methylation. In I/R-induced AKI, miRNAs act as central ferroptosis regulators via targeting key genes. Tao et al. (87) demonstrated that miR-3587 directly targets the ferroptosis-related gene HMOX1 in H/R-treated renal TECs; miR-3587 inhibition upregulated HO-1 (the HMOX1-encoded protein), protecting renal tissues from I/R-induced ferroptosis. Conversely, Ding et al. (88) found that H/R conditions upregulated miR-182-5p and miR-378a-3p in renal TECs, leading to GPX4 downregulation and subsequent ferroptosis. Lysine-specific demethylase 1 (LSD1), a kidney disease-associated epigenetic regulator, promotes oxidative stress and ferroptosis in renal I/R injury. LSD1 inhibition blocked I/R-induced ferroptosis and oxidative stress by downregulating the TLR4/NOX4 pathway, identifying LSD1 as a potential therapeutic target. Inositol-requiring enzyme 1 (IRE1)—a proximal endoplasmic reticulum (ER) stress sensor—activates the c-Jun N-terminal kinase (JNK) pathway during ER stress. IRE1/JNK inhibition reduced blood urea nitrogen (BUN), creatinine levels, and renal tissue damage in I/R mice while normalizing ferroptosis biomarkers (e.g., 4-HNE and GPX4). Cardiopulmonary resuscitation (CPR) is a major cause of renal I/R. In a CPR-induced I/R pig model, the aldehyde dehydrogenase 2 (ALDH2) activator ALDA-1 alleviated renal injury: compared to the CPR group, ALDA-1 treatment reduced renal iron deposition, MDA, 4-HNE, and ACSL4 levels, while increasing GSH and GPX4 expression (89).

#### 3.1.5 Targeting ferroptosis in I/R AKI

Natural products or herbal medicines hold potential for the clinical treatment of renal I/R injury. Pachymic acid (PA), a triterpenoid compound isolated from *Poria cocos*, has been shown to ameliorate renal injury in a mouse model of renal I/R, potentially by inhibiting renal ferroptosis through the upregulation of the NRF2/HO-1 axis (90). Paeoniflorin significantly alleviates I/R-induced AKI by upregulating SLC7A11 to inhibit ferroptosis (91). Chrysophanol, a traditional Chinese medicine used clinically for kidney diseases, has been demonstrated to mitigate I/R-induced ferroptosis by modulating GPX4 and SLC7A11 (84). These herbal medicines, which have previously been shown to exert protective effects against renal injury, possess anti-inflammatory and antioxidant properties. Some clinically used drugs have also been shown to protect the kidneys from I/R injury. Entacapone, a specific inhibitor of catechol-*O*-methyltransferase (COMT), has long been used as an adjunctive treatment for Parkinson’s disease. Recent studies have found that entacapone reverses ferroptosis and alleviates AKI by inhibiting lipid peroxidation and iron accumulation, with its mechanism involving the regulation of SLC7A11 to enhance antioxidant capacity (92). Dimethyl fumarate (DMF), a small-molecule drug used to treat multiple sclerosis and psoriasis, prevents ferroptosis and improves AKI by acting on NRF2 and exerting anti-peroxidative effects (93). Melatonin (MT), a pineal hormone and potent antioxidant, prevents ferroptosis in renal TECs via the NRF2/SLC7A11 axis (94). Vitamin K1 may improve renal I/R injury through its antioxidant effects (94). Dexmedetomidine, an α2-adrenergic receptor (α2-AR) agonist, may protect against I/R-induced renal injury by suppressing the upregulation of ACSL4 (95). Silibinin targets FTH1, disrupts the NCOA4–FTH1 interaction, reduces ferroptosis-mediated cell death, and alleviates renal dysfunction, pathological damage, and inflammation in ischemia–reperfusion injury (IRI) AKI mice, effectively preventing ischemia–reperfusion-induced renal injury (96).

Despite substantial progress in delineating ferroptosis-related mechanisms and potential therapeutics in I/R-induced AKI, critical knowledge gaps and translational bottlenecks persist that limit the clinical utility of these findings and hinder a holistic understanding of disease pathogenesis. First, the crosstalk network between distinct ferroptosis-regulating pathways (System Xc^−^–GSH–GPX4 axis, lipid peroxidation, iron metabolism, and epigenetic regulation) remains poorly defined—whether these pathways act in a hierarchical, synergistic, or compensatory manner in different phases of I/R injury (ischemia *vs*. reperfusion) or across renal cell subsets (e.g., tubular epithelial cells, glomerular cells, and immune cells) has not been systematically dissected. Current studies have largely focused on individual pathways in isolation, failing to capture the complexity of *in vivo* ferroptosis regulation. Second, cell type and microenvironmental specificity are insufficiently addressed: most research centers on renal tubular epithelial cells, while the role of ferroptosis in other renal resident cells (e.g., podocytes and mesangial cells) or infiltrating immune cells (e.g., macrophages and neutrophils) during I/R injury is largely unexplored. Additionally, how factors such as renal inflammation, hypoxia gradient, or metabolic reprogramming in the I/R microenvironment modulate ferroptosis susceptibility across cell types remains elusive. Third, translational gaps between preclinical models and clinical practice are prominent: most therapeutic candidates (e.g., natural products and repurposed drugs) have only been validated in rodent models, with limited data on human renal tissue responses, optimal dosing windows, or potential drug–drug interactions in clinical settings (e.g., in patients undergoing kidney transplantation or cardiovascular surgery). Moreover, no clinically validated biomarkers exist to stratify AKI patients based on ferroptosis activity, hindering the development of precision therapies. Fourth, long-term pathogenic implications are overlooked: current research focuses primarily on acute injury alleviation, while the role of ferroptosis in AKI-to-chronic kidney disease (CKD) progression—e.g., whether unresolved ferroptosis drives renal fibrosis or tubular atrophy—has not been rigorously investigated. Finally, epigenetic regulatory networks are incompletely characterized: while individual ubiquitinases, miRNAs, or demethylases are implicated, the integrated epigenetic landscape governing ferroptosis in I/R AKI (e.g., crosstalk between ubiquitination and miRNA-mediated silencing, or epigenetic memory of ferroptosis susceptibility) remains unclear. Addressing these gaps requires multi-dimensional studies integrating single-cell omics, spatial transcriptomics, and clinical cohort analyses to resolve pathway crosstalk, define cell type-specific roles, and bridge preclinical and clinical translation, ultimately advancing ferroptosis-targeted strategies from bench to bedside in AKI treatment.

### 3.2 Ferroptosis: a driver of AKI progression to CKD

AKI—characterized by rapid renal function decline, with pathogenesis involving I/R, nephrotoxicity, inflammation, and immune injury (97)—is an independent risk factor for CKD. Maladaptive repair post-AKI [driven by inflammation, hypoxia, interstitial fibrosis, and tubular injury (97)] promotes AKI-to-CKD transition, and ferroptosis is tightly linked to this process, highlighting its therapeutic potential.

#### 3.2.1 Ferroptosis-mediated inflammation

Chronic inflammation is a key driver of AKI-to-CKD progression (97). While PUFAs metabolized by cyclooxygenase (COX)/LOX generate inflammatory mediators, prostaglandin-endoperoxide synthase 2 (PTGS2/COX2)—a ferroptosis biomarker—does not always correlate with ferroptosis under inflammation (98). Ferroptosis releases DAMPs (e.g., HMGB1) that activate innate immunity via pattern recognition receptors. Neutralizing HMGB1 reduces ferroptosis-induced macrophage inflammation; Zhao et al. (99) further showed HMGB1’s dual roles: mediating AKI and delaying AKI-to-CKD transition by enhancing tubular sensitivity to oxidative stress. Tao et al. (77) found that dexmedetomidine inhibits ACSL4 via α2-ARs to alleviate ferroptosis-related I/R and inflammation. Ferroptosis also amplifies inflammation: inhibiting tubular ferroptosis reduces macrophage recruitment by decreasing monocyte chemoattractant protein 1 (MCP-1) (100) and indirectly promotes neutrophil aggregation via macrophages. Ferroptosis inhibitors (e.g., irisin) exert anti-inflammatory effects by upregulating GPX4 (101), confirming ferroptosis as both an inflammatory trigger and amplifier.

#### 3.2.2 Ferroptosis-mediated hypoxia

**Figure 4:**
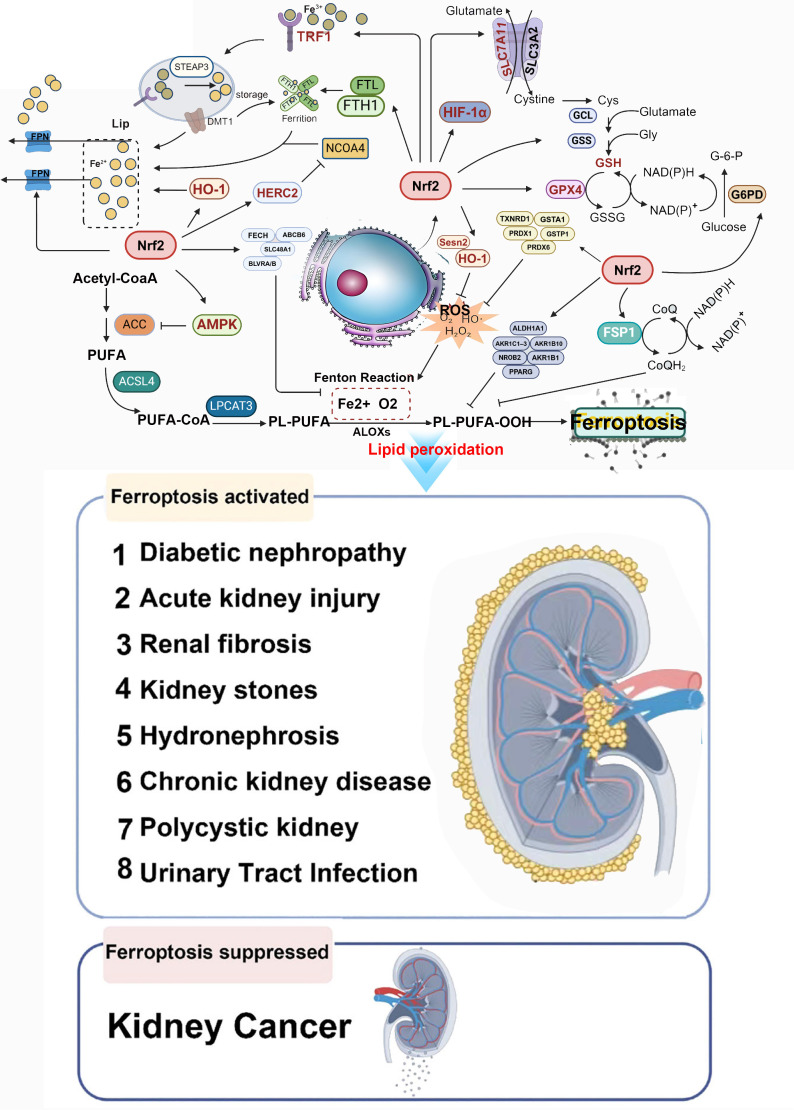
Nrf2-mediated ferroptosis in the pathogenesis of acute kidney injury (AKI) to chronic kidney disease (CKD) transition. The three core pathways regulating ferroptosis—iron metabolism, antioxidant defense, and lipid metabolism—are subject to transcriptional regulation by Nrf2, positioning Nrf2 as a central modulator of ferroptosis in the progression from AKI to CKD. Key genes involved in these processes are directly or indirectly controlled by Nrf2. TFR1, transferrin receptor 1; FPN (SLC40A1), ferroportin; STEAP3, six-transmembrane epithelial antigen of the prostate 3; DMT1 (SLC11A2), divalent metal transporter 1; FTL, ferritin light chain; FTH1, ferritin heavy chain; NCOA4, nuclear receptor coactivator 4; HERC2, HECT and RLD domain-containing E3 ubiquitin protein ligase 2; Nrf2, nuclear factor erythroid 2-related factor 2; FECH, ferrochelatase; ABCB6, ATP-binding cassette subfamily B member 6; SLC48A1, solute carrier family 48 member 1; BVRA/B, biliverdin reductase A/B; HO-1, heme oxygenase-1; Sesn2, Sestrin2; TXNRD1, thioredoxin reductase 1; GSTA1, glutathione *S*-transferase A1; PRDX1, peroxiredoxin 1; PRDX6, peroxiredoxin 6; GSTP1, glutathione *S*-transferase P1; ALDH1A1, aldehyde dehydrogenase 1 family member A1; AKR1C1, aldo-keto reductase 1C1; AKR1B1, aldo-keto reductase family 1 member B1; AKR1B10, aldo-keto reductase family 1 member B10; NR0B2, nuclear receptor subfamily 0 group B member 2; PPARG, peroxisome proliferator-activated receptor γ; GPX4, glutathione peroxidase 4; GSH, glutathione; GSSG, oxidized glutathione; GCL, glutamate–cysteine ligase; GSS, glutathione synthetase; SLC7A11, solute carrier family 7 member 11; SLC3A2, solute carrier family 3 member 2; hypoxia-inducible factor-1α (HIF-1α); AMPK, AMP-activated protein kinase; ACC, acetyl-CoA carboxylase; PUFA, polyunsaturated fatty acid; ACSL4, acyl-CoA synthetase long-chain family member 4; LPCAT3, lysophosphatidylcholine acyltransferase 3; ALOX, arachidonate lipoxygenase; CoQ10, coenzyme Q10; FSP1, ferroptosis suppressor protein 1.

Hypoxia—caused by post-AKI capillary rarefaction and endothelial damage—induces tubulointerstitial fibrosis and AKI-to-CKD transition. Transcription factors regulate ferroptosis under hypoxia. 1) Nrf2: A key antioxidant regulator; melatonin activates Nrf2 to inhibit oxidative stress/ferroptosis in hypoxic/reoxygenated tubular cells, preventing AKI (94, 102). 2) Hypoxia-inducible factor (HIF): Stabilized under hypoxia [via HIF-α/β subunits (102)]; Li et al. (103) showed that roxadustat (HIF prolyl hydroxylase inhibitor) stabilizes HIF-1α/Nrf2, reducing ferroptosis, inflammation, and fibrosis in folic acid-induced renal injury. HIF-1α downregulation increases ACSL4 to promote ferroptosis, and Nrf2 mediates HIF-1α activation to alleviate ischemic AKI (102). 3) REST: Upregulated in AKI (104), suppresses glutamate-cystine ligase modifier subunit (GCLM) transcription, reducing GSH/GPX4 to promote lipid peroxidation/ferroptosis; tubular REST knockout ameliorates AKI-to-CKD progression (104). Additional mechanisms include “synchronized ferroptosis” in proximal tubular cells [propagating injury (105)] and hypoxia-induced apical small extracellular vesicles [triggering tubular ferroptosis, inhibited by ferroptosis inhibitors/RNase A (106)]. These studies reveal the pivotal roles of transcription factors such as Nrf2, HIF, and REST in regulating ferroptosis during the transition from AKI to CKD, providing directions for the development of novel therapeutic targets. The detailed mechanism of Nrf2-mediated ferroptosis in the pathogenesis of AKI-to-CKD transition is presented in **Figure 4**.

#### 3.2.3 Ferroptosis-mediated renal interstitial fibrosis

Renal interstitial fibrosis—an endpoint of CKD—is promoted by ferroptosis in AKI models (100, 103). The core mechanisms included the following. 1) TGF-β/Smad–HDAC3 axis: HDAC3 [regulated by TGF-β/Smad (107)] promotes fibrosis; HDAC3 inhibitors restore anti-fibrotic Klotho. Zhang et al. (108) found elevated HDAC3 (with downregulated GPX4) in aristolochic/folic acid-induced AKI-to-CKD transition; tubular HDAC3 knockout upregulates GPX4 and improves fibrosis. 2) Epithelial-to-mesenchymal transition (EMT): EMT reduces ferritin heavy chain (FTH), releasing free iron to trigger ferroptosis; exogenous FTH reduction exacerbates fibrosis (100). 3) Fatty acid oxidation (FAO) impairment: post-AKI FAO loss (109) contributes to fibrosis (110); ferroptosis targeting alleviates fibrosis and delays AKI-to-CKD transition. Targeting ferroptosis effectively reduces the extent of renal interstitial fibrosis, improves renal function, and delays the progression from AKI to CKD. Beyond their individual pathogenic roles, ferroptosis, inflammation, hypoxia, and renal interstitial fibrosis form a synergistic and self-amplifying integrated pathogenic network that drives the irreversible AKI-to-CKD transition. At the core of this network, ferroptosis acts as a central initiating and amplifying hub, while inflammation and hypoxia function as reciprocal triggers and amplifiers, ultimately converging to promote fibrosis, the terminal pathological feature of CKD.

Regarding the integrated pathogenic network linking ferroptosis with inflammation, hypoxia, and fibrosis, in this network, the initial AKI-induced tubular injury (e.g., from I/R or nephrotoxicity) directly triggers ferroptosis via mitochondrial dysfunction and iron overload. Ferroptotic tubular cells release DAMPs (e.g., HMGB1) and lipid peroxidation products (e.g., 4-HNE), which activate pattern recognition receptors on macrophages and neutrophils, inducing the secretion of pro-inflammatory cytokines (e.g., IL-33 and MCP-1). These cytokines not only amplify local inflammation but also further disrupt the System Xc^−^–GSH–GPX4 axis and upregulate ACSL4 in neighboring tubular cells, creating a “ferroptosis-inflammatory cascade” that expands tubular injury. Concurrently, ferroptosis-mediated tubular and endothelial cell damage reduces renal microvascular density (capillary rarefaction) and impairs oxygen delivery, establishing a hypoxic microenvironment. Hypoxia then stabilizes HIF-1α and upregulates REST, which suppresses GCLM/GPX4 expression and enhances ACSL4-dependent lipid peroxidation—directly amplifying ferroptosis—while also promoting the release of hypoxic extracellular vesicles (EVs) that propagate tubular ferroptosis in a paracrine manner. This ferroptosis–inflammation–hypoxia loop ultimately converges on fibrosis progression: pro-inflammatory cytokines (e.g., from activated macrophages) and hypoxic signals synergistically activate the TGF-β/Smad–HDAC3 pathway, downregulating anti-fibrotic Klotho and upregulating pro-fibrotic factors (e.g., collagen I/III). Additionally, ferroptosis-induced tubular injury promotes EMT via FTH downregulation, releasing free iron that further amplifies ferroptosis and converts epithelial cells to pro-fibrotic mesenchymal cells. Meanwhile, hypoxia-induced FAO impairment in tubular cells exacerbates lipid accumulation and ferroptosis, while fibrosis itself creates a pathological microenvironment—compressing renal microvessels to sustain hypoxia and trapping inflammatory cells to perpetuate inflammation—thus closing the loop and reinforcing the ferroptosis–inflammatory–hypoxic–fibrotic cascade.

Notably, key transcription factors (e.g., Nrf2 and HIF-1α) act as critical “regulatory checkpoints” within this network: Nrf2 inhibits ferroptosis and inflammation by upregulating GPX4 and antioxidant enzymes, while HIF-1α exerts a context-dependent dual role, acutely mitigating ferroptosis via Nrf2 activation but promoting fibrosis and sustained ferroptosis when chronically stabilized. Disruption of this integrated network (e.g., via ferroptosis inhibitors targeting ACSL4/GPX4, anti-inflammatory agents blocking HMGB1/IL-33, or HIF modulators balancing hypoxia adaptation) can break the pathogenic cycle, highlighting the therapeutic potential of targeting the network’s central nodes rather than individual pathways. This integrated network explains the irreversibility of AKI-to-CKD transition: once the reciprocal amplification between ferroptosis, inflammation, hypoxia, and fibrosis is initiated, each component reinforces the others, driving progressive and persistent renal damage. Elucidating this interconnected circuitry provides a more comprehensive framework for understanding AKI-to-CKD progression and identifies multi-targeted strategies that disrupt the network at critical junctions.

#### 3.2.4 Ferroptosis-mediated renal tubular epithelial cell injury

Tubular injury—hallmark of AKI—drives AKI-to-CKD progression. Proximal tubules (high energy demand and mitochondria-rich) rely on FAO for ATP (110); post-AKI mitochondrial dysfunction increases mitochondrial ROS to trigger ferroptosis (111). Damaged tubules promote inflammation/fibrosis via secretory phenotype shifts or G2/M phase arrest [paracrine effects (112)]. Targeting tubular ferroptosis is protective. 1) XJB-5-131 (mitochondrial nitroxide) inhibits lipid peroxidation to prevent ferroptosis, reducing inflammatory infiltration and promoting tubular repair (113). 2) Gene targeting: Proximal tubule FTH knockout reduces AKI mouse survival (94); tubular ACSL4 knockout alleviates inflammation, lipid peroxidation, and ferroptosis-mediated AKI (96).

#### 3.2.5 Ferroptosis modulators in AKI-to-CKD intervention

Ferroptosis modulators (**Table 1**) target ferroptosis components to prevent AKI-to-CKD transition. 1) Anti-inflammatory: Ferrostatin-1 inhibits interleukin-33 (IL-33) processing/release, reducing macrophage infiltration (114). 2) Anti-ferroptotic under hypoxia: Triptolide activates Nrf2/GPX4 to treat cisplatin-induced AKI (115). 3) Anti-fibrotic: Liproxstatin-1 (Lip-1) reduces pro-fibrotic factor secretion from ferroptotic tubules to inhibit fibroblast activation (116); roxadustat/irisin also suppresses fibrosis (101, 103). 4) Most modulators remain experimental; further research is needed for clinical translation. Additionally, both roxadustat and irisin have been shown to inhibit the progression of fibrosis (101, 103). Currently, research on these drugs remains limited to experimental stages, and further exploration is needed to establish a foundation for clinical applications.

**Table 1:** Ferroptosis modulators.

Class	Compound	Target	Mechanism of action
Inhibitors	Ferrostatin-l	Lipid peroxidation	Inhibits lipid peroxidation
	Liproxstatin-1	Lipid peroxidation	Inhibits lipid peroxidation
	XJB-5-131	Lipid peroxidation	Inhibits lipid peroxidation
	Irisin	CPX4	Upregulates GPX4 expression
	Melatonin	System Xc^−^.GPX4	Upregulates System Xc^−^ and GPX4 expression
	Celastrol	CPX4	Upregulates GPX4 expression
	Dexmedetomidine	ACSIA	Inhibits ACSL4 expression
Inducers	Erastin and analogs	System Xc^−^	Inhibits cystine uptake, leading to GSH depletion
	Sulfasalazine	System Xc^−^	Inhibits cystine uptake, leading to GSH depletion
	Sorafenib	System Xc^−^	Inhibits cystine uptake, leading to GSH depletion
	RSL3	CPX4	Inhibits cystine uptake, leading to GSH depletion
	Brequinar	DHODH	Inhibits DHODH
	iFSP1	FSP1	Inhibits FSP1

GPX4, glutathione peroxidase 4; System Xc^−^, cystine/glutamate antiporter; ACSL4, acyl-CoA synthetase long-chain family member 4; GSH, glutathione; DHODH, dihydroorotate dehydrogenase; FSP1, ferroptosis suppressor protein 1; RSL3, glutathione peroxidase 4 (GPX4) inhibitor.

While cumulative evidence establishes ferroptosis as a pivotal mediator of AKI-to-CKD transition and identifies candidate regulatory pathways and modulators, several critical research gaps remain that require systematic exploration to advance the field’s depth and translational potential; these gaps also highlight unmet needs for precise intervention in chronic kidney disease progression.

1. Mechanistic gaps in pathway crosstalk and causality: Current studies have primarily focused on individual pathways (e.g., Nrf2-mediated antioxidant defense and TGF-β/Smad–HDAC3-driven fibrosis) but lack insights into the core molecular hubs integrating these networks. For instance, how Nrf2, HIF-1α, and REST coordinate to regulate ferroptosis across different stages of AKI-to-CKD transition (e.g., acute injury *vs*. established fibrosis) remains undefined—whether they act sequentially, synergistically, or antagonistically depends on contextual cues that have not been elucidated. Additionally, the causal relationship between EMT and ferroptosis is poorly resolved: existing data only confirm mutual association, but it is unclear whether EMT-induced FTH downregulation is a primary trigger of ferroptosis or a secondary response to ferroptotic tubular injury. Furthermore, the interplay between ferroptosis and other programmed cell death pathways (e.g., pyroptosis and necroptosis) in driving renal fibrosis is fragmented; whether these cell death modalities crosstalk to amplify injury or compensate for each other in the transition phase lacks systematic investigation.
2. Cell type- and etiology-specific regulatory blank spots: Most research centers on renal TECs, but the role of non-parenchymal renal cells in ferroptosis-mediated AKI-to-CKD transition is largely understudied. For example, whether renal interstitial fibroblasts, endothelial cells, or immune cell subsets (e.g., M1/M2 macrophages and neutrophils) undergo ferroptosis themselves—and how this cell type-specific ferroptosis contributes to inflammation or fibrosis—has not been rigorously characterized. Moreover, ferroptosis mechanisms are predominantly investigated in I/R or nephrotoxic AKI models, with limited data on sepsis-induced, metabolic (e.g., diabetic), or autoimmune AKI subtypes. It remains unknown whether the core drivers of ferroptosis (e.g., ACSL4 and GPX4) vary across these etiologies, which hinders the development of etiology-tailored therapies.
3. Limitations in biomarker development and clinical translation: A major translational gap lies in the lack of stage-specific, ferroptosis-related biomarkers for predicting AKI-to-CKD progression. Current markers (e.g., GPX4 and 4-HNE) are non-specific to the transition phase, failing to distinguish acute ferroptosis (reversible) from chronic ferroptosis (fibrosis-promoting) in clinical samples. Additionally, most ferroptosis modulators (e.g., ferrostatin-1 and triptolide) are evaluated in preclinical models of early AKI, but their efficacy in patients with established CKD (e.g., with tubulointerstitial fibrosis) is untested—whether inhibiting ferroptosis can reverse or halt existing fibrosis, or only delay progression from AKI, remains unclear. Furthermore, renal-specific drug delivery systems for ferroptosis modulators are lacking; systemic administration carries risks of off-target effects (e.g., promoting tumorigenesis via global ferroptosis inhibition), which have not been addressed in long-term safety assessments.
4. Underexplored epigenetic and long-term regulatory mechanisms: Epigenetic regulation of ferroptosis in AKI-to-CKD transition is largely limited to individual molecules (e.g., miRNAs targeting GPX4), with no comprehensive understanding of epigenetic landscapes (e.g., long non-coding RNAs, histone modifications, and DNA methylation) that persistently modulate ferroptosis-related genes during the chronic phase. Additionally, the impact of host factors (e.g., age, genetics, and comorbidities like hypertension or diabetes) on ferroptosis sensitivity in AKI-to-CKD transition is understudied; whether genetic polymorphisms in ferroptosis regulators (e.g., SLC7A11 and ACSL4) contribute to inter-individual differences in progression risk remains unknown. Finally, the role of ferroptosis in renal fibrosis resolution is entirely unexplored: it is unclear whether activating or inhibiting ferroptosis could promote fibrosis regression in advanced CKD, a critical question for reversing established disease. These gaps underscore the need for future research to move beyond descriptive associations, toward mechanistic dissection of pathway integration, cell type-specific functions, and clinical validation of biomarkers and targeted therapies; only by addressing these areas can ferroptosis-targeted strategies fulfill their potential for improving outcomes in AKI and CKD patients.

### 3.3 The role of ferroptosis in the pathogenesis of DN

Diabetes prevalence is rising globally, with diabetic nephropathy (DN)—affecting 20%–50% of diabetics—being the leading cause of ESRD (117). High glucose drives renal cell damage (podocytes, TECs, etc.) (118), and ferroptosis (a non-apoptotic cell death modality) is a key mediator of DN progression. Hyperglycemia-induced ROS overproduction, iron overload, and antioxidant system inactivation synergistically trigger ferroptosis (119, 120).

#### 3.3.1 System Xc^−^–GSH–GPX4 axis imbalance

GPX4, the central suppressor of ferroptosis, detoxifies lipid peroxides (PLOOH) with GSH as a cofactor in the pathogenesis of DN (121–124). GSH depletion or GPX4 inactivation induces lipid peroxidation (e.g., MDA accumulation) and ferroptosis (125). In DN, 1) renal biopsy samples show downregulated SLC7A11 (System Xc^−^ subunit) and GPX4, with reduced GSH and elevated lipid peroxides under high glucose (HG) (124, 126). 2) High-fructose diets induce podocyte ferroptosis via SLC7A11/GPX4 downregulation (127), while GPX4 deficiency causes tubular cell death and proteinuria (128).

#### 3.3.2 Iron overload under high glucose promotes ROS generation and triggers ferroptosis

HG disrupts iron homeostasis: upregulated TFR1 (iron uptake) and downregulated FTH1 (iron storage) lead to LIP expansion (129, 130). Excess Fe^2+^ triggers Fenton reactions, generating ROS and lipid peroxides. Key evidence: 1) Renal iron concentration is fourfold higher in diabetic mice (131). 2) Iron deposition occurs in TECs [human DN (132)] and podocytes [HG-stimulated (133)], initiating ferroptosis (134).

#### 3.3.3 Lipid peroxidation under high-glucose conditions triggers ferroptosis

PUFAs are susceptible to ROS-mediated peroxidation, forming PLOOH (135). In the absence of GPX4, Fe^2+^ amplifies peroxyl chain reactions (136), and PLOOH accumulation directly induces ferroptosis (137). DN mouse kidneys show increased lipid peroxides, which disrupt membrane permeability and exacerbate renal injury (138). Under high-glucose conditions, the accumulation of lipid hydroperoxides in cell membrane structures increases membrane permeability, thereby inducing ferroptosis and exacerbating renal damage in DN.

#### 3.3.4 Mitochondrial dysfunction under high-glucose conditions exacerbates ferroptosis

Mitochondria are major ROS sources in ferroptosis. HG-induced iron overload generates hydroxyl radicals, causing mitochondrial membrane potential (MMP) depolarization, mtDNA damage, and dysfunction (139–142). Mitochondrial iron dyshomeostasis further amplifies ferroptosis (140–142). In DN renal tissues, mitochondrial accumulation due to dysfunction can be clearly observed, indicating that mitochondrial damage plays a significant role in ferroptosis during DN (143).

#### 3.3.5 Antioxidant proteins as potential therapeutic targets for ferroptosis in DN

Ferroptosis in DN is governed by a core network of antioxidant proteins, with Nrf2 as the master transcriptional regulator. Nrf2 orchestrates redox homeostasis by inducing genes encoding GPX4, FTH1, NQO1, and HO-1, thereby stabilizing GSH pools, suppressing lipid peroxidation, and modulating iron and mitochondrial metabolism (37, 144, 145). Its expression is suppressed in high glucose-exposed podocytes, and Nrf2 deficiency heightens renal oxidative stress and ferroptotic susceptibility (146, 147). Conversely, Nrf2 activation—by pharmacological agents or genetic means—upregulates GSH, GPX4, and FTH1, mitigating podocyte injury and DN progression (120, 148, 149). Dexmedetomidine, empagliflozin, and HO-1 induction further protect against ferroptosis via the Nrf2/GPX4 or AMPK/Nrf2 axis (150–153). The sirtuin family fine-tunes this response. Sirt1, downregulated in DN podocytes (154), enhances Nrf2 activity by deacetylating and inhibiting p53 (155, 156); Sirt1 activators (e.g., SRT2104) or Astragaloside IV restore GPX4 and suppress ferroptosis (157). Mitochondrial Sirt3, also diminished in DN (158), preserves redox balance through SOD2/CAT activation via FoxO3a (159), sustains AMPK/mTOR signaling to limit autophagy-driven ferroptosis (160), and cooperates with UCP2/PGC1α to maintain mitochondrial integrity (161). *N*-Acetylcysteine rescues Sirt3–SOD2/GPX4 signaling, attenuating renal ferroptosis (158). Ferritin, particularly FTH1, sequesters labile iron via ferroxidase activity, directly antagonizing iron-dependent lipid peroxidation (28, 30). Podocyte-specific FTH1 overexpression confers protection (162), while its loss promotes ferroptosis; Nrf2 transcriptionally regulates FTH1 (163, 164), and interventions like resveratrol or HO-1 activation reinforce the Nrf2/FTH1/GPX4 axis to suppress ferroptosis (165, 166). At the effector level, GPX4 neutralizes phospholipid hydroperoxides and is indispensable for membrane integrity (167). Its downregulation in DN kidneys correlates with injury (121), whereas its restoration—by vitexin (123), ginkgolide B (via inhibition of GPX4 ubiquitination) (121), platycodin D (125), or Buyang Huanwu Decoction (168)—robustly inhibits ferroptosis. Functionally, GPX4 integrates inputs from Nrf2, Sirt1, Sirt3, and FTH1 into a unified cytoprotective node. Thus, GPX4, as a downstream target of Sirt1, Sirt3, Nrf2, and FTH1 proteins, interacts with these molecules to form a network of potential therapeutic targets for ferroptosis in DN.

#### 3.3.6 Natural compounds and TCM intervention in intervening ferroptosis in DN

Numerous studies have reported that active components of traditional Chinese medicine (TCM) (121, 123, 125, 147, 169–179) and TCM compound formulas (83, 120, 180–186) can ameliorate renal injury and ferroptosis in DN by activating antioxidant proteins. For instance, Jin et al. (149) demonstrated that 7-hydroxycoumarin inhibits oxidative stress and ferroptosis in the kidneys of db/db mice by activating the Nrf2/HO-1 signaling pathway, thereby exerting a protective effect against DN. Similarly, Buyang Huanwu Decoction reduces DN murine renal histopathology by upregulating SLC7A11 and GPX4 to restrain ferroptosis (180); Shenqi Dihuang Decoction attenuates high glucose-induced ferroptosis in HK-2 cells by activating the Nrf2/HO-1/GPX4 axis, lowering Fe^2+^ and ROS, and mitigating peroxidative damage (181). Notably, TCM compound formulas remain understudied in this context, representing a critical avenue for future DN therapeutic development.

While current studies have delineated the core roles of ferroptosis (via the System Xc^−^–GSH–GPX4 axis, iron overload, lipid peroxidation, and mitochondrial dysfunction) and identified antioxidant networks (Nrf2/Sirtuins/FTH1) as therapeutic targets in DN, several critical research gaps persist that hinder a comprehensive understanding of ferroptosis-driven DN progression and translational application; addressing these gaps will significantly enhance the clinical relevance and depth of this field. 1) Mechanistic gaps in pathway integration and novel regulatory nodes: Existing research focuses on individual ferroptosis-related pathways but lacks insights into the hierarchical integration and reciprocal regulation of these networks in DN. For instance, how Nrf2, Sirt1, and Sirt3 synergistically or antagonistically modulate GPX4/FTH1 expression under chronic high-glucose conditions remains undefined; whether they form a core regulatory module or act via independent downstream cascades has not been systematically dissected. Additionally, the role of non-classical ferroptosis regulators (e.g., FSP1, DHODH, and GCH1/BH4) in DN is largely unexplored; it is unknown whether these radical-trapping antioxidant pathways compensate for GPX4 deficiency in diabetic kidneys or if their dysfunction contributes to ferroptosis susceptibility. Furthermore, the crosstalk between ferroptosis and other DN-related pathological processes [e.g., advanced glycation end product (AGE)-RAGE signaling and renin–angiotensin system (RAS) activation] is fragmented; whether AGEs directly induce ferroptosis via iron overload or lipid peroxidation, or indirectly by suppressing Nrf2/Sirtuin pathways, requires definitive validation. 2) Cell type- and subtype-specific regulatory blank spots: Most studies have centered on podocytes and TECs, but the role of renal non-parenchymal cells (e.g., mesangial cells, renal endothelial cells, and inflammatory cells) in ferroptosis-driven DN is understudied. For example, whether mesangial cell ferroptosis contributes to glomerular sclerosis or if endothelial ferroptosis exacerbates renal microvascular dysfunction in DN has not been rigorously characterized. Moreover, DN exhibits significant clinical heterogeneity (e.g., type 1 *vs*. type 2 DN, albuminuric *vs*. non-albuminuric subtypes), but ferroptosis mechanisms across these subtypes remain unexplored. It is unclear whether the core drivers of ferroptosis (e.g., iron overload *vs*. antioxidant exhaustion) vary by DN subtype, which limits the development of personalized therapies. Additionally, the contribution of ferroptosis in different renal compartments (glomerulus *vs*. tubulointerstitium) to DN progression—whether glomerular ferroptosis predominates in early DN and tubulointerstitial ferroptosis drives late-stage fibrosis—lacks spatial-temporal characterization. 3) Limitations in clinical biomarkers and translational research: A major translational gap is the absence of DN-specific ferroptosis biomarkers with clinical utility. Current markers (e.g., GPX4, lipid peroxides, and serum iron) lack specificity for DN, as they are also altered in other kidney diseases or metabolic disorders. Validating non-invasive biomarkers (e.g., urine exosomal ferroptosis-related proteins and circulating lipid peroxide metabolites) that reflect renal ferroptosis activity in DN patients is urgently needed for early diagnosis and therapeutic monitoring. Furthermore, most preclinical studies have used short-term high-glucose or db/db mouse models, which fail to recapitulate the chronic, progressive nature of human DN (e.g., long-term fibrosis and comorbidity-associated complications). The efficacy of ferroptosis inhibitors (e.g., GPX4 activators) or natural compounds in models of advanced DN (with established glomerular/tubulointerstitial damage) remains untested—whether these interventions can reverse existing injury or only delay progression is unknown. 4) Underexplored therapeutic strategies and mechanisms: Despite promising preclinical data on natural compounds and TCM formulas, their molecular targets and pharmacokinetic properties are poorly defined. Most TCM studies have focused on downstream effects (e.g., upregulating Nrf2/GPX4) but fail to identify active ingredients or their direct binding partners, hindering standardized development and clinical approval. Additionally, combination therapies targeting ferroptosis with conventional DN treatments (e.g., SGLT2 inhibitors and RAS blockers) are understudied; whether synergistic effects exist (e.g., empagliflozin enhancing Nrf2-mediated ferroptosis inhibition) and potential antagonism require evaluation. Furthermore, renal-specific drug delivery systems for ferroptosis modulators are lacking; systemic administration may cause off-target effects (e.g., promoting tumorigenesis via global ferroptosis inhibition) or insufficient renal tissue penetration, which has not been addressed in preclinical studies. 5) Gaps in understanding ferroptosis in DN comorbidities: DN patients often present with comorbidities (e.g., hypertension, obesity, and dyslipidemia) that exacerbate renal injury, but how these comorbidities interact with ferroptosis in DN is unexplored. For example, whether hypertension-induced renal hypoxia amplifies high glucose-mediated ferroptosis, or if dyslipidemia synergizes with iron overload to accelerate lipid peroxidation, remains unclear. Additionally, the impact of anti-diabetic drugs (beyond SGLT2 inhibitors) on renal ferroptosis is understudied—whether insulin or GLP-1 agonists directly modulate ferroptosis pathways or indirectly via glycemic control requires clarification. These gaps highlight the need for future research to move beyond descriptive mechanisms toward systematic dissection of pathway crosstalk, cell type-specific functions, and clinical validation. Addressing these areas will not only deepen the understanding of ferroptosis in DN but also facilitate the development of precise, translatable therapeutic strategies for this globally prevalent cause of ESRD.

### 3.4 Ferroptosis–renal fibrosis crosstalk: current research

CKD is a global public health challenge, with renal fibrosis (RF)—characterized by fibroblast/extracellular matrix (ECM) accumulation, glomerulosclerosis, and tubular atrophy (187)—as its common progressive hallmark. RF leads to irreversible renal function decline and ESRD, but treatment options remain limited and costly (188). Ferroptosis and RF exhibit a mutually reinforcing relationship: in CKD renal tissues, lipid peroxidation, necroinflammation, and iron overload damage parenchymal cells, driving fibrotic accumulation (189). Proximal tubule (PT) cells, in a pro-inflammatory state, are highly susceptible to ferroptosis; even mild ferroptosis impairs renal repair and triggers RF, while ferroptosis also promotes fibroblast differentiation to accelerate RF (190).

#### 3.4.1 Ferroptosis modulation interferes with RF

Wang et al. (191): In CKD rats, ferroptosis inducer cisplatin (CP) reduced GSH and GPX4, while inhibitor desferrioxamine (DFO) elevated these markers, confirming ferroptosis modulation as an RF intervention. Feng et al. (192): Ferroptosis inhibitor-1 reduced kidney injury molecule 1 (KIM-1), HIF-1α, HO-1, and ROS in db/db mouse kidneys, indicating that ferroptosis drives RF/renal dysfunction via the HIF-1/HO-1 pathway. Ide et al. (193): Iron overload in severely damaged PT cells triggers ferroptosis and inflammatory PT cell aggregation; persistent inflammation fuels RF, linking ferroptosis to renal injury amplification. Zhou et al. (194): In IRI *vs*. unilateral ureteral obstruction (UUO) mice, GPX4 declined faster post-IRI and failed to recover by day 28, reflecting model-specific ferroptosis kinetics. Ferroptosis inhibitors [ferrostatin-1 (Fer-1) and DFO] reduced tubular inflammatory chemotaxis to alleviate RF (195), and Lip-1 (ferroxidase inhibitor) mitigated UUO-induced RF by preserving GPX4 and suppressing pro-fibrotic factors. These studies, combined with CKD patient renal biopsies, confirm renal ferroptosis as a key RF driver; inhibiting ferroptosis promotes tissue remodeling and delays RF.

#### 3.4.2 RF induces ferroptosis: a vicious cycle

RF and ferroptosis form a self-perpetuating loop: 1) RF-driven CKD progression reduces renal function, causing hematuria, renal anemia, and increased tubular iron filtration/exogenous iron intake, leading to iron overload and ferroptosis. 2) Ferroptosis triggers DAMP release, inflammation, and immune activation; injured tubules activate fibroblasts or undergo EMT to exacerbate RF, further reducing glomerular filtration rate (116). 3) PT cells post-injury downregulate ferroptosis defense pathways, becoming hypersensitive to ferroptosis. Even mild injury prevents PT redifferentiation, promoting inflammatory PT cell aggregation and maladaptive repair via pro-fibrotic signals, extending ferroptosis’s role beyond cell death to non-lethal pathological cell state regulation.

#### 3.4.3 Targeting ferroptosis to ameliorate RF

Ferroptosis is now recognized as a pivotal regulated necrotic pathway in AKI (196), and recurrent AKI drives maladaptive repair—marked by tubular atrophy, fibroblast activation, and excessive ECM deposition—culminating in CKD and ESRD, where RF is nearly universal. TECs, central to both injury sensing and myofibroblast generation (197), initiate fibrogenic signaling, while myofibroblasts execute matrix accumulation (198). In UUO—a canonical RF model—TECs display hallmark ferroptotic features (199). Treatment with the ferroptosis inhibitor Lip-1 significantly reduces renal iron and MDA levels, attenuating fibrosis and dysfunction (116). Mechanistically, GPX4 inhibition in HK-2 cells triggers iron-dependent release of pro-necrotic mediators that promote fibroblast proliferation and myofibroblast differentiation, effects reversed by Lip-1 (189). Similarly, platycodin and Fer-1 mitigate TEC injury and suppress TGF-β1-driven fibrogenesis in UUO (191). Beyond direct cytoprotection, Fer-1 and the iron chelator DFO dampen inflammatory cell infiltration in tubules, further limiting fibrosis. Notably, specific ferroptosis inhibitors show efficacy not only in RF models but also in rodent models of kidney and liver cancer (195), underscoring ferroptosis in TECs as a therapeutically tractable node in fibrogenesis.

#### 3.4.4 Targeting ferroptosis for the treatment of renal fibrosis

Ferroptosis inhibition holds substantial therapeutic promise for kidney disease, with research accelerating since its discovery in 2012; numerous inducers and inhibitors have been identified, some in clinical trials (200). Here, we focus on classical natural active components from TCM as ferroptosis inhibitors, highlighting their mechanisms in renal disease. Preclinical studies have confirmed that targeted ferroptosis inhibition alleviates renal injury and fibrosis: 1) deferoxamine mitigates 5/6 nephrectomy-induced CKD in rats via iron metabolism and TGF-β1/Smad3 pathway modulation (191). 2) Lip-1 reduces collagen deposition and pro-fibrotic factor expression in UUO mice and suppresses paracrine-driven fibroblast activation in human proximal tubular epithelial (HK-2) cells (116). 3) Fer-1 and deferoxamine inhibit tubular epithelial ferroptosis, alleviating UUO- or I/R-induced injury and fibrosis (200). 4) Berberine activates AMPK to reduce lipid peroxidation and ferroptosis, mitigating I/R-induced renal fibrosis in mice (201). 5) Irisflorentin blocks erastin/RSL3-induced ferroptosis and TGF-β1-stimulated fibrosis in primary tubular cells and suppresses Smad3 phosphorylation/Nox4 to reduce UUO-induced injury and fibrosis (202). Additionally, Balzer et al. (203) identified pyroptosis/ferroptosis as vulnerable pathways in pro-fibrotic proximal tubule clusters during maladaptive renal regeneration; their targeting promoted adaptive repair and reduced fibrosis. Collectively, these findings support ferroptosis as a therapeutic target to prevent renal fibrosis in CKD.

GPX4 is critical in this context. Renal TECs rely on FAO for energy, with FAO–fibrosis crosstalk: FAO blockade promotes lipid deposition during fibrosis, and TGF-β reduces FAO to enhance lipid accumulation (204, 205). TGF-β1-induced tubular lipid peroxidation (linked to renal failure) is reversed by GPX4 (206), while GPX4 deficiency exacerbates TGF-β1 production and fibrosis—effects reversed by GPX4 upregulation (207). Elevated GPX4 also attenuates NF-κB activation (208) and suppresses fibroblast IL-6 release (209), mitigating fibrosis. In CKD patient biopsies and UUO/I/R models, GPX4 downregulation and 4-HNE upregulation highlight ferroptosis’s role in tubular pathology (200). Concordantly, Lip-1 preserves GPX4 to reduce iron deposition/lipid peroxidation and suppress ferroptosis; erastin promotes myofibroblast differentiation via lipid peroxidation and GPX4 inhibition, while Fer-1 reverses this by enhancing GPX4 (210). These data establish GPX4 as a key target for ferroptosis-mediated fibrosis alleviation, although mechanistic details require further clarification.

While accumulating evidence confirms a mutually reinforcing crosstalk between ferroptosis and RF and validates ferroptosis inhibition as a potential therapeutic strategy, several critical, insufficiently addressed research gaps persist; these gaps limit a comprehensive understanding of the pathogenic network and hinder translational progress, necessitating critical dissection to enhance the review’s depth. 1) Mechanistic gaps in crosstalk integration and novel regulatory nodes: Current research establishes a bidirectional link between ferroptosis and RF but lacks insights into the core molecular hubs that integrate this crosstalk. For instance, how ferroptosis-induced DAMPs (e.g., HMGB1) specifically activate fibroblast differentiation or EMT remains poorly defined; whether these DAMPs directly bind to fibroblast receptors or act via intermediate inflammatory signals has not been rigorously elucidated. Additionally, the role of non-classical ferroptosis regulators (e.g., FSP1, DHODH, and GCH1/BH4) in RF is largely unexplored; it is unknown whether these radical-trapping antioxidant pathways compensate for GPX4 deficiency in fibrotic kidneys or if their dysfunction exacerbates ferroptosis–RF crosstalk. Furthermore, the interplay between ferroptosis and other fibrogenic pathways (e.g., TGF-β/Smad and Wnt/β-catenin) is fragmented; whether TGF-β1 directly induces tubular ferroptosis via iron overload or lipid peroxidation, or indirectly by suppressing GPX4/FSP1, requires definitive validation. The molecular basis for model-specific ferroptosis kinetics [e.g., faster GPX4 decline in IRI *vs*. UUO (194)] also remains unclear, raising questions about the generalizability of mechanisms across different RF etiologies. 2) Cell type- and etiology-specific regulatory blank spots: Most studies have focused on TECs and fibroblasts, but the role of renal non-parenchymal cells in ferroptosis–RF crosstalk is severely understudied. For example, whether mesangial cell ferroptosis contributes to glomerulosclerosis (a key feature of RF) or if renal endothelial ferroptosis exacerbates microvascular rarefaction and hypoxia (further amplifying RF) has not been systematically characterized. Additionally, RF arises from diverse etiologies (e.g., diabetic nephropathy, hypertensive nephropathy, and chronic interstitial nephritis), but ferroptosis mechanisms across these etiologies are rarely compared. It is unknown whether the core drivers of ferroptosis–RF crosstalk (e.g., iron overload *vs*. antioxidant exhaustion) vary by etiology, which limits the development of etiology-tailored therapies. Moreover, the contribution of ferroptosis in “non-lethal pathological cell states” [e.g., inflammatory PT cell aggregation (116)] to RF progression is poorly defined; how these states perpetuate fibrogenesis without inducing overt cell death requires further investigation. 3) Limitations in clinical translation and biomarker development: A major translational barrier is the absence of RF-specific ferroptosis biomarkers with clinical utility. Current markers (e.g., GPX4, 4-HNE, and serum iron) lack specificity for ferroptosis-driven RF, as they are also altered in other kidney diseases or metabolic disorders. Validating non-invasive biomarkers (e.g., urine exosomal ferroptosis-related proteins and circulating lipid peroxide metabolites) that reflect renal ferroptosis activity in CKD patients with RF is urgently needed for early diagnosis and therapeutic monitoring. Furthermore, most preclinical studies have used acute RF models (e.g., UUO and I/R), which fail to recapitulate the chronic, progressive nature of human RF (e.g., long-term fibrosis and comorbidity-associated complications). The efficacy of ferroptosis inhibitors (e.g., Lip-1 and Fer-1) in models of chronic RF (e.g., diabetic nephropathy-related fibrosis and hypertensive nephropathy-related fibrosis) remains untested; whether these interventions can reverse established fibrosis or only delay progression is unknown. Additionally, the long-term safety of ferroptosis inhibitors (e.g., risk of promoting tumorigenesis via global ferroptosis suppression) and their pharmacokinetic profiles in fibrotic kidneys (e.g., tissue penetration and metabolism) have not been evaluated. 4) Underexplored therapeutic strategies and model limitations: Despite promising preclinical data, combination therapeutic strategies targeting ferroptosis–RF crosstalk are understudied. For example, whether combining ferroptosis inhibitors with anti-fibrotic agents (e.g., TGF-β inhibitors and pirfenidone) yields synergistic effects remains untested, as does the potential for antagonism between these agents. Natural compounds and TCM formulas [e.g., berberine and irisflorentin (201, 202)] show efficacy, but their active ingredients, direct molecular targets, and standardized formulations are poorly defined, hindering clinical development. Moreover, preclinical RF models exhibit significant limitations: UUO and I/R models induce acute severe fibrosis that does not mimic the gradual progression of human CKD-related RF and lacks comorbidities (e.g., diabetes and hypertension) that are common in clinical settings. This model dependence raises concerns about the translatability of findings, as mechanisms identified in acute models may not apply to chronic, clinically relevant RF. 5) Gaps in understanding ferroptosis–fibrosis crosstalk in adaptive *vs*. maladaptive repair: The role of ferroptosis in distinguishing adaptive *vs*. maladaptive renal repair—critical for RF progression—remains unclear. Mild ferroptosis is proposed to impair repair (190), but it is unknown whether there is a “threshold” of ferroptosis that switches repair from adaptive to maladaptive, or which molecular signals govern this switch. Additionally, the impact of ferroptosis on renal regeneration (e.g., proliferation of renal progenitor cells) in fibrotic kidneys is entirely unexplored; whether ferroptosis inhibits regeneration by damaging progenitor cells or indirectly via inflammatory signals requires investigation.

These gaps highlight the need for future research to move beyond descriptive associations toward systematic dissection of integrative mechanisms, cell type-specific functions, and clinical validation. Addressing these areas will not only deepen the understanding of ferroptosis–RF crosstalk but also facilitate the development of precise, translatable therapeutic strategies for CKD-related RF, an unmet clinical need.

### 3.5 Ferroptosis in SLE nephritis: core advances

Systemic lupus erythematosus (SLE) is a prototypic autoimmune disease characterized by self-tolerance loss, autoantigen/immune complex formation, and multi-organ inflammation (211). Lupus nephritis (LN)—the most severe SLE manifestation—affects most patients within 5 years of diagnosis, with ~10% progressing to end-stage kidney disease (ESKD) (212). Dysregulated iron metabolism is a key pathogenic feature of LN (213), and emerging evidence links these disturbances to ferroptosis (214)—an iron-dependent cell death driven by lipid peroxidation, distinct from apoptosis/necrosis (82). Although still in the early stage, ferroptosis research opens new avenues for LN pathogenesis and therapy.

#### 3.5.1 CD4 T-cell iron dyshomeostasis drives SLE/LN

Iron overload promotes pathogenic T-cell differentiation and accelerates disease in lupus-prone models (215): 1) In MRL/MpJ-Fas (lpr)/J (MRL/lpr) mice, iron overload expands T follicular helper (Tfh) cells, germinal center (GC) B cells, and pro-inflammatory cytokine-secreting CD4 T cells. 2) This amplifies autoantibody production and T cell-dependent (TD) humoral responses, directly fueling SLE/LN progression. 3) Maintaining iron homeostasis is critical for eliminating pathogenic Th cells and balancing protective *vs*. autoimmune humoral immunity.

#### 3.5.2 GPX4-regulated neutrophil ferroptosis triggers autoimmunity

Neutrophils—central effectors of innate immunity—are now implicated in the pathogenesis of SLE not only through classical functions (e.g., autoantibody formation and TNF-α release) but also via dysregulated iron metabolism and ferroptosis. Recent work reveals that GPX4-controlled ferroptosis in neutrophils drives systemic autoimmunity by unleashing redox-active iron, which amplifies inflammatory and immune activation, a hallmark of SLE (216). GPX4 sustains neutrophil integrity by detoxifying lipid hydroperoxides in a GSH-dependent manner. Its loss triggers ferroptosis, leading to plasma iron release and subsequent immune stimulation (216). In murine models, GPX4 deficiency in neutrophils results in iron overload and spontaneous autoimmune responses resembling SLE. Critically, neutrophils from SLE patients exhibit heightened ferroptotic signatures, correlating with disease activity. The genetic or pharmacological restoration of GPX4 suppresses neutrophil ferroptosis and abrogates downstream autoimmunity, underscoring GPX4 as a checkpoint that constrains iron-driven inflammation (206, 217). These findings position neutrophil ferroptosis—orchestrated by GPX4—as a mechanistic bridge between iron dyshomeostasis and loss of self-tolerance in SLE, offering a rationale for therapeutic strategies targeting neutrophil redox resilience or iron chelation in autoimmune disease.

#### 3.5.2 B-cell ferroptosis modulates LN severity

B-cell ferroptosis is detectable in SLE patients and MRL/lpr mice (218): Treatment with ferroptosis inhibitor Lip-1 reduces autoantibody production, ameliorates renal damage, and alleviates lupus symptoms *in vivo*. Ferroptosis regulates B-cell differentiation and plasma cell formation, directly linking this cell death pathway to humoral autoimmunity in LN.

#### 3.5.4 Macrophage ferroptosis and SLE

Renal M2-like macrophages in LN patients selectively express CD163 (haptoglobin–hemoglobin complex scavenger) and SLC40A1, tied to iron homeostasis (219, 220): 1) SLE-associated autoimmune hemolysis and glomerular injury-induced erythrocyte lysis generate excess haptoglobin–hemoglobin complexes. 2) Abnormally elevated CD163+ macrophages drive excessive complex phagocytosis, leading to macrophage iron overload, LIP expansion, oxidative stress, and ferroptosis. 3) Clinical relevance: LN patients with Grade III/IV disease show higher ferroptosis levels, which correlate strongly with renal function decline (221). 4) CD163 and PC are dysregulated genes validated to associate with lipid peroxidation; ferroptosis inhibitors improve LN in lupus-prone mice (221). Ferroptosis—regulated by iron metabolism and lipid peroxidation (222)—plays a non-redundant role in SLE/LN via multiple immune cell types: T/B-cell iron overload triggers ferroptosis to amplify autoimmunity (215, 218); neutrophil/macrophage ferroptosis fuels renal inflammation and injury (216, 219). Mitochondrial oxidative stress and DNA damage link ferroptosis to SLE pathogenesis (223). Therapeutic potential: Iron inhibitors and antioxidants reduce SLE/LN inflammation (224), with preclinical efficacy of ferroptosis blockers (e.g., Lip-1) (218, 221). Unresolved issues were as follows: 1) crosstalk between ferroptosis and other cell death pathways (e.g., pyroptosis) in LN, 2) interplay of iron metabolism with epigenetic regulation in ferroptosis-driven autoimmunity, and 3) development of LN-specific ferroptosis inhibitors and sensitive renal biomarkers for clinical translation (225).

While emerging evidence establishes ferroptosis as a pivotal mediator of SLE nephritis (LN) pathogenesis—linking iron dyshomeostasis, immune cell dysfunction, and renal injury—and identifies GPX4 as a key regulatory checkpoint, several critical, insufficiently addressed research gaps persist. These gaps limit a comprehensive understanding of ferroptosis-driven LN progression and hinder translational progress, necessitating critical dissection to enhance the review’s depth and clinical relevance. 1) Mechanistic gaps in cell–cell crosstalk and non-classical regulatory pathways: Current research focuses on ferroptosis in individual immune cell types (T cells, neutrophils, B cells, and macrophages) but lacks insights into the intercellular crosstalk that amplifies ferroptosis-driven autoimmunity and renal injury in LN. For instance, it remains unclear how ferroptosis in neutrophils [e.g., GPX4 deficiency-induced iron release (216)] directly modulates T/B-cell differentiation or macrophage activation, or whether DAMPs released by ferroptotic immune cells propagate ferroptosis in renal parenchymal cells (e.g., tubular epithelial cells). Additionally, the role of non-classical ferroptosis regulators (e.g., FSP1, DHODH, and GCH1/BH4) in LN is entirely unexplored—whether these radical-trapping antioxidant pathways compensate for GPX4 deficiency in immune cells or renal tissue, or if their dysfunction exacerbates ferroptosis susceptibility, has not been investigated. The molecular link between mitochondrial oxidative stress/DNA damage and ferroptosis in LN (223) is also poorly defined: it is unknown whether mitochondrial dysfunction directly triggers iron overload/lipid peroxidation or indirectly by suppressing antioxidant networks (e.g., Nrf2/GPX4). Furthermore, the epigenetic mechanisms underlying ferroptosis-driven autoimmunity (225) remain vague; specific epigenetic modifications (e.g., DNA methylation and histone acetylation) that regulate ferroptosis-related genes (e.g., GPX4 and SLC7A11) in LN immune cells or renal tissue have not been systematically mapped.

2) Cell type- and pathological grade-specific regulatory blank spots: Most studies have centered on immune cells, but the role of renal parenchymal cells (e.g., tubular epithelial cells, mesangial cells, and renal endothelial cells) in ferroptosis-driven LN is severely understudied. For example, whether mesangial cell ferroptosis contributes to glomerulosclerosis (a core pathological feature of LN) or if tubular epithelial cell ferroptosis amplifies renal inflammation and fibrosis in LN has not been rigorously characterized. Additionally, LN exhibits distinct pathological grades (I–V) with varying immune infiltration and renal damage, but ferroptosis mechanisms across these grades are poorly differentiated. While Grade III/IV LN patients show higher ferroptosis levels (221), it is unknown whether the core drivers of ferroptosis (e.g., iron overload *vs*. antioxidant exhaustion) or key cell types mediating ferroptosis vary by grade; this limits the development of grade-tailored therapies. The role of ferroptosis in LN remission and relapse is also unexplored; whether residual ferroptosis in immune cells or renal tissue predisposes patients to disease recurrence remains unaddressed. 3) Limitations in clinical translation and biomarker development: A major translational barrier is the lack of LN-specific, non-invasive ferroptosis biomarkers with clinical utility. Current markers (e.g., GPX4 expression, serum iron, and lipid peroxides) lack specificity for LN, as they are altered in other autoimmune diseases or kidney disorders. Validating urine-based biomarkers (e.g., exosomal ferroptosis-related proteins and lipid peroxide metabolites) or circulating immune cell ferroptosis signatures that correlate with LN disease activity, pathological grade, or treatment response is urgently needed. Furthermore, existing ferroptosis inhibitors [e.g., Lip-1 (218, 221)] are broad-spectrum, and their efficacy in targeting LN-specific ferroptosis (e.g., immune cell *vs*. renal tissue ferroptosis) has not been evaluated. The safety and efficacy of combining ferroptosis inhibitors with conventional LN therapies (e.g., glucocorticoids and immunosuppressants) are also untested; whether synergistic effects reduce immunosuppressant doses or potential antagonism (e.g., immunosuppressants altering iron metabolism/ferroptosis) exists remains unknown. Additionally, the impact of SLE comorbidities (e.g., lupus nephritis with hypertension and metabolic syndrome) on ferroptosis pathways in LN is understudied; whether comorbidities exacerbate ferroptosis by disrupting iron homeostasis or antioxidant capacity requires investigation. 4) Underexplored therapeutic strategies and model limitations: Preclinical studies have relied heavily on lupus-prone mouse models (e.g., MRL/lpr mice), which may not fully recapitulate human LN’s complexity (e.g., genetic heterogeneity, variable disease progression, and response to therapy). The translatability of ferroptosis-targeted therapies from mice to humans is thus uncertain, for example, whether human LN immune cells exhibit the same ferroptosis susceptibility as mouse models, or if species-specific differences in ferroptosis regulators (e.g., FSP1 *vs*. GPX4 dependence) exist. Furthermore, LN-specific ferroptosis inhibitors (225) are lacking; current agents target general ferroptosis pathways, raising risks of off-target effects (e.g., promoting tumorigenesis via global ferroptosis suppression). Natural compounds or TCM formulas with ferroptosis-inhibitory effects in other kidney diseases [e.g., berberine and irisflorentin (201, 202)] have not been tested in LN, representing an untapped therapeutic avenue. Finally, the role of ferroptosis in LN chronicization—whether persistent ferroptosis in immune cells or renal tissue drives the transition from acute LN to CKD with irreversible renal fibrosis—has not been explored.

These gaps highlight the need for future research to move beyond single-cell type and descriptive mechanisms toward systematic dissection of intercellular crosstalk, renal parenchymal cell contributions, and clinical validation. Addressing these areas will not only deepen the understanding of ferroptosis in LN but also facilitate the development of precise, translatable biomarkers and therapies for this severe autoimmune kidney disease.

### 3.6 Ferroptosis: a selective vulnerability of renal cell carcinoma

RCC accounts for 90% of renal cancers, with three dominant subtypes: clear cell (ccRCC; 70%), papillary (pRCC; 10%–15%), and chromophobe (ChRCC; 5%) (226). RCC exhibits unique susceptibility to ferroptosis-iron-dependent lipid peroxidation-driven cell death (82), distinguishing it from normal kidney tissue. 1) Sensitivity evidence: RCC cells are more vulnerable to erastin-induced death than other tumor types, with ROS accumulation and GPX4 downregulation reversed by antioxidants (227). 2) Transcriptomic signature: TCGA/GTEx analyses show upregulated SLC7A11/FSP1 and downregulated ACSL4 (key ferroptosis regulators) across RCC subtypes (18). 3) Subtype-specific mechanisms: ccRCC: GPX4 silencing induces lipid peroxidation and cell loss (227); p53/BAP1 suppresses SLC7A11 to promote ferroptosis (54, 228, 229). ChRCC: High GSH/GSSG levels enhance ferroptosis inducer sensitivity (230). Hereditary Leiomyomatosis and Renal Cell Carcinoma (HLRCC): FH inactivation causes fumarate accumulation, protein succination, and GPX4 dysfunction (231). Microenvironmental regulation: Tumor cell density modulates ferroptosis via the TAZ–EMP1–NOX4 axis (232). Therapeutic implication: Ferroptosis inducers (erastin, sorafenib, and artesunate) inhibit RCC progression (227, 233); ferroptosis-related genes (GPX4 and ACSL4) serve as prognostic biomarkers (233), supporting precision therapy development. Ferroptosis contributes to the initiation, progression, and metastasis of renal cancer. Renal carcinoma cells exhibit heightened ferroptotic sensitivity compared to normal kidney cells, a vulnerability that can be therapeutically exploited to spare healthy tissue and overcome drug resistance through rational combination strategies. Targeting ferroptosis thus offers a promising avenue for precision oncology in renal cancer, warranting further development of selective inducers and biomarker-guided therapeutic regimens.

#### 3.6.1 Natural compounds: ferroptosis activators with tumor selectivity

Natural compounds selectively induce ferroptosis in renal cancer cells while sparing normal kidney tissue, offering a therapeutic window for precision oncology. Icariin II (ICS II), a flavonoid from *Epimedium koreanum*, triggers ferroptosis in renal carcinoma by downregulating GPX4 in a p53-independent manner and upregulating miR-324-3p, which suppresses GPX4 expression, leading to iron accumulation, lipid peroxidation, and GSH depletion (234). Despite promising *in vitro* and *in vivo* efficacy, validation remains limited to two cell lines and animal models. Artesunate (ART), an artemisinin derivative, overcomes sunitinib resistance in renal cancer, with ferroptosis observed specifically in KTCTL-26 cells via ROS burst, metabolic rewiring, and p53 induction, suggesting p53 as a potential biomarker for ART responsiveness (235, 236). ART’s broader anti-tumor activity in non-urological cancers further supports its repurposing potential (237–240). Lycorine, an Amaryllidaceae alkaloid with low toxicity, reduces GPX4 and elevates ACSL4 in renal cancer cells; these effects are reversed by ferrostatin-1, implicating ferroptosis as a key mechanism (241). However, evidence is confined to cell-based studies (242–244). Luteolin (Lut), a dietary flavonoid, induces hallmark ferroptotic features such as mitochondrial shrinkage, iron overload, GSH loss, and lipid peroxidation via HO-1 overexpression and LIP activation; inhibition by DFO or ferrostatin-1 confirms ferroptosis dependence (245, 246). Broader validation across renal cancer subtypes and clinical translation remain pending. Finally, salinomycin (Sal)–originally identified in a high-throughput screen for CSC-targeting agents (247, 248)—sensitizes renal cancer to ferroptosis by downregulating PDIA4 via autophagy, thereby suppressing the ATF4–SLC7A11–GPX4 axis (249, 250). Notably, PDIA4 overexpression correlates with poor prognosis in RCC, positioning it as both a biomarker and a node for Sal-mediated intervention. Collectively, these natural compounds exploit the heightened ferroptotic vulnerability of renal cancer cells through distinct but convergent pathways centered on GPX4 suppression. Their synergy with existing therapies (240, 251, 252) and selectivity for malignant over normal cells underscore their potential as next-generation ferroptosis-inducing agents, although rigorous clinical validation is still required.

#### 3.6.2 Ferroptosis dysregulation drives RCC drug resistance

Sorafenib, the first Food and Drug Administration (FDA)-approved multi-kinase inhibitor for metastatic ccRCC (253), was initially shown to induce ferroptosis by inhibiting SLC7A11 (254). However, acquired resistance—now a major clinical limitation—has been linked to DPP9 overexpression (255). DPP9, a proline-specific dipeptidase upregulated in ccRCC and associated with poor prognosis (255), binds KEAP1 via a conserved ESGE motif, competitively disrupting KEAP1-mediated ubiquitination and degradation of NRF2 (256). Stabilized NRF2 transcriptionally upregulates SLC7A11, restoring cystine uptake and GSH synthesis, thereby conferring ferroptosis resistance and directly counteracting sorafenib’s mechanism of action. Genetic ablation of *DPP9* reverses this resistance *in vivo* and in organoids, suggesting that small-molecule disruption of the DPP9–KEAP1 interface could resensitize NRF2-hyperactive tumors to ferroptosis-inducing therapy. Similarly, resistance to sunitinib—another frontline tyrosine kinase inhibitor—remains a critical unmet need (257). Recent work implicates the AIM2 inflammasome, which is overexpressed in ccRCC and drives sunitinib resistance (258). AIM2 suppresses ferroptosis by promoting proteasomal degradation of FOXO3a, a transcriptional activator of *ACSL4*—a key enzyme that esterifies PUFAs into peroxidation-prone phospholipids essential for ferroptosis execution (259). By dampening the FOXO3a–ACSL4 axis, AIM2 reduces membrane PUFA-PL content and blunts lipid peroxidation, enabling tumor cells to evade ferroptosis. Notably, AIM2 confers resistance even in treatment-naïve cells, positioning it as both a biomarker and a therapeutic target. Together, these findings reveal that ferroptosis suppression via NRF2 activation or ACSL4 inhibition underpins resistance to standard targeted therapies in ccRCC. Restoring ferroptotic sensitivity—by targeting DPP9, AIM2, or their downstream effectors—offers a rational strategy to overcome drug resistance and improve outcomes in advanced renal cancer.

#### 3.6.3 Combination therapy: restoring ferroptosis sensitivity

Everolimus—an FDA-approved mTOR inhibitor used as second-line therapy for metastatic RCC (mRCC) resistant to sorafenib/sunitinib—now faces resistance challenges. However, combining everolimus with ferroptosis inducers (erastin, which inhibits System Xc^−^; and RSL3, which targets GPX4) overcomes this limitation: the combination suppresses the mTOR–4EBP1 axis to inhibit RCC cell activity and induce ferroptosis, offering a promising option for drug-resistant mRCC (260). Another synergistic combination involves the ferroptosis inducer RSL3 and URB597 (a potent oral FAAH inhibitor). Fatty Acid Amide Hydrolase (FAAH)—an upregulated serine hydrolase in tumors—regulates cancer proliferation, and its inhibitors exhibit anti-invasive/anti-metastatic effects, often synergizing with chemotherapeutics (261, 262); importantly, FAAH inhibitors are well-tolerated in clinical trials (263). In RCC, GPX4 (a key ferroptosis enzyme) correlates with FAAH expression. The URB597–RSL3 combination targets both, reducing RCC cell viability via ferroptosis and G1 cell cycle arrest and exerting stronger effects on ferroptosis-related genes, proliferation, and invasion than single agents (264). Combining ferroptosis inducers with existing RCC therapies thus represents a feasible strategy, with potential for more such regimens to advance RCC treatment.

**Figure 5:**
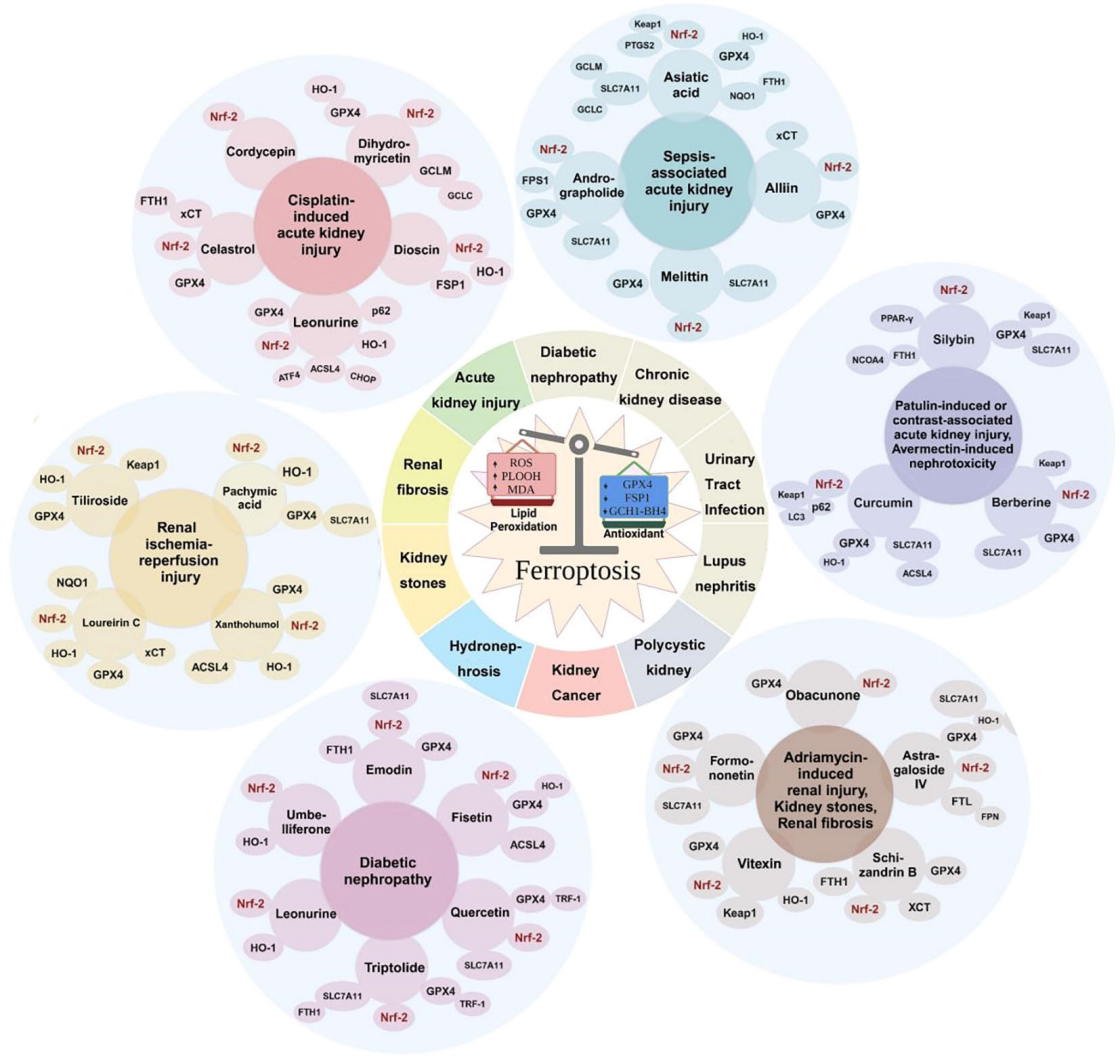
Natural compounds modulating ferroptosis in kidney diseases. The central role of ferroptosis and its key regulators (e.g., NRF2 and GPX4) in diverse kidney diseases. This schematic illustrates the intricate relationship between ferroptosis and a wide spectrum of renal pathologies. At the center, ferroptosis is depicted as a pivotal cell death pathway driven by lipid peroxidation (indicated by ROS, PLOOH, MDA) and counteracted by antioxidant systems, prominently featuring GPX4 and its cofactor GSH, alongside other regulators like FSP1 and CoQ10. Surrounding this core are eight distinct kidney disease states, each represented by a colored circle. Within each circle, key molecules and compounds known to modulate ferroptosis in that specific context are listed. Crucially, NRF2 (highlighted in red text) and GPX4 (highlighted in blue text) are shown to be central, frequently recurring regulators across nearly all disease conditions, underscoring their fundamental importance in renal ferroptosis. The figure highlights how various insults—such as cisplatin, sepsis, ischemia–reperfusion, diabetes, contrast agents, adriamycin, or heavy metals—can trigger ferroptosis, contributing to acute kidney injury (AKI), chronic kidney disease (CKD), fibrosis, cancer, stones, and other nephropathies. The presence of numerous pharmacological agents (e.g., ferrostatin-1, liproxstatin-1, silybin, curcumin, and tiliroside) within these circles also suggests potential therapeutic strategies targeting the NRF2/GPX4 axis to mitigate ferroptosis-driven kidney damage.

Ferroptosis is tightly linked to iron/lipid metabolic disorders and antioxidant system dysfunction—key contexts for RCC—as RCC cells exhibit inherent iron accumulation and abnormal amino acid/lipid metabolism. Therapeutically, traditional Chinese medicine and natural compounds act as ferroptosis activators, offering new candidates for RCC treatment. Ferroptosis inducers also reverse drug resistance when combined with standard RCC therapies; notably, novel nanomaterials targeting excessive iron in tumor cells to trigger ferroptosis open innovative avenues for RCC intervention. Critical gaps remain; however, no clinical trials of ferroptosis-based RCC treatments exist due to insufficient understanding of ferroptosis’s functions and mechanisms. Additionally, existing preclinical studies lack validation of drug efficacy across multiple RCC cell lines. Thus, large-scale experiments are required to fully establish the biosafety and reliability of ferroptosis-targeting agents, while verifying their role in RCC progression and treatment, before clinical translation. The mechanisms by which natural compounds modulate ferroptosis in the intervention of kidney diseases, such as DN, are illustrated in **Figure 5**.

While compelling evidence establishes ferroptosis as a selective vulnerability of RCC and validates its potential for therapeutic exploitation—via natural compounds, reversal of drug resistance, and combination regimens—several critical, insufficiently addressed research gaps persist. These gaps limit a comprehensive understanding of ferroptosis-driven RCC pathogenesis and hinder translational progress, necessitating a critical dissection to enhance the review’s depth and clinical relevance. 1) Mechanistic gaps in non-classical pathways and tumor microenvironment (TME) crosstalk: Current research centers on classical ferroptosis regulators (GPX4, SLC7A11, and ACSL4) but overlooks the role of non-classical ferroptosis suppressors (FSP1, DHODH, and GCH1/BH4) in RCC. For instance, it remains unknown whether FSP1 or DHODH compensates for GPX4 deficiency in RCC subtypes [e.g., ccRCC with GPX4 silencing (227)] or if their dysregulation contributes to ferroptosis resistance; this limits the development of pan-ferroptosis targeting strategies. Additionally, the molecular basis of subtype-specific ferroptosis sensitivity [e.g., ChRCC’s high GSH/GSSG levels yet enhanced inducer sensitivity (230)] is poorly defined; whether other antioxidant pathways or lipid metabolic features (e.g., PUFA composition) offset GSH abundance requires clarification. The crosstalk between tumor cell ferroptosis and the TME is also fragmented: how ferroptotic RCC cells modulate immune cell function (e.g., macrophage polarization and T-cell activation) or how immune cells in the TME (e.g., tumor-associated neutrophils) regulate RCC ferroptosis remains unexplored. Furthermore, while tumor cell density modulates ferroptosis via the TAZ–EMP1–NOX4 axis (232), the upstream triggers of this axis in RCC (e.g., hypoxia and ECM stiffness) and its interplay with other TME signals (e.g., cytokines and growth factors) are not elucidated. 2) Subtype-specific and genotype-driven regulatory blank spots: Most studies have focused on ccRCC, leaving other RCC subtypes (pRCC, ChRCC, and HLRCC) severely understudied. For example, pRCC’s ferroptosis regulation beyond p53/BAP1-mediated SLC7A11 suppression (54, 228, 229) is unknown; whether it shares ccRCC’s dependence on GPX4 or exhibits unique drivers (e.g., different lipid metabolic enzymes) remains untested. HLRCC’s FH inactivation-induced fumarate accumulation and GPX4 dysfunction (231) also lack downstream mechanistic dissection; whether fumarate directly modifies GPX4 or acts via other succination targets to drive ferroptosis is unclear. Additionally, the impact of RCC driver mutations (e.g., VHL, PBRM1, and SETD2 in ccRCC) on ferroptosis pathways is incompletely characterized. For instance, VHL loss leads to HIF stabilization, but how HIF regulates ferroptosis (e.g., via iron metabolism genes and antioxidant networks) in RCC is not systematically mapped. This subtype and genotype limit the development of precision ferroptosis-targeted therapies tailored to RCC’s molecular heterogeneity. 3) Limitations in clinical translation and biomarker development: A major translational barrier is the absence of RCC-specific, clinically actionable ferroptosis biomarkers. Current markers [GPX4 and ACSL4 (233)] lack predictive power for ferroptosis inducer responsiveness or drug resistance—they cannot distinguish which patients will benefit from sorafenib, natural compounds [e.g., artesunate (235)], or combination regimens. Validating robust, non-invasive biomarkers (e.g., circulating lipid peroxide metabolites, urine exosomal ferroptosis regulators, or TME-derived immune cell ferroptosis signatures) is urgently needed to guide clinical trial design and patient stratification. Furthermore, no clinical trials of ferroptosis-targeted monotherapies or combinations exist for RCC, largely due to insufficient preclinical validation in patient-derived models [e.g., patient-derived xenografts (PDXs) and organoids]. Existing studies have relied heavily on established cell lines and conventional mouse models, which fail to recapitulate RCC’s genetic diversity and TME complexity; this raises concerns about translatability. The long-term safety of ferroptosis inducers (e.g., potential off-target effects on normal renal tissue or promotion of secondary tumors) also remains unevaluated in prolonged preclinical studies. 4) Underexplored therapeutic strategies and resistance mechanisms: While combination therapies [e.g., everolimus + erastin/RSL3 (260) and URB597 + RSL3 (264)] show promise, rational combination design remains limited by incomplete mechanistic understanding. For example, the synergy between ferroptosis inducers and immune checkpoint inhibitors (PD-1/PD-L1 blockers)—a cornerstone of RCC therapy—has not been explored. It is unknown whether ferroptosis-induced immunogenic cell death (ICD) enhances anti-tumor immunity or if immune checkpoint inhibition reverses ferroptosis resistance via TME remodeling. Additionally, natural compounds [e.g., icariin II (234) and lycorine (241)] lack clear molecular targets: their direct binding partners or upstream signaling pathways (e.g., epigenetic regulators and kinase cascades) that modulate ferroptosis are poorly defined, hindering the optimization of potency and selectivity. Drug resistance mechanisms beyond DPP9-NRF2 (255) and AIM2-FOXO3a (258) are also underexplored—whether epigenetic modifications (e.g., DNA methylation of ACSL4), post-translational regulation (e.g., GPX4 ubiquitination), or metabolic rewiring (e.g., alternative antioxidant pathways) drives acquired resistance to ferroptosis inducers requires investigation. Finally, the tumor selectivity of ferroptosis inducers—why they spare normal kidney tissue (234, 245)—lacks a molecular explanation; understanding this could guide the development of more selective agents with reduced toxicity. 5) Gaps in ferroptosis and RCC metastasis: Ferroptosis is proposed to contribute to RCC metastasis (232), but the underlying mechanisms are largely unexplored. For example, whether ferroptosis resistance promotes RCC cell dissemination, or if metastatic lesions exhibit distinct ferroptosis vulnerabilities compared to primary tumors, remains unknown. The role of ferroptosis in the “metastatic niche” (e.g., bone and lung metastases of RCC) is also unaddressed—whether the niche’s microenvironment (e.g., hypoxia and iron availability) modulates ferroptosis to support metastatic outgrowth requires clarification. This gap is critical, as metastatic RCC remains incurable with current therapies, and ferroptosis targeting could offer a new avenue to treat advanced disease.

These gaps highlight the need for future research to move beyond descriptive mechanisms toward subtype-specific, genotype-driven, and clinically focused investigations. Addressing these areas will not only deepen the understanding of ferroptosis in RCC but also accelerate the translation of ferroptosis-targeted strategies into effective, personalized therapies for this lethal disease.

### 3.7 Ferroptosis and other kidney diseases

#### 3.7.1 Ferroptosis and contrast-induced nephropathy

Contrast-induced nephropathy (CIN) is a common complication associated with the use of iodinated contrast agents. Currently, preventive measures for CIN primarily involve hydration and volume expansion, but there is no ideal treatment available. In recent years, studies have shown that the administration of ferroptosis inhibitors in animal models of CIN can improve renal pathological damage and restore kidney function (265, 266). In *in vitro* models, ferroptosis inhibitors such as Lip-1 and DFO have demonstrated protective effects on iodinated contrast agent-stimulated HK-2 cells. Additionally, hemin (a porphyria therapeutic agent) has been shown to upregulate GPX4 via the HO-1/NRF2 signaling pathway, thereby inhibiting oxidative stress and ferroptosis, which alleviates CIN (265). Caloric restriction has also been reported to ameliorate CIN by activating the sirtuin 1/GPX4 signaling pathway (266). The discovery of ferroptosis provides new insights into the prevention and treatment of CIN; however, the current evidence is limited, and more high-quality studies are needed for further validation.

Current studies have confirmed ferroptosis involvement in CIN but fail to clarify the core initiating events by which iodinated contrast agents trigger ferroptosis. For instance, it remains undefined whether contrast agents primarily induce iron overload, disrupt the System Xc^−^–GSH–GPX4 axis, or directly promote lipid peroxidation, as well as which of these pathways is the rate-limiting step in CIN. The upstream signaling linking contrast agent exposure to HO-1/NRF2 or sirtuin 1/GPX4 activation (265, 266) is also poorly elucidated; whether contrast-induced oxidative stress acts as a direct trigger, or if other intermediates (e.g., endoplasmic reticulum stress and mitochondrial dysfunction) are involved, requires definitive validation. Additionally, the role of non-classical ferroptosis regulators (e.g., FSP1, DHODH) in CIN is entirely unexplored—whether these pathways compensate for GPX4 dysfunction in CIN or if their dysregulation exacerbates susceptibility remains unknown, limiting the development of multi-targeted interventions. Most preclinical studies have relied on HK-2 cells (human proximal tubular epithelial cells) and generic animal models, but the cell type-specific contribution to CIN-related ferroptosis is severely understudied. For example, renal endothelial cells (critical for maintaining microvascular integrity) or mesangial cells may also undergo ferroptosis in response to contrast agents, yet their role in amplifying CIN injury (e.g., via paracrine signaling or microvascular rarefaction) has not been rigorously characterized. Furthermore, the impact of clinical contexts (e.g., different types of iodinated contrast agents and varying injection doses) on ferroptosis induction remains unexplored—whether high-osmolar *vs*. iso-osmolar contrast agents differ in their ability to trigger ferroptosis, or if dose-dependent thresholds exist, is unclear. The influence of patient comorbidities (e.g., pre-existing CKD, diabetes, and hypertension)—key risk factors for CIN—is also underexplored; it is unknown whether these conditions exacerbate CIN-related ferroptosis by pre-disrupting iron homeostasis or antioxidant capacity, which limits the development of risk-stratified therapies. A major translational barrier is the absence of CIN-specific, non-invasive ferroptosis biomarkers with clinical utility. Current markers (e.g., GPX4 expression, serum creatinine, and lipid peroxides) lack specificity for ferroptosis-driven CIN, as they are altered in other acute kidney injuries or metabolic disorders. Validating urine-based biomarkers (e.g., exosomal ferroptosis-related proteins and 4-HNE metabolites) or circulating ferroptosis signatures that predict CIN risk, severity, or response to interventions is urgently needed to guide clinical decision-making. Furthermore, all ferroptosis-targeted strategies for CIN remain in preclinical stages; no clinical trials have evaluated the efficacy and safety of ferroptosis inhibitors (e.g., Lip-1 and DFO) or activators of protective pathways (e.g., hemin) in humans. Critical questions about clinical application remain unaddressed: What is the optimal administration window (pre- *vs*. post-contrast exposure) for ferroptosis modulators? How should doses be adjusted for high-risk patients (e.g., those with pre-existing renal impairment)? Do ferroptosis modulators synergize with standard preventive measures (e.g., hydration) or pose risks of off-target effects? These gaps highlight the need for future research to move beyond descriptive associations toward systematic dissection of ferroptosis initiation mechanisms, cell type-specific contributions, and clinical validation. Addressing these areas will not only deepen the understanding of ferroptosis in CIN but also accelerate the translation of targeted strategies into effective preventive or therapeutic options for this common and clinically impactful complication.

#### 3.7.2 Ferroptosis and autosomal dominant polycystic kidney disease

Autosomal dominant polycystic kidney disease (ADPKD) is the most common hereditary kidney disease, caused by mutations in either the PKD1 or PKD2 gene, and is characterized by the development of multiple renal cysts. Over time, these cysts enlarge, leading to ESRD (267). The formation and progression of cysts in ADPKD are also associated with oxidative stress, inflammation, and cell death (268, 269). In ADPKD patients, the expression levels of GPX4, SLC3A2, and SLC7A11 are significantly downregulated in the kidneys compared to normal kidneys, while the expression of TFR1 and divalent metal transporter 1 is markedly upregulated. Furthermore, kidneys from PKD1-knockout mice exhibit prominent features of ferroptosis, including the downregulation of SLC3A2, SLC7A11, and GPX4 and the upregulation of TFR1, divalent metal transporter 1, and HO-1, as well as mitochondrial abnormalities such as reduced mitochondrial cristae and ruptured mitochondrial outer membranes. Treatment with ferroptosis inducers exacerbates these changes and promotes cyst growth, whereas the administration of ferroptosis inhibitors significantly alleviates these alterations. *In vitro* studies have further confirmed that the lipid peroxidation product 4-HNE promotes the proliferation of PKD1-mutant cells and cyst growth via serine/threonine protein kinase, ribosomal protein S6, signal transducer and activator of transcription 3, and retinoblastoma tumor suppressor pathways (270). These findings suggest that ferroptosis is closely related to the development and progression of ADPKD, and exploring its mechanisms may offer new therapeutic hope for ADPKD. It has been reported that the expression and activity of antioxidant enzymes [such as glutathione peroxidase (GPX) and SOD] are reduced in two different PKD animal models (271). Additionally, in mouse embryonic kidney organ cultures, the lipid peroxidation compound tBHP increases lipid peroxidation in human polycystic kidneys and promotes cyst growth (272). Recently, we found that inhibiting ferroptosis with Fer-1 delays cyst growth in both rapidly progressive and slowly progressive ADPKD mouse models, while inducing ferroptosis with its inducer erastin promotes cyst growth in these mouse models (270). Taken together, ferroptosis is one of the key mechanisms driving cyst progression in ADPKD, and targeting ferroptosis may represent a novel therapeutic strategy for treating ADPKD.

While compelling evidence establishes ferroptosis as a key driver of cyst progression in ADPKD—linked to PKD1/PKD2 mutations, disrupted antioxidant networks (GPX4/SLC7A11), iron overload, and lipid peroxidation—and validates ferroptosis modulators as potential therapeutics, several critical, insufficiently addressed research gaps persist. These gaps limit a comprehensive understanding of ferroptosis-driven ADPKD pathogenesis and hinder translational progress, necessitating critical dissection. 1) Mechanistic gaps in PKD mutation–ferroptosis link and regulatory networks: Current studies have confirmed ferroptosis-related pathway dysregulation in ADPKD but have failed to clarify the direct molecular link between PKD1/PKD2 mutations and ferroptosis initiation. For instance, it remains undefined whether PKD1/PKD2 loss-of-function directly modulates the transcription or stability of ferroptosis regulators (e.g., SLC7A11, GPX4, and TFR1) or indirectly via downstream signaling cascades (e.g., cAMP/PKA and mTOR, which are known to be dysregulated in ADPKD). The upstream mechanisms by which PKD mutations induce mitochondrial abnormalities (e.g., cristae reduction and outer membrane rupture) and their specific contribution to ferroptosis (e.g., mitochondrial ROS production *vs*. iron dyshomeostasis) also require definitive validation. Additionally, the role of non-classical ferroptosis suppressors (e.g., FSP1, DHODH, and GCH1/BH4) in ADPKD is entirely unexplored—whether these pathways compensate for GPX4 downregulation in cystic kidneys or if their dysfunction exacerbates ferroptosis susceptibility remains unknown, limiting the development of multi-targeted interventions. Furthermore, while 4-HNE promotes cyst growth via multiple kinase pathways (270), the hierarchical integration of these pathways (e.g., which is the rate-limiting step) and their crosstalk with ferroptosis core networks (e.g., lipid peroxidation amplification) are poorly defined. 2) Cell type- and phenotype-specific regulatory blank spots: Most studies have focused on global renal changes or generic cystic cells, but the cell type-specific contribution to ADPKD-related ferroptosis is severely understudied. For example, cyst-lining epithelial cells (the primary drivers of ADPKD progression) may exhibit distinct ferroptosis vulnerabilities compared to adjacent normal tubular epithelial cells, yet the molecular basis for this difference (e.g., unique lipid metabolic profiles and antioxidant capacity) has not been rigorously characterized. Renal interstitial cells, endothelial cells, or immune cells in ADPKD kidneys may also undergo ferroptosis or modulate cyst cell ferroptosis via paracrine signaling—their roles in amplifying cyst growth or fibrosis remain unaddressed. Additionally, ADPKD exhibits significant phenotypic heterogeneity [rapidly *vs*. slowly progressive disease (270)], but ferroptosis mechanisms across these phenotypes are unexplored. It is unknown whether rapidly progressive ADPKD is driven by more severe ferroptosis or distinct regulatory pathways, which limits the development of phenotype-tailored therapies. 3) Limitations in clinical translation and biomarker development: A major translational barrier is the absence of ADPKD-specific, non-invasive ferroptosis biomarkers with clinical utility. Current markers (e.g., GPX4 expression, 4-HNE, and serum creatinine) lack specificity for ferroptosis-driven cyst progression, as they are altered in other kidney diseases or metabolic disorders. Validating urine-based biomarkers (e.g., cyst-derived exosomal ferroptosis regulators and lipid peroxide metabolites) or circulating signatures that correlate with ADPKD disease stage, cyst growth rate, or response to ferroptosis modulators is urgently needed to guide clinical trial design and patient stratification. Furthermore, all ferroptosis-targeted strategies for ADPKD remain in preclinical stages—no clinical trials have evaluated the efficacy and safety of ferroptosis inhibitors (e.g., Fer-1) or inducers in humans. Critical clinical questions remain unaddressed: What is the optimal administration window (early *vs*. advanced ADPKD)? How should doses be adjusted for patients with comorbidities (e.g., ADPKD with pre-existing CKD and hypertension)? Do ferroptosis modulators interact with existing ADPKD therapies (e.g., tolvaptan, a vasopressin V2 receptor antagonist)? 4) Underexplored therapeutic strategies and safety concerns: Preclinical studies have relied on short-term administration of ferroptosis modulators, but the long-term safety and efficacy of these agents in ADPKD are unknown. For example, prolonged ferroptosis inhibition may promote tumorigenesis (by suppressing cell death in pre-malignant cells) or exacerbate other pathological processes (e.g., fibrosis) in ADPKD kidneys, risks that require rigorous evaluation in long-term animal models. Natural compounds or TCM formulas with ferroptosis-inhibitory effects in other kidney diseases (e.g., berberine and paeoniflorin) have not been tested in ADPKD, representing an untapped therapeutic avenue. Additionally, the potential for synergistic combination therapies (e.g., ferroptosis inhibitors + tolvaptan or ferroptosis inhibitors + mTOR inhibitors) is understudied; whether combining these agents enhances cyst growth suppression while reducing individual drug toxicity remains untested. Finally, the impact of ADPKD-related systemic abnormalities (e.g., electrolyte disturbances, hypertension, and liver cysts) on ferroptosis pathways and therapeutic responses is unexplored, which may affect the translatability of preclinical findings. These gaps highlight the need for future research to move beyond descriptive associations toward systematic dissection of PKD mutation–ferroptosis crosstalk, cell type-specific functions, and clinical validation. Addressing these areas will not only deepen the understanding of ferroptosis in ADPKD but also accelerate the translation of targeted strategies into effective, safe therapies for this common hereditary kidney disease.

#### 3.7.3 Ferroptosis and IgA nephropathy

IgA nephropathy is the most common primary glomerular disease. Recent studies have found that GPX4 expression in the kidneys of patients with IgA nephropathy is significantly lower than in healthy controls. In an *in vitro* model (human mesangial cells stimulated by Gd-IgA1), cell viability was markedly reduced, accompanied by lipid peroxidation and characteristic mitochondrial changes associated with ferroptosis. However, treatment with ferroptosis inhibitors alleviated these changes and improved renal injury (273). Mechanistically, the downregulation of peroxisome proliferator-activated receptor α mediates the regulation of fatty acid-binding protein 1 on GPX4 and ACSL4, leading to ferroptosis and promoting the development of IgA nephropathy (273). Therefore, ferroptosis may be an important contributing factor and a potential therapeutic target in IgA nephropathy. While preliminary evidence identifies ferroptosis as a key pathogenic factor in IgA nephropathy—linked to GPX4 downregulation, Gd-IgA1-induced mesangial cell injury, and the PPARα–FABP1–ACSL4 regulatory axis—and validates ferroptosis inhibitors as potential therapeutics, several critical, insufficiently addressed research gaps persist. These gaps limit a comprehensive understanding of ferroptosis-driven IgA nephropathy progression and hinder translational progress, necessitating critical dissection to enhance the review’s depth and clinical relevance. 1) Mechanistic gaps in pathway initiation and crosstalk: Current studies have clarified the PPARα–FABP1-mediated regulation of GPX4/ACSL4 (273) but have failed to elucidate the upstream triggers linking Gd-IgA1 deposition to ferroptosis initiation. For instance, it remains undefined whether Gd-IgA1 directly binds to mesangial cell receptors to activate the PPARα–FABP1 axis, or if indirect signals (e.g., inflammatory cytokines and complement activation—hallmarks of IgA nephropathy) are required. The specific molecular mechanism by which FABP1 modulates GPX4 and ACSL4 is also vague; whether it acts via transcriptional regulation, post-translational modification, or lipid metabolism rewiring requires definitive validation. Additionally, the role of non-classical ferroptosis regulators (e.g., FSP1, DHODH, and GCH1/BH4) in IgA nephropathy is entirely unexplored; whether these radical-trapping antioxidant pathways compensate for GPX4 downregulation in affected kidneys or if their dysfunction exacerbates ferroptosis susceptibility remains unknown, limiting multi-targeted therapeutic design. Furthermore, the crosstalk between ferroptosis and other IgA nephropathy-related pathways (e.g., mesangial cell proliferation and extracellular matrix deposition) is fragmented; how ferroptosis-induced mesangial cell injury amplifies glomerular pathology beyond cell death has not been systematically characterized. 2) Cell type- and clinical phenotype-specific regulatory blank spots: Most research focuses on human mesangial cells, but the cell type-specific contribution to IgA nephropathy-related ferroptosis is severely understudied. For example, glomerular endothelial cells, podocytes, or tubular epithelial cells—all involved in IgA nephropathy pathogenesis—may also undergo ferroptosis or modulate mesangial cell ferroptosis via paracrine signaling, yet their roles in amplifying renal injury remain unaddressed. Immune cells (e.g., IgA1-producing B cells and infiltrating macrophages) are central to IgA nephropathy, but whether their ferroptosis contributes to autoimmunity or inflammation in the disease is unexplored. Additionally, IgA nephropathy exhibits diverse clinical phenotypes (e.g., subclinical *vs*. rapidly progressive, and focal proliferative *vs*. diffuse proliferative lesions), but ferroptosis mechanisms across these phenotypes are unexplored. It is unknown whether severe or progressive IgA nephropathy is associated with more intense ferroptosis or distinct regulatory pathways, which limits the development of phenotype-tailored therapies. The impact of disease stages (early *vs*. advanced) on ferroptosis activity—whether ferroptosis is a driver of early injury or a secondary consequence of chronic damage—also remains unclear. 3) Limitations in clinical translation and biomarker development: A major translational barrier is the absence of IgA nephropathy-specific, non-invasive ferroptosis biomarkers with clinical utility. Current markers (e.g., GPX4 expression, lipid peroxides, and serum creatinine) lack specificity for ferroptosis-driven disease, as they are altered in other glomerular diseases or metabolic disorders. Validating urine-based biomarkers (e.g., glomerular cell-derived exosomal ferroptosis regulators, and 4-HNE metabolites) or circulating signatures that correlate with IgA nephropathy disease activity, pathological severity, or response to interventions is urgently needed to guide clinical trial design and patient stratification. Furthermore, all ferroptosis-targeted strategies remain in preclinical stages—no clinical trials have evaluated the efficacy and safety of ferroptosis inhibitors in IgA nephropathy patients. Critical clinical questions remain unaddressed: What is the optimal administration window for ferroptosis modulators (early intervention to prevent progression *vs*. late-stage to mitigate damage)? How should doses be adjusted for patients with comorbidities (e.g., IgA nephropathy with hypertension or diabetes)? Do ferroptosis inhibitors synergize with standard therapies (e.g., glucocorticoids and immunosuppressants) or pose risks of off-target effects? 4) Underexplored therapeutic strategies and model limitations: Preclinical studies have relied on *in vitro* mesangial cell models and lack validated animal models that fully recapitulate human IgA nephropathy’s pathological features (e.g., glomerular IgA1 deposition and mesangial proliferation). The translatability of ferroptosis-targeted therapies from current models to humans is thus uncertain. Natural compounds or TCM formulas with ferroptosis-inhibitory effects in other kidney diseases (e.g., berberine and paeoniflorin) have not been tested in IgA nephropathy, representing an untapped therapeutic avenue. Additionally, the long-term safety of ferroptosis inhibitors in IgA nephropathy is unevaluated—prolonged inhibition may promote tumorigenesis or exacerbate fibrosis, risks that require rigorous assessment in long-term preclinical models. The potential for combination therapies (e.g., ferroptosis inhibitors + IgA-lowering agents) is also understudied, as is the impact of genetic variability (e.g., polymorphisms in ferroptosis regulators) on therapeutic responses. These gaps highlight the need for future research to move beyond descriptive associations toward systematic dissection of Gd-IgA1–ferroptosis crosstalk, cell type-specific functions, and clinical validation. Addressing these areas will not only deepen the understanding of ferroptosis in IgA nephropathy but also accelerate the translation of targeted strategies into effective therapies for this common primary glomerular disease.

#### 3.7.4 Ferroptosis and kidney stones

Kidney stones affect approximately 10% of the global population, with a 5-year recurrence rate as high as 50%, and are a risk factor for ESRD (274). High concentrations of oxalate can cause renal tubular epithelial cell injury and kidney stone formation. In oxalate-stimulated HK-2 cells, iron levels increase, accompanied by the upregulation of ACSL4 and TFR1 expression and the downregulation of GPX4, SLC7A11, and ferritin light chain expression, along with typical ferroptosis-related mitochondrial alterations (275). These findings suggest that oxalate can induce ferroptosis in HK-2 cells. However, the specific mechanisms by which ferroptosis contributes to kidney stone formation remain unclear, and the therapeutic potential of targeting ferroptosis for kidney stone treatment requires further validation. However, the specific mechanisms by which ferroptosis contributes to kidney stone formation remain unclear, and the therapeutic potential of targeting ferroptosis for kidney stone treatment requires further validation. Beyond these preliminary observations, critical knowledge gaps and unaddressed questions hinder a comprehensive understanding of the ferroptosis–kidney stone axis and its translational value. First, the molecular trigger specificity of oxalate-induced ferroptosis in renal tubular cells is undefined—how oxalate precisely initiates the synergistic dysregulation of iron uptake (TFR1), lipid metabolism (ACSL4), and antioxidant defense (GPX4/SLC7A11) remains elusive, with no clarity on upstream signaling molecules or post-translational modifications that link oxalate exposure to ferroptosis machinery. Second, cell-type and stone-subtype heterogeneity is largely ignored: current studies have focused solely on HK-2 cells, while the role of ferroptosis in other renal resident cells (e.g., renal interstitial cells and collecting duct cells) or infiltrating immune cells during stone formation is unknown. Additionally, whether ferroptosis contributes differently to distinct kidney stone types (e.g., calcium oxalate *vs*. calcium phosphate stones) or disease stages (initial crystallization *vs*. stone growth/recurrence) has not been explored. Third, the causal relationship between ferroptosis and stone pathogenesis lacks rigorous validation—existing data only confirm oxalate induces ferroptosis, but it remains unclear whether ferroptosis directly promotes crystal adhesion, tubular injury, or inflammatory responses (key steps in stone formation) or is merely a secondary consequence of oxalate toxicity. Fourth, clinical translational potential is underdeveloped: there is a lack of clinical cohort data to confirm whether ferroptosis biomarkers (e.g., GPX4 downregulation and lipid peroxide accumulation) are elevated in kidney stone patients, and preclinical studies have not addressed whether targeting ferroptosis can reduce stone formation or recurrence (rather than just protecting tubular cells *in vitro*). Finally, the crosstalk between ferroptosis and other stone-related pathways (e.g., oxidative stress, inflammation, and autophagy) is unexplored—whether ferroptosis acts independently or interacts with these pathways to exacerbate stone pathogenesis requires systematic dissection. Addressing these gaps will be essential to clarify the functional relevance of ferroptosis in kidney stone disease and unlock its potential as a therapeutic target.

### 3.8 Expression distribution of representative ferroptosis-promoting and ferroptosis-suppressing signature genes in renal cells of healthy individuals and disease states

**Figure 6:**
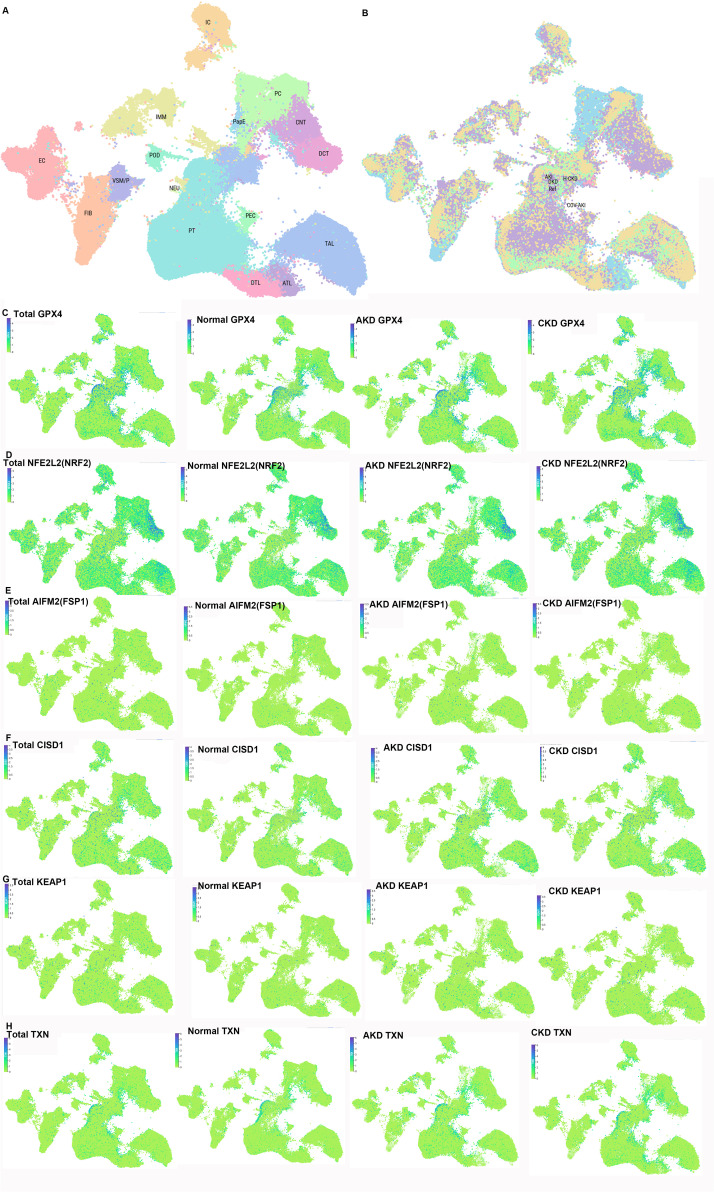
Expression distribution of representative ferroptosis-suppressing signature genes in renal cells under healthy conditions and disease states [acute kidney injury (AKI) and chronic kidney disease (CKD)] as exemplified by the GSE183279 dataset. **(A)** UMAP clustering analysis of renal cell types demonstrating distinct cellular subpopulations including endothelial cells (EC), fibroblasts (FIB), vascular smooth muscle cells/pericytes (VSM/P), immune cells (IMM), podocytes (POD), neutrophils (NEU), proximal tubule (PT) cells, parietal epithelial cells (PEC), interstitial cells (IC), papillary epithelial cells (PapE), principal cells of collecting ducts (PC), connecting tubule cells (CNT), distal convoluted tubule cells (DCT), descending thin limbs (DTL), and thick ascending limbs of Henle’s loop (ATL and TAL). **(B)** UMAP clustering of renal cells under disease conditions (acute kidney injury and chronic kidney disease) illustrating the distribution of disease-associated cellular subpopulations and annotated pathological cell clusters. **(C)** UMAP distribution of *GPX4* expression levels across total cells, normal conditions, AKI, and CKD, revealing differential expression patterns of this core ferroptosis-suppressing gene under distinct renal physiological and pathological states. **(D)** UMAP distribution of nuclear factor erythroid 2-related factor 2 (*NFE2L2/NRF2*) expression levels in total cells, normal conditions, AKI, and CKD, reflecting its expression profiles during renal disease progression. **(E)** UMAP distribution of apoptosis-inducing factor mitochondria-associated 2 (*AIFM2/FSP1*) expression levels in total cells, normal conditions, AKI, and CKD, demonstrating expression heterogeneity of this ferroptosis-inhibitory gene. **(F)** UMAP distribution of CDGSH iron sulfur domain-containing protein 1 (*CISD1*) expression levels in total cells, normal conditions, AKI, and CKD, revealing its expression patterns within the renal pathological microenvironment. **(G)** UMAP distribution of Kelch-like ECH-associated protein 1 (*KEAP1*) expression levels in total cells, normal conditions, AKI, and CKD, illustrating its regulatory expression characteristics in renal disease states. **(H)** UMAP distribution of thioredoxin (*TXN*) expression levels in total cells, normal conditions, AKI, and CKD, displaying differential expression patterns across distinct renal pathological conditions.

In mechanistic investigations of ferroptosis-driven kidney diseases, the GSE183279 single-cell dataset provides critical insights into the cell type-specific expression and disease-dependent regulation of ferroptosis-promoting signature genes. As illustrated in **Figure 6**, Uniform Manifold Approximation and Projection (UMAP) clustering enables the clear identification of the complex renal cellular landscape, encompassing core cellular populations including endothelial cells (EC), fibroblasts (FIB), vascular smooth muscle cells/pericytes (VSM/P), immune cells (IMM), podocytes (POD), neutrophils (NEU), proximal tubule cells (PT), parietal epithelial cells (PEC), interstitial cells (IC), papillary epithelial cells (PapE), principal cells of collecting ducts (PC), connecting tubule cells (CNT), distal tubule cells (DCT), descending thin limbs (DTL), and thick ascending limbs of Henle’s loop (ATL and TAL) (**Figure 6A**). This delineates the intrinsic cellular expression context of ferroptosis-promoting genes. Furthermore, t-distributed Stochastic Neighbor Embedding (t-SNE) clustering under disease conditions (**Figure 6B**) reveals pathological remodeling of renal cellular subpopulations in acute kidney disease (AKD) and CKD, establishing a foundation for exploring the cell-selective roles of ferroptosis during renal injury progression. Analysis of core molecular expression profiles within ferroptosis-promoting pathways reveals significant differences in the expression patterns of ferritin heavy chain 1 (FTH1; **Figure 6C**) and ferritin light chain (FTL; **Figure 6D**)—central regulators of cellular iron metabolism—between AKD, CKD, and normal kidneys. This suggests a tight association between iron metabolism dysregulation and ferroptosis initiation within the renal injury microenvironment. Acyl-CoA synthetase long-chain family member 4 (ACSL4; **Figure 6E**), a key driver enzyme of ferroptotic lipid peroxidation, exhibits heterogeneous expression across different renal cell types and disease states, directly reflecting cell type-specific roles of lipid metabolism reprogramming in renal ferroptosis. Acyl-CoA synthetase long-chain family member 1 (ACSL1; **Figure 6F**), heme oxygenase-1 (HMOX1; **Figure 6G**), and nuclear receptor coactivator 4 (NCOA4; **Figure 6H**) collectively reveal differential activation patterns of ferroptosis-promoting pathways in AKD and CKD renal cells from multiple dimensions—lipid biosynthesis, heme degradation (oxidative stress amplification), and ferritinophagy regulation, respectively. AKD appears characterized by acute oxidative stress and explosive lipid peroxidation, whereas CKD tends toward chronic progression involving iron metabolism dysregulation and ferroptosis. These single-cell resolution expression profiles systematically elucidate the spatiotemporal regulation and cell-selective activation patterns of ferroptosis-promoting signature genes within the renal disease microenvironment. This provides direct cellular and molecular evidence for understanding the mechanisms of ferroptosis imbalance in AKD and CKD pathogenesis while offering crucial theoretical and data support for subsequent intervention strategies targeting ferroptosis-promoting pathways in kidney diseases (e.g., cell type-specific iron metabolism modulation and lipid peroxidation inhibition).

**Figure 7:**
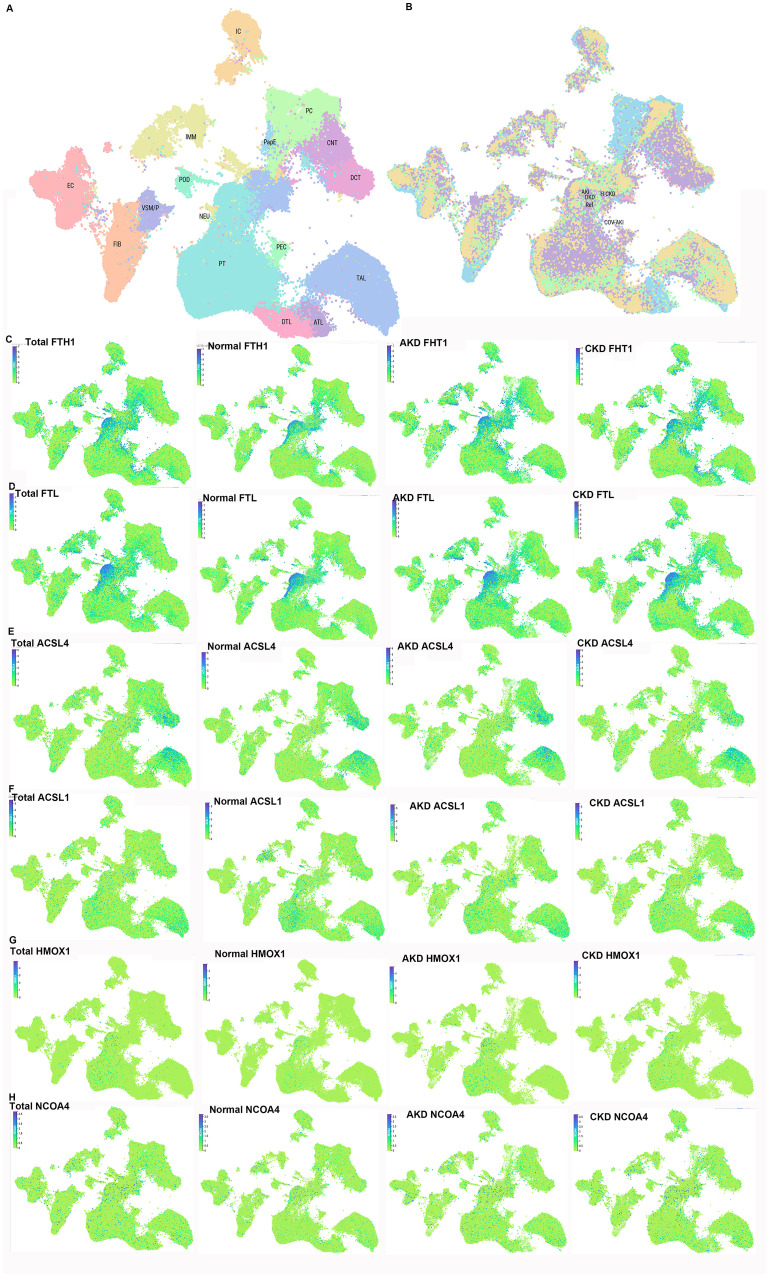
Expression distribution of representative ferroptosis-promoting signature genes in renal cells under healthy conditions and disease states [acute kidney injury (AKI) and chronic kidney disease (CKD)] as exemplified by the GSE183279 dataset. **(A)** UMAP clustering analysis of renal cell types demonstrating distinct subpopulations including endothelial cells (EC), fibroblasts (FIB), vascular smooth muscle cells/pericytes (VSM/P), immune cells (IMM), podocytes (POD), neutrophils (NEU), proximal tubule (PT) cells, parietal epithelial cells (PEC), interstitial cells (IC), papillary epithelial cells (PapE), principal cells of collecting ducts (PC), connecting tubule cells (CNT), distal convoluted tubule cells (DCT), descending thin limbs (DTL), and thick ascending limbs of Henle’s loop (ATL and TAL). **(B)** t-SNE clustering of renal cells under disease conditions (acute kidney injury and chronic kidney disease), illustrating the distribution of disease-associated cellular subpopulations and annotated cell clusters. **(C)** t-SNE distribution of ferritin heavy chain 1 (FTH1) expression levels across total cells, normal conditions, acute kidney injury (AKI), and chronic kidney disease (CKD), revealing differential expression patterns under distinct renal physiological and pathological states. **(D)** t-SNE distribution of ferritin light chain (FTL) expression levels in total cells, normal conditions, AKI, and CKD, reflecting expression profiles across various renal pathological conditions. **(E)** t-SNE distribution of acyl-CoA synthetase long-chain family member 4 (ACSL4) expression levels in total cells, normal conditions, AKI, and CKD, demonstrating expression heterogeneity of this key ferroptosis-related gene. **(F)** t-SNE distribution of acyl-CoA synthetase long-chain family member 1 (ACSL1) expression levels in total cells, normal conditions, AKI, and CKD, revealing expression dynamics during renal disease progression. **(G)** t-SNE distribution of heme oxygenase-1 (HMOX1) expression levels in total cells, normal conditions, AKI, and CKD, illustrating expression patterns within the renal pathological microenvironment. **(H)** t-SNE distribution of nuclear receptor coactivator 4 (NCOA4) expression levels in total cells, normal conditions, AKI, and CKD, displaying differential expression across distinct renal disease states.

In ferroptosis regulation and kidney disease mechanism studies, the GSE183279 dataset provides single-cell resolution insights into the expression heterogeneity of ferroptosis-suppressing signature genes within renal cells and disease microenvironments. As shown in **Figure 7**, UMAP clustering (**Figure 7A**) clearly identifies multiple cellular populations in healthy kidneys—including endothelial cells (EC), fibroblasts (FIB), vascular smooth muscle cells/pericytes (VSM/P), immune cells (IMM), podocytes (POD), and proximal tubule cells (PT)—establishing the cellular expression context of ferroptosis-related genes. t-SNE clustering under disease conditions (**Figure 7B**) further reveals subpopulation remodeling of renal cells in AKD and CKD, providing a foundation for exploring cell type-specific roles of ferroptosis in renal pathological processes. Regarding the expression distribution of core ferroptosis-suppressing genes, GPX4 (**Figure 7C**), a critical ferroptosis inhibitor, exhibits significantly altered expression patterns in AKD and CKD compared to normal kidneys, suggesting potential remodeling of its ferroptosis-suppressive function during renal injury. Nuclear factor erythroid 2-related factor 2 (NFE2L2/NRF2; **Figure 7D**), a master transcription factor regulating antioxidant responses and ferroptosis, displays disease state-dependent expression changes reflecting transcriptional adaptive responses of renal cells to ferroptotic stress. Differential expression of apoptosis-inducing factor mitochondria-associated 2 (AIFM2/FSP1; **Figure 7E**), CDGSH iron sulfur domain-containing protein 1 (CISD1; **Figure 7F**), Kelch-like ECH-associated protein 1 (KEAP1; **Figure 7G**), and thioredoxin (TXN; **Figure 7H**) reveals heterogeneous alterations in ferroptosis resistance capacity of renal cells in AKD and CKD across multiple nodes of ferroptosis-suppressing pathways, including iron metabolism and redox homeostasis. These single-cell expression profiles systematically delineate the spatiotemporal regulation patterns of ferroptosis-suppressing signature genes within the renal disease microenvironment, providing direct cellular and molecular evidence for elucidating the mechanisms of ferroptosis imbalance in AKD and CKD pathogenesis, while offering important references for subsequent research on intervention strategies targeting ferroptosis-suppressing pathways in kidney diseases.

## 4 Challenges and perspectives of ferroptosis in kidney diseases

Ferroptosis, an iron-dependent regulated cell death driven by lipid peroxidation, has emerged as a central pathogenic mediator in kidney diseases—an organ uniquely susceptible to redox imbalance due to its high metabolic activity, abundant iron handling, and structural heterogeneity (276, 277). Beyond its established roles in autoimmune disorders, neurodegeneration, and cancer, ferroptosis exhibits distinct organ-specific features in the kidney, where its activation is tightly linked to the disruption of renal iron homeostasis, lipid metabolism, and antioxidant defense systems (278, 279). As summarized earlier, ferroptosis arises from a dysregulated balance between pro-ferroptotic cues (e.g., labile iron accumulation and PUFA enrichment) and protective mechanisms, including the SLC7A11–GSH–GPX4 axis, GCH1–BH4 pathway, FSP1–CoQH2 system, DHODH–CoQH2 cascade, and MBOAT1/2-mediated monounsaturated fatty acid (MUFA) synthesis (280–282). The Nrf2–KEAP1 pathway stands as a master regulator of this balance, transcriptionally activating genes involved in GSH biosynthesis (GCLC, GCLM, GSR, and GSS), iron metabolism (FTL, FTH1, FPN1, and HO-1), and redox defense (SLC7A11, GPX4, and NQO1)—all of which directly modulate ferroptosis susceptibility (283, 284). The expression distribution of representative ferroptosis-promoting and ferroptosis-suppressing signature genes in renal cells of healthy individuals and disease states is presented in **Figures 6** and **7**. Despite these advances, critical gaps remain: non-coding RNAs (ncRNAs) such as long non-coding RNAs (lncRNAs), circular RNAs (circRNAs), and microRNAs (miRNAs) have only been sporadically implicated in this network (e.g., miR-125a targeting SLC7A11 in diabetic nephropathy), and their cell type-specific roles in renal ferroptosis remain largely uncharacterized.

Current research on ferroptosis in kidney diseases has predominantly focused on AKI and CKD, with accumulating evidence linking ferroptosis to interstitial fibrosis, mitochondrial dysfunction, inflammatory cell infiltration, and impaired tubular regeneration, key drivers of CKD progression (285, 286). However, translating these mechanistic insights into clinical benefit is hindered by three interconnected challenges, each of which offers critical directions for future investigation.

### 4.1 Unresolved complexity of molecular regulatory networks and renal heterogeneity

The molecular machinery governing ferroptosis is characterized by extensive crosstalk between GSH metabolism, iron handling, lipid remodeling, and redox signaling—yet most studies have remained confined to the classical GPX4–GSH and iron metabolism axes. This narrow focus overlooks emerging regulators [e.g., the recently identified ferroptosis suppressor protein 2 (FSP2) or the lipid kinase PI3Kγ] and fails to account for the kidney’s inherent structural and functional heterogeneity. 1) Cell type- and nephron segment-specific susceptibility: The kidney is a highly heterogeneous organ, with distinct nephron segments [PT, distal tubule (DT), collecting duct (CD)] and cell types (podocytes, mesangial cells, interstitial fibroblasts, and endothelial cells) exhibiting divergent ferroptosis susceptibility. For example, PT cells—responsible for ~70% of renal iron reabsorption—express high levels of iron transporters (DMT1 and TfR1) and ACSL4 (a key enzyme for PUFA incorporation into phospholipids), making them inherently more prone to ferroptosis than DT cells or podocytes. Recent single-cell RNA sequencing (scRNA-seq) studies (287, 288) have further revealed that in CKD, PT cells downregulate SLC7A11 and GPX4 expression, while interstitial fibroblasts upregulate pro-ferroptotic genes (e.g., ACSL4 and LOX), suggesting cell type-specific ferroptosis programs that drive fibrosis. However, the functional redundancy and compensation of ferroptosis regulatory pathways across these cell types have not been systematically mapped. 2) Dual contextual roles of ferroptosis: Ferroptosis exhibits stage-dependent duality in kidney disease progression. In early AKI, ferroptosis-mediated elimination of irreversibly damaged PT cells may limit oxidative stress propagation and promote tissue repair, which is supported by studies showing that transient ferroptosis induction reduces AKI severity in mouse models (287, 288). Conversely, sustained ferroptosis activation in the post-AKI phase drives tubular atrophy and interstitial fibrosis, transitioning adaptive repair to maladaptive progression. This duality poses a critical challenge: therapeutic interventions must be timed to target pathological ferroptosis without abrogating its protective roles in early injury clearance. Currently, the molecular “switch” that converts ferroptosis from protective to pathogenic remains undefined, requiring longitudinal studies to map temporal changes in ferroptosis regulators.

### 4.2 Lack of specific biomarkers and translational limitations of therapeutics

The clinical translation of ferroptosis-targeted strategies is bottlenecked by two critical barriers: the absence of specific, standardized biomarkers for ferroptosis in renal tissues and biofluids, and the suboptimal pharmacokinetic and safety profiles of current ferroptosis modulators. 1) Biomarker challenges: Classical ferroptosis markers (MDA, 4-HNE, and C11-BODIPY) are non-specific, as they are also generated by other oxidative stress pathways (e.g., ROS from mitochondrial dysfunction). Emerging candidates such as oxidized phospholipids (oxPCs), ferroptosis-related ncRNAs (e.g., lncRNA FENDRR), or cell type-specific proteins (e.g., PT-specific ACSL4 fragments) show promise but lack standardized detection protocols and clinical validation. To address this, future studies must integrate spatial multi-omics technologies—including spatial transcriptomics, mass spectrometry imaging (MSI), and single-cell proteomics—to establish a “spatiomolecular fingerprint” of ferroptosis in human kidney biopsies. This fingerprint should link molecular signatures (e.g., co-expression of ACSL4 and 4-HNE) to histological lesions (e.g., tubular necrosis and fibrosis), enabling precise localization and quantification of ferroptosis in the renal microenvironment. 2) Therapeutic limitations and innovations: Current ferroptosis inhibitors (Fer-1, Lip-1, and DFO) suffer from poor aqueous solubility, short half-lives, and off-target effects (e.g., DFO-induced iron deficiency anemia). Kidney-targeted delivery systems—such as PEGylated lipid nanoparticles (LNPs) modified with renal tubular cell-targeting ligands (e.g., vitamin B12, which binds to cubilin on PT cells)—have shown promise in preclinical studies, increasing renal Fer-1 accumulation by 5–10-fold and reducing systemic toxicity. Additionally, proteolysis targeting chimeras (PROTACs) offer a novel approach to modulate ferroptosis: PROTACs targeting ACSL4 or Nrf2 have been developed to either induce ferroptosis (in renal cell carcinoma) or enhance antioxidant defense (in CKD), respectively, with improved specificity compared to small-molecule inhibitors. However, these agents remain in early preclinical stages, requiring the optimization of tissue penetration and proteasomal degradation efficiency. For chronic diseases like CKD, long-term safety is paramount; future studies must evaluate the risk of ferroptosis inhibition promoting tumorigenesis (e.g., by protecting pre-neoplastic cells) or impairing iron metabolism homeostasis.

### 4.3 Complex crosstalk with other cell death pathways and functional compensation

Ferroptosis does not act in isolation; it engages in extensive signaling crosstalk with apoptosis, necroptosis, pyroptosis, and NETosis, forming a “cell death network” that regulates renal injury and repair. Blocking a single pathway often triggers compensatory activation of alternative death mechanisms, limiting therapeutic efficacy. 1) Mechanisms of crosstalk: Key molecular nodes mediate crosstalk between ferroptosis and other cell death pathways. For example, RIPK3—best known for driving necroptosis—also phosphorylates GPX4, promoting its degradation and enhancing ferroptosis in AKI. Similarly, ferroptosis-induced lipid peroxidation activates the NLRP3 inflammasome, triggering pyroptosis and amplifying renal inflammation. In late-stage CKD, ferroptosis and necroptosis form a positive feedback loop: ferroptosis-derived DAMPs (e.g., HMGB1) activate RIPK3–MLKL signaling, while necroptosis releases labile iron, further fueling ferroptosis. Conditional GPX4 knockout mice exemplify the risks of single-pathway targeting: while GPX4 deletion in PT cells protects against early AKI by eliminating damaged cells, long-term deletion activates the cGAS–STING pathway—likely via release of fragmented mitochondrial DNA—leading to sustained type I interferon signaling and progressive interstitial fibrosis. 2) Toward “death network modulation”: To overcome functional compensation, future therapeutic strategies must shift from “single-target inhibition” to “network modulation”. This includes developing bifunctional molecules (e.g., Fer-1 conjugated to Nec-1, a necroptosis inhibitor) that co-target complementary pathways or epigenetic editing tools (e.g., dCas9-SunTag-KRAB systems) to dynamically suppress the transcription of ferroptosis–necroptosis crosstalk nodes (e.g., RIPK3). Systems biology approaches—integrating multi-omics data (transcriptomics, metabolomics, and phosphoproteomics) with mathematical modeling—are critical to identify “hub molecules” (e.g., Nrf2 and RIPK3) that regulate multiple death pathways. For kidney cancer, the dual role of ferroptosis adds another layer of complexity: while ferroptosis induction kills cancer cells, it may also promote immune suppression via DAMPs (e.g., ATP) that recruit myeloid-derived suppressor cells (MDSCs). Future studies must optimize ferroptosis inducers to enhance anti-tumor immunity—for example, combining ferroptosis modulators with immune checkpoint inhibitors (e.g., PD-1 blockers) to reverse immunosuppression.

### 4.4 Future perspectives: from mechanistic insight to clinical translation

To fully harness the therapeutic potential of ferroptosis in kidney diseases, future research must address the aforementioned challenges through a combination of mechanistic depth, technological innovation, and clinical validation. We propose four priority directions, with a focus on actionable strategies for researchers. 1) Decipher cell type-specific ferroptosis regulatory networks: Use integrated single-cell multi-omics (scRNA-seq, Single-cell Assay for Transposase-Accessible Chromatin with High-throughput Sequencing (scATAC-seq), and single-cell metabolomics) to map ferroptosis-related gene expression, chromatin accessibility, and metabolite profiles across renal cell types in health and disease. Validate key regulators (e.g., cell type-specific lncRNAs and lipid metabolic enzymes) using kidney organoid models, particularly patient-derived organoids (PDOs) that recapitulate disease-specific phenotypes (e.g., diabetic nephropathy and focal segmental glomerulosclerosis). This will identify cell type-selective therapeutic targets to avoid off-target effects. 2) Develop standardized, clinically applicable biomarkers: Establish a multi-tiered biomarker panel combining biofluid-based markers (e.g., plasma oxPCs and urinary ferroptosis-related miRNAs) and tissue-based spatial signatures (e.g., ACSL4/GPX4 expression ratio in PT cells). Validate this panel in prospective clinical cohorts to assess its prognostic value (e.g., predicting AKI-to-CKD transition) and ability to stratify patients for ferroptosis-targeted therapies. 3) Advance next-generation ferroptosis modulators: Prioritize the development of kidney-targeted, stimuli-responsive delivery systems (e.g., ROS-sensitive LNPs and hypoxia-responsive polymers) to enhance tissue specificity and reduce systemic toxicity. Explore novel modalities such as PROTACs (targeting ACSL4 and Nrf2), gene therapies (e.g., AAV-mediated SLC7A11 overexpression in PT cells), and epigenetic modulators (e.g., m^6^A inhibitors that regulate ferroptosis-related gene expression). 4) Integrate multi-disciplinary approaches for clinical translation: Combine artificial intelligence (AI)-driven drug discovery (e.g., machine learning models to predict ferroptosis modulator efficacy using multi-omics data) with network pharmacology to identify synergistic drug combinations (e.g., ferroptosis inhibitors + anti-fibrotic agents). Conduct phase I/II clinical trials with rigorous pharmacokinetic/pharmacodynamic (PK/PD) monitoring, using validated biomarkers to ensure target engagement (e.g., reduced urinary 4-HNE levels) and safety (e.g., no iron deficiency).

Additionally, cross-species comparative studies (e.g., mouse, rat, and non-human primate models) will help refine therapeutic dosing and minimize translational failure. Genetic lineage tracing techniques can further clarify the contribution of ferroptosis to renal cell fate (e.g., tubular cell death *vs*. transdifferentiation into myofibroblasts). Finally, liquid biopsy technologies (e.g., urinary EVs containing ferroptosis markers) offer a non-invasive alternative to kidney biopsies, enabling longitudinal monitoring of treatment response in clinical settings.

The study of ferroptosis in kidney diseases is entering a transformative phase, from defining basic mechanisms to developing precision therapies. By addressing the challenges of organ heterogeneity, biomarker specificity, pathway crosstalk, and translational efficacy, researchers can unlock the full potential of ferroptosis modulation to halt or reverse kidney disease progression. Ultimately, the integration of fundamental science, technological innovation, and clinical collaboration will establish ferroptosis as a cornerstone of personalized kidney disease management.

## References

[1] Smith PA Sarris I Clark K Wiles K Bramham K . Kidney disease and reproductive health. Nat Rev Nephrol. (2025) 21:127–43. doi: 10.1038/s41581-024-00901-6, PMID: 39501029

[2] Francis A Harhay MN Ong ACM Tummalapalli SL Ortiz A Fogo AB . Chronic kidney disease and the global public health agenda: an international consensus. Nat Rev Nephrol. (2024) 20:473–85. doi: 10.1038/s41581-024-00820-6, PMID: 38570631

[3] Kidney Disease: Improving Global Outcomes (KDIGO) CKD Work Group . KDIGO 2024 clinical practice guideline for the evaluation and management of chronic kidney disease. Kidney Int. (2024) 105:S117–314. doi: 10.1016/j.kint.2023.10.018, PMID: 38490803

[4] Awdishu L Maxson R Gratt C Rubenzik T Battistella M . KDIGO 2024 clinical practice guideline on evaluation and management of chronic kidney disease: A primer on what pharmacists need to know. Am J Health Syst Pharm. (2025) 82:660–71. doi: 10.1093/ajhp/zxaf044, PMID: 40197825 PMC12158546

[5] Vivante A . Genetics of chronic kidney disease. N Engl J Med. (2024) 391:627–39. doi: 10.1056/NEJMra2308577, PMID: 39141855

[6] Anders HJ Kitching AR Leung N Romagnani P . Glomerulonephritis: immunopathogenesis and immunotherapy. Nat Rev Immunol. (2023) 23:453–71. doi: 10.1038/s41577-022-00816-y, PMID: 36635359 PMC9838307

[7] Miguel V Shaw IW Kramann R . Metabolism at the crossroads of inflammation and fibrosis in chronic kidney disease. Nat Rev Nephrol. (2025) 21:39–56. doi: 10.1038/s41581-024-00889-z, PMID: 39289568

[8] Romagnani P Agarwal R Chan JCN Levin A Kalyesubula R Karam S . Chronic kidney disease. Nat Rev Dis Primers. (2025) 11:8. doi: 10.1038/s41572-024-00589-9, PMID: 39885176

[9] Yang K Zeng L Zeng J Deng Y Wang S Xu H . Research progress in the molecular mechanism of ferroptosis in Parkinson’s disease and regulation by natural plant products. Ageing Res Rev. (2023) 91:102063. doi: 10.1016/j.arr.2023.102063, PMID: 37673132

[10] Zeng L Yang K Yu G Hao W Zhu X Ge A . Advances in research on immunocyte iron metabolism, ferroptosis, and their regulatory roles in autoimmune and autoinflammatory diseases. Cell Death Dis. (2024) 15:481. doi: 10.1038/s41419-024-06807-2, PMID: 38965216 PMC11224426

[11] Dixon SJ Lemberg KM Lamprecht MR Skouta R Zaitsev EM Gleason CE . Ferroptosis: an iron-dependent form of nonapoptotic cell death. Cell. (2012) 149:1060–72. doi: 10.1016/j.cell.2012.03.042, PMID: 22632970 PMC3367386

[12] Yang WS Stockwell BR . Synthetic lethal screening identifies compounds activating iron-dependent, nonapoptotic cell death in oncogenic-RAS-harboring cancer cells. Chem Biol. (2008) 15:234–45. doi: 10.1016/j.chembiol.2008.02.010, PMID: 18355723 PMC2683762

[13] Yagoda N von Rechenberg M Zaganjor E Bauer AJ Yang WS Fridman DJ . RAS-RAF-MEK-dependent oxidative cell death involving voltage-dependent anion channels. Nature. (2007) 447:864–8. doi: 10.1038/nature05859, PMID: 17568748 PMC3047570

[14] Wang X Fang X Wang F . Pleiotropic actions of iron balance in diabetes mellitus. Rev Endocr Metab Disord. (2015) 16:15–23. doi: 10.1007/s11154-014-9303-y, PMID: 25520048

[15] Cai Z Wu X Song Z Sun S Su Y Wang T . Metformin potentiates nephrotoxicity by promoting NETosis in response to renal ferroptosis. Cell Discov. (2023) 9:104. doi: 10.1038/s41421-023-00595-3, PMID: 37848438 PMC10582023

[16] Bayır H Dixon SJ Tyurina YY Kellum JA Kagan VE . Ferroptotic mechanisms and therapeutic targeting of iron metabolism and lipid peroxidation in the kidney. Nat Rev Nephrol. (2023) 19:315–36. doi: 10.1038/s41581-023-00689-x, PMID: 36922653

[17] Long Z Luo Y Yu M Wang X Zeng L Yang K . Targeting ferroptosis: a new therapeutic opportunity for kidney diseases. Front Immunol. (2024) 15:1435139. doi: 10.3389/fimmu.2024.1435139, PMID: 39021564 PMC11251909

[18] Li J Zheng S Fan Y Tan K . Emerging significance and therapeutic targets of ferroptosis: a potential avenue for human kidney diseases. Cell Death Dis. (2023) 14:628. doi: 10.1038/s41419-023-06144-w, PMID: 37739961 PMC10516929

[19] Ru Q Li Y Chen L Wu Y Min J Wang F . Iron homeostasis and ferroptosis in human diseases: mechanisms and therapeutic prospects. Signal Transduct Target Ther. (2024) 9:271. doi: 10.1038/s41392-024-01969-z, PMID: 39396974 PMC11486532

[20] Wang Y Hu J Wu S Fleishman JS Li Y Xu Y . Targeting epigenetic and posttranslational modifications regulating ferroptosis for the treatment of diseases. Signal Transduct Target Ther. (2023) 8:449. doi: 10.1038/s41392-023-01720-0, PMID: 38072908 PMC10711040

[21] Zeng F Nijiati S Tang L Ye J Zhou Z Chen X . Ferroptosis detection: from approaches to applications. Angew Chem Int Ed Engl. (2023) 62:e202300379. doi: 10.1002/anie.202300379, PMID: 36828775

[22] Tang D Chen X Kang R Kroemer G . Ferroptosis: molecular mechanisms and health implications. Cell Res. (2021) 31:107–25. doi: 10.1038/s41422-020-00441-1, PMID: 33268902 PMC8026611

[23] Galy B Conrad M Muckenthaler M . Mechanisms controlling cellular and systemic iron homeostasis. Nat Rev Mol Cell Biol. (2024) 25:133–55. doi: 10.1038/s41580-023-00648-1, PMID: 37783783

[24] Sawicki KT De Jesus A Ardehali H . Iron metabolism in cardiovascular disease: physiology, mechanisms, and therapeutic targets. Circ Res. (2023) 132:379–96. doi: 10.1161/CIRCRESAHA.122.321667, PMID: 36730380 PMC9907000

[25] Ni S Yuan Y Kuang Y Li X . Iron metabolism and immune regulation. Front Immunol. (2022) 13:816282. doi: 10.3389/fimmu.2022.816282, PMID: 35401569 PMC8983924

[26] ScIndia PhD Y Leeds Md J Swaminathan Md S . Iron homeostasis in healthy kidney and its role in acute kidney injury. Semin Nephrol. (2019) 39:76–84. doi: 10.1016/j.semnephrol.2018.10.006, PMID: 30606409

[27] Fuhrmann DC Brüne B . A graphical journey through iron metabolism, microRNAs, and hypoxia in ferroptosis. Redox Biol. (2022) 54:102365. doi: 10.1016/j.redox.2022.102365, PMID: 35717888 PMC9213245

[28] Kajarabille N Latunde-Dada GO . Programmed cell-death by ferroptosis: antioxidants as mitigators. Int J Mol Sci. (2019) 20:4968. doi: 10.3390/ijms20194968, PMID: 31597407 PMC6801403

[29] Mancias JD Wang X Gygi SP Harper JW Kimmelman AC . Quantitative proteomics identifies NCOA4 as the cargo receptor mediating ferritinophagy. Nature. (2014) 509:105–9. doi: 10.1038/nature13148, PMID: 24695223 PMC4180099

[30] Fang Y Chen X Tan Q Zhou H Xu J Gu Q . Inhibiting ferroptosis through disrupting the NCOA4-FTH1 interaction: A new mechanism of action. ACS Cent Sci. (2021) 7:980–9. doi: 10.1021/acscentsci.0c01592, PMID: 34235259 PMC8227600

[31] Park E Chung SW . ROS-mediated autophagy increases intracellular iron levels and ferroptosis by ferritin and transferrin receptor regulation. Cell Death Dis. (2019) 10:822. doi: 10.1038/s41419-019-2064-5, PMID: 31659150 PMC6817894

[32] Stoyanovsky DA Tyurina YY Shrivastava I Bahar I Tyurin VA Protchenko O . Iron catalysis of lipid peroxidation in ferroptosis: Regulated enzymatic or random free radical reaction? Free Radic Biol Med. (2019) 133:153–61. doi: 10.1016/j.freeradbiomed.2018.09.008, PMID: 30217775 PMC6555767

[33] Chen X Li J Kang R Klionsky DJ Tang D . Ferroptosis: machinery and regulation. Autophagy. (2021) 17:2054–81. doi: 10.1080/15548627.2020.1810918, PMID: 32804006 PMC8496712

[34] Yuan H Li X Zhang X Kang R Tang D . Identification of ACSL4 as a biomarker and contributor of ferroptosis. Biochem Biophys Res Commun. (2016) 478:1338–43. doi: 10.1016/j.bbrc.2016.08.124, PMID: 27565726

[35] Doll S Proneth B Tyurina YY Panzilius E Kobayashi S Ingold I . ACSL4 dictates ferroptosis sensitivity by shaping cellular lipid composition. Nat Chem Biol. (2017) 13:91–8. doi: 10.1038/nchembio.2239, PMID: 27842070 PMC5610546

[36] Kuang F Liu J Tang D Kang R . Oxidative damage and antioxidant defense in ferroptosis. Front Cell Dev Biol. (2020) :586578. doi: 10.3389/fcell.2020.586578, PMID: 33043019 PMC7527737

[37] Koppula P Lei G Zhang Y Yan Y Mao C Kondiparthi L . A targetable CoQ-FSP1 axis drives ferroptosis- and radiation-resistance in KEAP1 inactive lung cancers. Nat Commun. (2022) 13:2206. doi: 10.1038/s41467-022-29905-1, PMID: 35459868 PMC9033817

[38] Mao C Liu X Zhang Y Lei G Yan Y Lee H . DHODH-mediated ferroptosis defence is a targetable vulnerability in cancer. Nature. (2021) 593:586–90. doi: 10.1038/s41586-021-03539-7, PMID: 33981038 PMC8895686

[39] Liu J Kang R Tang D . Signaling pathways and defense mechanisms of ferroptosis. FEBS J. (2022) 289:7038–50. doi: 10.1111/febs.16059, PMID: 34092035

[40] Stockwell BR Jiang X Gu W . Emerging mechanisms and disease relevance of ferroptosis. Trends Cell Biol. (2020) 30:478–90. doi: 10.1016/j.tcb.2020.02.009, PMID: 32413317 PMC7230071

[41] Lin L Li X Zhu S Long Q Hu Y Zhang L . Ferroptosis-related NFE2L2 and NOX4 genes are potential risk prognostic biomarkers and correlated with immunogenic features in glioma. Cell Biochem Biophys. (2023) 81:7–17. doi: 10.1007/s12013-022-01124-x, PMID: 36627482 PMC9925512

[42] Park MW Cha HW Kim J Kim JH Yang H Yoon S . NOX4 promotes ferroptosis of astrocytes by oxidative stress-induced lipid peroxidation via the impairment of mitochondrial metabolism in Alzheimer’s diseases. Redox Biol. (2021) 41:101947. doi: 10.1016/j.redox.2021.101947, PMID: 33774476 PMC8027773

[43] Ursini F Maiorino M . Lipid peroxidation and ferroptosis: The role of GSH and GPx4. Free Radic Biol Med. (2020) 152:175–85. doi: 10.1016/j.freeradbiomed.2020.02.027, PMID: 32165281

[44] Wang L Liu Y Du T Yang H Lei L Guo M . ATF3 promotes erastin-induced ferroptosis by suppressing system Xc. Cell Death Differ. (2020) 27:662–75. doi: 10.1038/s41418-019-0380-z, PMID: 31273299 PMC7206049

[45] Gao M Monian P Quadri N Ramasamy R Jiang X . Glutaminolysis and transferrin regulate ferroptosis. Mol Cell. (2015) 59:298–308. doi: 10.1016/j.molcel.2015.06.011, PMID: 26166707 PMC4506736

[46] Zhang X Hou L Guo Z Wang G Xu J Zheng Z . Lipid peroxidation in osteoarthritis: focusing on 4-hydroxynonenal, malondialdehyde, and ferroptosis. Cell Death Discov. (2023) 9:320. doi: 10.1038/s41420-023-01613-9, PMID: 37644030 PMC10465515

[47] Bock FJ Tait SWG . Mitochondria as multifaceted regulators of cell death. Nat Rev Mol Cell Biol. (2020) 21:85–100. doi: 10.1038/s41580-019-0173-8, PMID: 31636403

[48] Zeng L Yang K Yu G Hao W Zhu X Ge A . Advances in research on immunocyte iron metabolism, ferroptosis, and their regulatory roles in autoimmune and autoinflammatory diseases. Cell Death Dis. (2024) 15:481. doi: 10.1038/s41419-024-06807-2, PMID: 38965216 PMC11224426

[49] Zhang W Hu X Shen Q Xing D . Mitochondria-specific drug release and reactive oxygen species burst induced by polyprodrug nanoreactors can enhance chemotherapy. Nat Commun. (2019) 10:1704. doi: 10.1038/s41467-019-09566-3, PMID: 30979885 PMC6461692

[50] De Stefani D Bononi A Romagnoli A Messina A De Pinto V Pinton P . VDAC1 selectively transfers apoptotic Ca2+ signals to mitochondria. Cell Death Differ. (2012) 19:267–73. doi: 10.1038/cdd.2011.92, PMID: 21720385 PMC3263501

[51] Fuhrmann DC Mondorf A Beifuß J Jung M Brüne B . Hypoxia inhibits ferritinophagy, increases mitochondrial ferritin, and protects from ferroptosis. Redox Biol. (2020) 36:101670. doi: 10.1016/j.redox.2020.101670, PMID: 32810738 PMC7452134

[52] Wang YQ Chang SY Wu Q Gou YJ Jia L Cui YM . The protective role of mitochondrial ferritin on erastin-induced ferroptosis. Front Aging Neurosci. (2016) 8:308. doi: 10.3389/fnagi.2016.00308, PMID: 28066232 PMC5167726

[53] Hassin O Oren M . Drugging p53 in cancer: one protein, many targets. Nat Rev Drug Discov. (2023) 22:127–44. doi: 10.1038/s41573-022-00571-8, PMID: 36216888 PMC9549847

[54] Jiang L Kon N Li T Wang SJ Su T Hibshoosh H . Ferroptosis as a p53-mediated activity during tumour suppression. Nature. (2015) 520:57–62. doi: 10.1038/nature14344, PMID: 25799988 PMC4455927

[55] Hu W Zhang C Wu R Sun Y Levine A Feng Z . Glutaminase 2, a novel p53 target gene regulating energy metabolism and antioxidant function. Proc Natl Acad Sci U S A. (2010) 107:7455–60. doi: 10.1073/pnas.1001006107, PMID: 20378837 PMC2867677

[56] Ou Y Wang SJ Li D Chu B Gu W . Activation of SAT1 engages polyamine metabolism with p53-mediated ferroptotic responses. Proc Natl Acad Sci U S A. (2016) 113:E6806–12. doi: 10.1073/pnas.1607152113, PMID: 27698118 PMC5098629

[57] Xie Y Zhu S Song X Sun X Fan Y Liu J . The tumor suppressor p53 limits ferroptosis by blocking DPP4 activity. Cell Rep. (2017) 20:1692–704. doi: 10.1016/j.celrep.2017.07.055, PMID: 28813679

[58] Tarangelo A Magtanong L Bieging-Rolett KT Li Y Ye J Attardi LD . p53 suppresses metabolic stress-induced ferroptosis in cancer cells. Cell Rep. (2018) 22:569–75. doi: 10.1016/j.celrep.2017.12.077, PMID: 29346757 PMC5791910

[59] Dai E Chen X Linkermann A Jiang X Kang R Kagan VE . A guideline on the molecular ecosystem regulating ferroptosis. Nat Cell Biol. (2024) 26:1447–57. doi: 10.1038/s41556-024-01360-8, PMID: 38424270 PMC11650678

[60] Chen Y Fang ZM Yi X Wei X Jiang DS . The interaction between ferroptosis and inflammatory signaling pathways. Cell Death Dis. (2023) 14:205. doi: 10.1038/s41419-023-05716-0, PMID: 36944609 PMC10030804

[61] Bell HN Stockwell BR Zou W . Ironing out the role of ferroptosis in immunity. Immunity. (2024) 57:941–56. doi: 10.1016/j.immuni.2024.03.019, PMID: 38749397 PMC11101142

[62] Chen X Tsvetkov AS Shen HM Isidoro C Ktistakis NT Linkermann A . International consensus guidelines for the definition, detection, and interpretation of autophagy-dependent ferroptosis. Autophagy. (2024) 20:1213–46. doi: 10.1080/15548627.2024.2319901, PMID: 38442890 PMC11210914

[63] Co HKC Wu CC Lee YC Chen SH . Emergence of large-scale cell death through ferroptotic trigger waves. Nature. (8021) 2024:631. doi: 10.1038/s41586-024-07623-6, PMID: 38987590 PMC11639682

[64] Zhang Y Yang J Ouyang C Meng N . The association between ferroptosis and autophagy in cardiovascular diseases. Cell Biochem Funct. (2024) 42:e3985. doi: 10.1002/cbf.3985, PMID: 38509716

[65] Freitas FP Alborzinia H Dos Santos AF Nepachalovich P Pedrera L Zilka O . 7-Dehydrocholesterol is an endogenous suppressor of ferroptosis. Nature. (2024) 626:401–10. doi: 10.1038/s41586-023-06878-9, PMID: 38297129

[66] Li Y Ran Q Duan Q Jin J Wang Y Yu L . 7-Dehydrocholesterol dictates ferroptosis sensitivity. Nature. (2024) 626:411–8. doi: 10.1038/s41586-023-06983-9, PMID: 38297130 PMC11298758

[67] Zhang DD . Natural inhibitor found for cell death by ferroptosis. Nature. (2024) 626:269–70. doi: 10.1038/d41586-024-00080-1, PMID: 38297047

[68] Yang WS SriRamaratnam R Welsch ME Shimada K Skouta R Viswanathan VS . Regulation of ferroptotic cancer cell death by GPX4. Cell. (2014) 156:317–31. doi: 10.1016/j.cell.2013.12.010, PMID: 24439385 PMC4076414

[69] Bersuker K Hendricks JM Li Z Magtanong L Ford B Tang PH . The CoQ oxidoreductase FSP1 acts parallel to GPX4 to inhibit ferroptosis. Nature. (2019) 575:688–92. doi: 10.1038/s41586-019-1705-2, PMID: 31634900 PMC6883167

[70] Liang D Feng Y Zandkarimi F Wang H Zhang Z Kim J . Ferroptosis surveillance independent of GPX4 and differentially regulated by sex hormones. Cell. (2023) 186:2748–2764.e22. doi: 10.1016/j.cell.2023.05.003, PMID: 37267948 PMC10330611

[71] Chen Y Li Z Zhang H Chen H Hao J Liu H . Mitochondrial metabolism and targeted treatment strategies in ischemic-induced acute kidney injury. Cell Death Discov. (2024) 10:69. doi: 10.1038/s41420-024-01843-5, PMID: 38341438 PMC10858869

[72] Lee C Jang MJ Kim BH Park JY You D Jeong IG . Recovery of renal function after administration of adipose-tissue-derived stromal vascular fraction in rat model of acute kidney injury induced by ischemia/reperfusion injury. Cell Tissue Res. (2017) 368:603–13. doi: 10.1007/s00441-017-2585-0, PMID: 28283911

[73] Chen C Wang D Yu Y Zhao T Min N Wu Y . Legumain promotes tubular ferroptosis by facilitating chaperone-mediated autophagy of GPX4 in AKI. Cell Death Dis. (2021) 12:65. doi: 10.1038/s41419-020-03362-4, PMID: 33431801 PMC7801434

[74] Wang Y Quan F Cao Q Lin Y Yue C Bi R . Quercetin alleviates acute kidney injury by inhibiting ferroptosis. J Adv Res. (2020) 28:231–43. doi: 10.1016/j.jare.2020.07.007, PMID: 33364059 PMC7753233

[75] Dall E Brandstetter H . Structure and function of legumain in health and disease. Biochimie. (2016) 122:126–50. doi: 10.1016/j.biochi.2015.09.022, PMID: 26403494

[76] Huang LL Liao XH Sun H Jiang X Liu Q Zhang L . Augmenter of liver regeneration protects the kidney from ischaemia-reperfusion injury in ferroptosis. J Cell Mol Med. (2019) 23:4153–64. doi: 10.1111/jcmm.14302, PMID: 30993878 PMC6533476

[77] Tao WH Shan XS Zhang JX Liu HY Wang BY Wei X . Dexmedetomidine attenuates ferroptosis-mediated renal ischemia/reperfusion injury and inflammation by inhibiting ACSL4 via α2-AR. Front Pharmacol. (2022) 13:782466. doi: 10.3389/fphar.2022.782466, PMID: 35873574 PMC9307125

[78] Wang Y Zhang M Bi R Su Y Quan F Lin Y . ACSL4 deficiency confers protection against ferroptosis-mediated acute kidney injury. Redox Biol. (2022) 51:102262. doi: 10.1016/j.redox.2022.102262, PMID: 35180475 PMC8857079

[79] Zhao Z Li G Wang Y Li Y Xu H Liu W . Cytoplasmic HMGB1 induces renal tubular ferroptosis after ischemia/reperfusion. Int Immunopharmacol. (2023) 116:109757. doi: 10.1016/j.intimp.2023.109757, PMID: 36731154

[80] Liu Y Zhou L Lv C Liu L Miao S Xu Y . PGE2 pathway mediates oxidative stress-induced ferroptosis in renal tubular epithelial cells. FEBS J. (2023) 290:533–49. doi: 10.1111/febs.16609, PMID: 36031392

[81] Eleftheriadis T Pissas G Filippidis G Liakopoulos V Stefanidis I . Reoxygenation induces reactive oxygen species production and ferroptosis in renal tubular epithelial cells by activating aryl hydrocarbon receptor. Mol Med Rep. (2021) 23:41. doi: 10.3892/mmr.2020.11679, PMID: 33179104 PMC7684866

[82] Zheng J Conrad M . Ferroptosis: when metabolism meets cell death. Physiol Rev. (2025) 105:651–706. doi: 10.1152/physrev.00031.2024, PMID: 39661331

[83] Choi N Whitlock R Klassen J Zappitelli M Arora RC Rigatto C . Early intraoperative iron-binding proteins are associated with acute kidney injury after cardiac surgery. J Thorac Cardiovasc Surg. (2019) 157:287–297.e2. doi: 10.1016/j.jtcvs.2018.06.091, PMID: 30195593

[84] Lin CH Tseng HF Hsieh PC Chiu V Lin TY Lan CC . Nephroprotective role of chrysophanol in hypoxia/reoxygenation-induced renal cell damage via apoptosis, ER stress, and ferroptosis. Biomedicines. (2021) 9:1283. doi: 10.3390/biomedicines9091283, PMID: 34572468 PMC8467645

[85] Su L Jiang X Yang C Zhang J Chen B Li Y . Pannexin 1 mediates ferroptosis that contributes to renal ischemia/reperfusion injury. J Biol Chem. (2019) 294:19395–404. doi: 10.1074/jbc.RA119.010949, PMID: 31694915 PMC6916502

[86] Pan J Zhao J Feng L Xu X He Z Liang W . Inhibition of USP14 suppresses ROS-dependent ferroptosis and alleviates renal ischemia/reperfusion injury. Cell Biochem Biophys. (2023) 81:87–96. doi: 10.1007/s12013-022-01107-y, PMID: 36255562

[87] Tao W Liu F Zhang J Fu S Zhan H Qian K . miR-3587 inhibitor attenuates ferroptosis following renal ischemia-reperfusion through HO-1. Front Mol Biosci. (2022) 8:789927. doi: 10.3389/fmolb.2021.789927, PMID: 35047556 PMC8762253

[88] Ding C Ding X Zheng J Wang B Li Y Xiang H . miR-182-5p and miR-378a-3p regulate ferroptosis in I/R-induced renal injury. Cell Death Dis. (2020) 11:929. doi: 10.1038/s41419-020-03135-z, PMID: 33116120 PMC7595188

[89] Yu Q Gao J Shao X Lu W Chen L Jin L . The effects of alda-1 treatment on renal and intestinal injuries after cardiopulmonary resuscitation in pigs. Front Med (Lausanne). (2022) 9:892472. doi: 10.3389/fmed.2022.892472, PMID: 35646953 PMC9133723

[90] Jiang GP Liao YJ Huang LL Zeng XJ Liao XH . Effects and molecular mechanism of pachymic acid on ferroptosis in renal ischemia reperfusion injury. Mol Med Rep. (2021) 23:63. doi: 10.3892/mmr.2020.11704, PMID: 33215224 PMC7716408

[91] Ma L Liu X Zhang M Zhou L Jiang L Gao L . Paeoniflorin alleviates ischemia/reperfusion induced acute kidney injury by inhibiting Slc7a11-mediated ferroptosis. Int Immunopharmacol. (2023) 116:109754. doi: 10.1016/j.intimp.2023.109754, PMID: 36753983

[92] Yang J Sun X Huang N Li P He J Jiang L . Entacapone alleviates acute kidney injury by inhibiting ferroptosis. FASEB J. (2022) 36:e22399. doi: 10.1096/fj.202200241RR, PMID: 35691001

[93] Yang Y Cai F Zhou N Liu S Wang P Zhang S . Dimethyl fumarate prevents ferroptosis to attenuate acute kidney injury by acting on NRF2. Clin Transl Med. (2021) 11:e382. doi: 10.1002/ctm2.382, PMID: 33931960 PMC8087913

[94] Huang YB Jiang L Liu XQ Wang X Gao L Zeng HX . Melatonin alleviates acute kidney injury by inhibiting NRF2/slc7a11 axis-mediated ferroptosis. Oxid Med Cell Longev. (2022) 2022:4776243. doi: 10.1155/2022/4776243, PMID: 35979396 PMC9377938

[95] Huang J Zhao Y Luo X Luo Y Ji J Li J . Dexmedetomidine inhibits ferroptosis and attenuates sepsis-induced acute kidney injury via activating the Nrf2/SLC7A11/FSP1/CoQ10 pathway. Redox Rep. (2024) 29:2430929. doi: 10.1080/13510002.2024.2430929, PMID: 39581576 PMC11587839

[96] Deng Y Zeng L Liu H Zuo A Zhou J Yang Y . Silibinin attenuates ferroptosis in acute kidney injury by targeting FTH1. Redox Biol. (2024) 77:103360. doi: 10.1016/j.redox.2024.103360, PMID: 39326069 PMC11462067

[97] Wang Z Zhang C . From AKI to CKD: maladaptive repair and the underlying mechanisms. Int J Mol Sci. (2022) 23:10880. doi: 10.3390/ijms231810880, PMID: 36142787 PMC9504835

[98] Chen X Comish PB Tang D Kang R . Characteristics and biomarkers of ferroptosis. Front Cell Dev Biol. (2021) 9:637162. doi: 10.3389/fcell.2021.637162, PMID: 33553189 PMC7859349

[99] Zhao ZB Marschner JA Iwakura T Li C Motrapu M Kuang M . Tubular epithelial cell HMGB1 promotes AKI-CKD transition by sensitizing cycling tubular cells to oxidative stress: A rationale for targeting HMGB1 during AKI recovery. J Am Soc Nephrol. (2023) 34:394–411. doi: 10.1681/ASN.0000000000000024, PMID: 36857499 PMC10103235

[100] Zhou L Xue X Hou Q Dai C . Targeting ferroptosis attenuates interstitial inflammation and kidney fibrosis. Kidney Dis (Basel). (2021) 8:57–71. doi: 10.1159/000517723, PMID: 35224007 PMC8820137

[101] Zhang J Bi J Ren Y Du Z Li T Wang T . Involvement of GPX4 in irisin’s protection against ischemia reperfusion-induced acute kidney injury. J Cell Physiol. (2021) 236:931–45. doi: 10.1002/jcp.29903, PMID: 32583428

[102] Potteti HR Noone PM Tamatam CR Ankireddy A Noel S Rabb H . Nrf2 mediates hypoxia-inducible HIF1α activation in kidney tubular epithelial cells. Am J Physiol Renal Physiol. (2021) 320:F464–74. doi: 10.1152/ajprenal.00501.2020, PMID: 33491566 PMC7988808

[103] Li X Zou Y Xing J Fu YY Wang KY Wan PZ . Pretreatment with Roxadustat (FG-4592) Attenuates Folic Acid-Induced Kidney Injury through Antiferroptosis via Akt/GSK-3β/Nrf2 Pathway. Oxid Med Cell Longev. (2020) 2020:6286984. doi: 10.1155/2020/6286984, PMID: 32051732 PMC6995323

[104] Gong S Zhang A Yao M Xin W Guan X Qin S . REST contributes to AKI-to-CKD transition through inducing ferroptosis in renal tubular epithelial cells. JCI Insight. (2023) 8:e166001. doi: 10.1172/jci.insight.166001, PMID: 37288660 PMC10393228

[105] Linkermann A Skouta R Himmerkus N Mulay SR Dewitz C De Zen F . Synchronized renal tubular cell death involves ferroptosis. Proc Natl Acad Sci U S A. (2014) 111:16836–41. doi: 10.1073/pnas.1415518111, PMID: 25385600 PMC4250130

[106] Wang X Kim CS Adams BC Wilkinson R Hill MM Shah AK . Human proximal tubular epithelial cell-derived small extracellular vesicles mediate synchronized tubular ferroptosis in hypoxic kidney injury. Redox Biol. (2024) 70:103042. doi: 10.1016/j.redox.2024.103042, PMID: 38244399 PMC10831315

[107] Chen F Gao Q Wei A Chen X Shi Y Wang H . Histone deacetylase 3 aberration inhibits Klotho transcription and promotes renal fibrosis. Cell Death Differ. (2021) 28:1001–12. doi: 10.1038/s41418-020-00631-9, PMID: 33024274 PMC7937860

[108] Zhang L Chen F Dong J Wang R Bi G Xu D . HDAC3 aberration-incurred GPX4 suppression drives renal ferroptosis and AKI-CKD progression. Redox Biol. (2023) 68:102939. doi: 10.1016/j.redox.2023.102939, PMID: 37890360 PMC10638610

[109] Zhao L Hao Y Tang S Han X Li R Zhou X . Energy metabolic reprogramming regulates programmed cell death of renal tubular epithelial cells and might serve as a new therapeutic target for acute kidney injury. Front Cell Dev Biol. (2023) 11:1276217. doi: 10.3389/fcell.2023.1276217, PMID: 38054182 PMC10694365

[110] Hwang S Chung KW . Targeting fatty acid metabolism for fibrotic disorders. Arch Pharm Res. (2021) 44:839–56. doi: 10.1007/s12272-021-01352-4, PMID: 34664210

[111] Su L Zhang J Gomez H Kellum JA Peng Z . Mitochondria ROS and mitophagy in acute kidney injury. Autophagy. (2023) 19:401–14. doi: 10.1080/15548627.2022.2084862, PMID: 35678504 PMC9851232

[112] Liu BC Tang TT Lv LL Lan HY . Renal tubule injury: a driving force toward chronic kidney disease. Kidney Int. (2018) 93:568–79. doi: 10.1016/j.kint.2017.09.033, PMID: 29361307

[113] Zhao Z Wu J Xu H Zhou C Han B Zhu H . XJB-5–131 inhibited ferroptosis in tubular epithelial cells after ischemia-reperfusion injury. Cell Death Dis. (2020) 11:629. doi: 10.1038/s41419-020-02871-6, PMID: 32796819 PMC7429848

[114] Martin-Sanchez D Ruiz-Andres O Poveda J Carrasco S Cannata-Ortiz P Sanchez-Niño MD . Ferroptosis, but not necroptosis, is important in nephrotoxic folic acid-induced AKI. J Am Soc Nephrol. (2017) 28:218–29. doi: 10.1681/ASN.2015121376, PMID: 27352622 PMC5198282

[115] Pan M Wang Z Wang Y Jiang X Fan Y Gong F . Celastrol alleviated acute kidney injury by inhibition of ferroptosis through Nrf2/GPX4 pathway. BioMed Pharmacother. (2023) 166:115333. doi: 10.1016/j.biopha.2023.115333.2, PMID: 37598476

[116] Zhang B Chen X Ru F Gan Y Li B Xia W . Liproxstatin-1 attenuates unilateral ureteral obstruction-induced renal fibrosis by inhibiting renal tubular epithelial cells ferroptosis. Cell Death Dis. (2021) 12:843. doi: 10.1038/s41419-021-04137-1, PMID: 34511597 PMC8435531

[117] Hu Q Chen Y Deng X Li Y Ma X Zeng J . Diabetic nephropathy: Focusing on pathological signals, clinical treatment, and dietary regulation. BioMed Pharmacother. (2023) 159:114252. doi: 10.1016/j.biopha.2023.114252, PMID: 36641921

[118] Naaman SC Bakris GL . Diabetic nephropathy: update on pillars of therapy slowing progression. Diabetes Care. (2023) 46:1574–86. doi: 10.2337/dci23-0030, PMID: 37625003 PMC10547606

[119] Li J Li L Zhang Z Chen P Shu H Yang C . Ferroptosis: an important player in the inflammatory response in diabetic nephropathy. Front Immunol. (2023) 14:1294317. doi: 10.3389/fimmu.2023.1294317, PMID: 38111578 PMC10725962

[120] Chen Y Huang G Qin T Zhang Z Wang H Xu Y . Ferroptosis: A new view on the prevention and treatment of diabetic kidney disease with traditional Chinese medicine. BioMed Pharmacother. (2024) 170:115952. doi: 10.1016/j.biopha.2023.115952, PMID: 38056233

[121] Chen J Ou Z Gao T Yang Y Shu A Xu H . Ginkgolide B alleviates oxidative stress and ferroptosis by inhibiting GPX4 ubiquitination to improve diabetic nephropathy. BioMed Pharmacother. (2022) 156:113953. doi: 10.1016/j.biopha.2022.113953, PMID: 36411664

[122] Xu X Xu XD Ma MQ Liang Y Cai YB Zhu ZX . The mechanisms of ferroptosis and its role in atherosclerosis. BioMed Pharmacother. (2024) 171:116112. doi: 10.1016/j.biopha.2023.116112, PMID: 38171246

[123] Zhang S Zhang S Wang H Chen Y . Vitexin ameliorated diabetic nephropathy via suppressing GPX4-mediated ferroptosis. Eur J Pharmacol. (2023) 951:175787. doi: 10.1016/j.ejphar.2023.175787, PMID: 37172926

[124] Kim S Kang SW Joo J Han SH Shin H Nam BY . Characterization of ferroptosis in kidney tubular cell death under diabetic conditions. Cell Death Dis. (2021) 12:160. doi: 10.1038/s41419-021-03452-x, PMID: 33558472 PMC7870666

[125] Huang J Chen G Wang J Liu S Su J . Platycodin D regulates high glucose-induced ferroptosis of HK-2 cells through glutathione peroxidase 4 (GPX4). Bioengineered. (2022) 13:6627–37. doi: 10.1080/21655979.2022.2045834, PMID: 35226829 PMC8973889

[126] Li Y Wang X Zhang Q Tian D Bai Y Feng Y . Dipeptidase 1 promotes ferroptosis in renal tubular epithelial cells in diabetic nephropathy via inhibition of the GSH/GPX4 axis. Int Immunopharmacol. (2024) 133:111955. doi: 10.1016/j.intimp.2024.111955, PMID: 38626544

[127] Wu WY Wang ZX Li TS Ding XQ Liu ZH Yang J . SSBP1 drives high fructose-induced glomerular podocyte ferroptosis via activating DNA-PK/p53 pathway. Redox Biol. (2022) 52:102303. doi: 10.1016/j.redox.2022.102303, PMID: 35390676 PMC8990215

[128] Shen S Ji C Wei K . Cellular senescence and regulated cell death of tubular epithelial cells in diabetic kidney disease. Front Endocrinol (Lausanne). (2022) 13:924299. doi: 10.3389/fendo.2022.924299, PMID: 35837297 PMC9273736

[129] Feng Q Yang Y Ren K Qiao Y Sun Z Pan S . Broadening horizons: the multifaceted functions of ferroptosis in kidney diseases. Int J Biol Sci. (2023) 19:3726–43. doi: 10.7150/ijbs.85674, PMID: 37564215 PMC10411478

[130] Shen J San W Zheng Y Zhang S Cao D Chen Y . Different types of cell death in diabetic endothelial dysfunction. BioMed Pharmacother. (2023) 168:115802. doi: 10.1016/j.biopha.2023.115802, PMID: 37918258

[131] Wu Z Li D Tian D Liu X Wu Z . Aspirin mediates protection from diabetic kidney disease by inducing ferroptosis inhibition. PloS One. (2022) 17:e0279010. doi: 10.1371/journal.pone.0279010, PMID: 36516169 PMC9749971

[132] Geng W Pan L Shen L Sha Y Sun J Yu S . Evaluating renal iron overload in diabetes mellitus by blood oxygen level-dependent magnetic resonance imaging: a longitudinal experimental study. BMC Med Imaging. (2022) 22:200. doi: 10.1186/s12880-022-00939-7, PMID: 36401188 PMC9675154

[133] Zhang Q Hu Y Hu JE Ding Y Shen Y Xu H . Sp1-mediated upregulation of Prdx6 expression prevents podocyte injury in diabetic nephropathy via mitigation of oxidative stress and ferroptosis. Life Sci. (2021) 278:119529. doi: 10.1016/j.lfs.2021.119529, PMID: 33894270

[134] Ma J Li C Liu T Zhang L Wen X Liu X . Identification of markers for diagnosis and treatment of diabetic kidney disease based on the ferroptosis and immune. Oxid Med Cell Longev. (2022) 2022:9957172. doi: 10.1155/2022/9957172, PMID: 36466094 PMC9712001

[135] Feng Q Yu X Qiao Y Pan S Wang R Zheng B . Ferroptosis and acute kidney injury (AKI): molecular mechanisms and therapeutic potentials. Front Pharmacol. (2022) 13:858676. doi: 10.3389/fphar.2022.858676, PMID: 35517803 PMC9061968

[136] Li J Li L Zhang Z Chen P Shu H Yang C . Ferroptosis: an important player in the inflam. Front Immunol. (2023) 14:1294317. doi: 10.3389/fimmu.2023.1294317, PMID: 38111578 PMC10725962

[137] Ke D Zhang Z Liu J Chen P Li J Sun X . Ferroptosis, necroptosis and cuproptosis: Novel forms of regulated cell death in diabetic cardiomyopathy. Front Cardiovasc Med. (2023) 10:1135723. doi: 10.3389/fcvm.2023.1135723, PMID: 36970345 PMC10036800

[138] Miao R Fang X Zhang Y Wei J Zhang Y Tian J . Iron metabolism and ferroptosis in type 2 diabetes mellitus and complications: mechanisms and therapeutic opportunities. Cell Death Dis. (2023) 14:186. doi: 10.1038/s41419-023-05708-0, PMID: 36882414 PMC9992652

[139] Li X Yang Q Liu S Song S Wang C . Mitochondria-associated endoplasmic reticulum membranes promote mitochondrial fission through AKAP1-Drp1 pathway in podocytes under high glucose conditions. Exp Cell Res. (2023) 424:113512. doi: 10.1016/j.yexcr.2023.113512, PMID: 36775185

[140] Yan X Xie Y Liu H Huang M Yang Z An D . Iron accumulation and lipid peroxidation: implication of ferroptosis in diabetic cardiomyopathy. Diabetol Metab Syndr. (2023) 15:161. doi: 10.1186/s13098-023-01135-5, PMID: 37468902 PMC10355091

[141] Zhang Q Deng Q Zhang J Ke J Zhu Y Wen RW . Activation of the nrf2-ARE pathway ameliorates hyperglycemia-mediated mitochondrial dysfunction in podocytes partly through sirt1. Cell Physiol Biochem. (2018) 48:1–15. doi: 10.1159/000491658, PMID: 29996125

[142] He W Chang L Li X Mei Y . Research progress on the mechanism of ferroptosis and its role in diabetic retinopathy. Front Endocrinol (Lausanne). (2023) 14:1155296. doi: 10.3389/fendo.2023.1155296, PMID: 37334304 PMC10268817

[143] Zhang S Li Y Liu X Guo S Jiang L Huang Y . Carnosine alleviates kidney tubular epithelial injury by targeting NRF2 mediated ferroptosis in diabetic nephropathy. Amino Acids. (2023) 55:1141–55. doi: 10.1007/s00726-023-03301-5, PMID: 37450047

[144] Leng J Li X Tian H Liu C Guo Y Zhang S . Neuroprotective effect of diosgenin in a mouse model of diabetic peripheral neuropathy involves the Nrf2/HO-1 pathway. BMC Complement Med Ther. (2020) 20:126. doi: 10.1186/s12906-020-02930-7, PMID: 32336289 PMC7184706

[145] Liu W Liang XC Shi Y . Effects of hirudin on high glucose-induced oxidative stress and inflammatory pathway in rat dorsal root ganglion neurons. Chin J Integr Med. (2020) 26:197–204. doi: 10.1007/s11655-019-2712-8, PMID: 32180149

[146] Liu T Li CY Chen H Liu J Zhong LL Tang MM . tBHQ attenuates podocyte injury in diabetic nephropathy by inhibiting NADPH oxidase-derived ROS generation via the Nrf2/HO-1 signalling pathway. Heliyon. (2022) 8:e10515. doi: 10.1016/j.heliyon.2022.e10515, PMID: 36119860 PMC9479023

[147] Wang C Li C Peng H Ye Z Zhang J Liu X . Activation of the Nrf2-ARE pathway attenuates hyperglycemia-mediated injuries in mouse podocytes. Cell Physiol Biochem. (2014) 34:891–902. doi: 10.1159/000366307, PMID: 25200066

[148] Li S Zheng L Zhang J Liu X Wu Z . Inhibition of ferroptosis by up-regulating Nrf2 delayed the progression of diabetic nephropathy. Free Radic Biol Med. (2021) 162:435–49. doi: 10.1016/j.freeradbiomed.2020.10.323, PMID: 33152439

[149] Jin T Chen C . Umbelliferone delays the progression of diabetic nephropathy by inhibiting ferroptosis through activation of the Nrf-2/HO-1 pathway. Food Chem Toxicol. (2022) 163:112892. doi: 10.1016/j.fct.2022.112892, PMID: 35278496

[150] Li F Hu Z Huang Y Zhan H . Dexmedetomidine ameliorates diabetic cardiomyopathy by inhibiting ferroptosis through the Nrf2/GPX4 pathway. J Cardiothorac Surg. (2023) 18:223. doi: 10.1186/s13019-023-02300-7, PMID: 37430319 PMC10334540

[151] Li B Wang C Lu P Ji Y Wang X Liu C . IDH1 promotes foam cell formation by aggravating macrophage ferroptosis. Biol (Basel). (2022) 11:1392. doi: 10.3390/biology11101392, PMID: 36290297 PMC9598283

[152] Wang H Yu X Liu D Qiao Y Huo J Pan S . VDR activation attenuates renal tubular epithelial cell ferroptosis by regulating nrf2/HO-1 signaling pathway in diabetic nephropathy. Adv Sci (Weinh). (2024) 11:e2305563. doi: 10.1002/advs.202305563, PMID: 38145959 PMC10933633

[153] Lu Q Yang L Xiao JJ Liu Q Ni L Hu JW . Empagliflozin attenuates the renal tubular ferroptosis in diabetic kidney disease through AMPK/NRF2 pathway. Free Radic Biol Med. (2023) 195:89–102. doi: 10.1016/j.freeradbiomed.2022.12.088, PMID: 36581059

[154] Ding L Li ZL Zhou Y Liu NC Liu SS Zhang XJ . Loss of Sirt1 promotes exosome secretion from podocytes by inhibiting lysosomal acidification in diabetic nephropathy. Mol Cell Endocrinol. (2023) 568-569:111913. doi: 10.1016/j.mce.2023.111913, PMID: 36990198

[155] Mao H Wang L Xiong Y Jiang G Liu X . Fucoxanthin attenuates oxidative damage by activating the sirt1/nrf2/HO-1 signaling pathway to protect the kidney from ischemia-reperfusion injury. Oxid Med Cell Longev. (2022) 2022:7444430. doi: 10.1155/2022/7444430, PMID: 35126819 PMC8816562

[156] Ma F Wu J Jiang Z Huang W Jia Y Sun W . P53/NRF2 mediates SIRT1’s protective effect on diabetic nephropathy. Biochim Biophys Acta Mol Cell Res. (2019) 1866:1272–81. doi: 10.1016/j.bbamcr.2019.04.006, PMID: 30959066

[157] Tang X Li X Zhang D Han W . Astragaloside-IV alleviates high glucose-induced ferroptosis in retinal pigment epithelial cells by disrupting the expression of miR-138-5p/Sirt1/Nrf2. Bioengineered. (2022) 13:8240–54. doi: 10.1080/21655979.2022.2049471, PMID: 35302431 PMC9162003

[158] Li Q Liao J Chen W Zhang K Li H Ma F . NAC alleviative ferroptosis in diabetic nephropathy via maintaining mitochondrial redox homeostasis through activating SIRT3-SOD2/Gpx4 pathway. Free Radic Biol Med. (2022) 187:158–70. doi: 10.1016/j.freeradbiomed.2022.05.024, PMID: 35660452

[159] Wang X Shen T Lian J Deng K Qu C Li E . Resveratrol reduces ROS-induced ferroptosis by activating SIRT3 and compensating the GSH/GPX4 pathway. Mol Med. (2023) 29:137. doi: 10.1186/s10020-023-00730-6, PMID: 37858064 PMC10588250

[160] Peng Q Chen X Liang X Ouyang J Wang Q Ren S . Metformin improves polycystic ovary syndrome in mice by inhibiting ovarian ferroptosis. Front Endocrinol (Lausanne). (2023) 14:1070264. doi: 10.3389/fendo.2023.1070264, PMID: 36755918 PMC9900736

[161] Jin H Zhao K Li J Xu Z Liao S Sun S . Matrine alleviates oxidative stress and ferroptosis in severe acute pancreatitis-induced acute lung injury by activating the UCP2/SIRT3/PGC1α pathway. Int Immunopharmacol. (2023) 117:109981. doi: 10.1016/j.intimp.2023.109981, PMID: 37012871

[162] Bányai E Balogh E Fagyas M Arosio P Hendrik Z Király G . Novel functional changes during podocyte differentiation: increase of oxidative resistance and H-ferritin expression. Oxid Med Cell Longev. (2014) 2014:976394. doi: 10.1155/2014/976394, PMID: 25097723 PMC4109136

[163] Tian H Huang Q Cheng J Xiong Y Xia Z . Rev-erbα attenuates diabetic myocardial injury through regulation of ferroptosis. Cell Signal. (2024) 114:111006. doi: 10.1016/j.cellsig.2023.111006, PMID: 38086436

[164] Qian Z Zhang Q Li P Li Y Zhang Y Li R . A disintegrin and metalloproteinase-8 protects against erastin-induced neuronal ferroptosis via activating nrf2/HO-1/FTH1 signaling pathway. Mol Neurobiol. (2024) 61:3490–502. doi: 10.1007/s12035-023-03782-1, PMID: 37995078

[165] Xu Z Sun X Ding B Zi M Ma Y . Resveratrol attenuated high intensity exercise training-induced inflammation and ferroptosis via Nrf2/FTH1/GPX4 pathway in intestine of mice. Turk J Med Sci. (2023) 53:446–54. doi: 10.55730/1300-0144.5604, PMID: 37476875 PMC10387861

[166] Tian X Wang Y Yuan M Zheng W Zuo H Zhang X . Heme oxygenase-1-modified BMMSCs activate AMPK-nrf2-FTH1 to reduce severe steatotic liver ischemia-reperfusion injury. Dig Dis Sci. (2023) 68:4196–211. doi: 10.1007/s10620-023-08102-0, PMID: 37707747 PMC10570260

[167] Wang YH Chang DY Zhao MH Chen M . Glutathione peroxidase 4 is a predictor of diabetic kidney disease progression in type 2 diabetes mellitus. Oxid Med Cell Longev. (2022) 2022:2948248. doi: 10.1155/2022/2948248, PMID: 36275902 PMC9581693

[168] Cai G Liu J Ji Y Dan YD Zhou XH Gao WJ . Ba yang huan wu decoction enhances GPX4 expression to inhibit ferroptosis and alleviate cerebral ischemia-reperfusion injury J. J Chengde Med Coll. (2024) 41:10–4. doi: 10.15921/j.cnki.cyxb.2024.01.001

[169] Tan H Chen J Li Y Li Y Zhong Y Li G . Glabridin, a bioactive component of licorice, ameliorates diabetic nephropathy by regulating ferroptosis and the VEGF/Akt/ERK pathways. Mol Med. (2022) 28:58. doi: 10.1186/s10020-022-00481-w, PMID: 35596156 PMC9123664

[170] Gao X Liu Y Yu X Ding Y . The effect of white flower danin-mediated sirt1-FOXO1 pathway on oxidative stress injury in rats with diabetic nephropathy J. Chin J Gerontol. (2024) 44:170–4. doi: 10.3969/j.issn.1005-9202.2024.01.039

[171] Huang Q Chen H Yin K Shen Y Lin K Guo X . Formononetin attenuates renal tubular injury and mitochondrial damage in diabetic nephropathy partly via regulating sirt1/PGC-1α Pathway. Front Pharmacol. (2022) 13:901234. doi: 10.3389/fphar.2022.901234, PMID: 35645821 PMC9133725

[172] Zhang X Hu W Wu Y Sun K Zhou YL Lu F . Study on the protective effect and mechanism of saikosaponin A on renal structure and function in diabetic rats J. J Guangzhou Univ Chin Med. (2020) 5:1347–53. doi: 10.13359/j.cnki.gzxbtcm.2020.07.026

[173] Wen F Zhang S Sun L Qian M Xu H . Salvianolic acid B inhibits oxidative stress in glomerular mesangial cells alleviating diabetic nephropathy by regulating SIRT3/FOXO1 signaling. Kidney Blood Press Res. (2023) 48:738–51. doi: 10.1159/000534832, PMID: 37935137

[174] Ma B Zhu Z Zhang J . Aucubin alleviates diabetic nephropathy by inhibiting NF-κB activation and inducing SIRT1/SIRT3-FOXO3a signaling pathway in high-fat diet/streptozotocin-induced diabetic mice. J Funct Foods. (2020) 64:103702. doi: 10.1016/j.jff.2019.103702

[175] Ji J Tao P Wang Q Cui M Cao M Xu Y . Emodin attenuates diabetic kidney disease by inhibiting ferroptosis via upregulating Nrf2 expression. Aging (Albany NY). (2023) 15:7673–88. doi: 10.18632/aging.204933, PMID: 37552124 PMC10457067

[176] Chen J Yang L Hao X He Q Hou SJ . To explore the effect and mechanism of genistein on renal injury in rats with diabetic nephropathy based on the Nrf2/HO-1 pathway J. J Modern Integrated Tradit Chin Western Med. (2023) 32:2241–8. doi: 10.3969/j.issn.1008-8849.2023.16.009

[177] Guan X Xie Y Ni W Tang L . Study on the Effect of Nrf2/HO-1/GPX4 on Podocyte Ferroptosis Induced by High Glucose and the intervention mechanism of berberine J. Chin J Pharmacol. (2021) 37:396–403. doi: 10.3969/j.issn.1001-1978.2021.03.018

[178] Lu J Liu A . Crocin regulates the Nrf2/HO-1 pathway to inhibit ferroptosis in human glomerular mesangial cells induced by high glucose J. New Chin Med Clin Pharmacol. (2023) 34:8–15. doi: 10.19378/j.issn.1003-9783.2023.01.002

[179] Feng Q Yang Y Qiao Y Zheng Y Yu X Liu F . Quercetin ameliorates diabetic kidney injury by inhibiting ferroptosis via activating nrf2/HO-1 signaling pathway. Am J Chin Med. (2023) 51:997–1018. doi: 10.1142/S0192415X23500465, PMID: 37046368

[180] Zheng L Guo D . Effects of buyang huanwu decoction on ferroptosis in diabetic nephropathy mice J. Chin J Exp Tradit Med Formulae. (2023) 29:34–41. doi: 10.13422/j.cnki.syfjx.20230839

[181] Wang Z Zou X Zou Y Wang L Wu Y . Exploring the mechanism of Shengqi Dihuang Decoction in inhibiting ferroptosis of human renal tubular epithelial cells induced by high glucose based on the Nrf2/HO-1/GPX4 signaling axis J. Chin J Natural Medicines. (2023) 48:5337–44. doi: 10.19540/j.cnki.cjcmm.20230721.501, PMID: 38114123

[182] Hu X Tang H Dai H Bian L . Study on the protective effect of yuye decoction on renal damage in type 2 diabetic nephropathy rats J. Chin Materia Med. (2020) 43:464–8. doi: 10.13863/j.issn.sn1001-4454.2020.02.039

[183] Zhang P Lu K Xia H He L Ma H Mi L . The effect of modified Huangfeng Decoction on the expression of SIRT1 and PGC-1α in renal tissue of rats with diabetic nephropathy J. Chin J Tradit Chin Med. (2019) 34:589–93. doi: 10.13194/j.issn.1673-842x.2019.12.035

[184] Li Y . Research on the Mechanism of the Traditional Chinese Medicine Compound Yitangkang Regulating SLC7A11/GPX4-Mediated Ferroptosis in the Prevention and Treatment of Diabetic Kidney Injury D. Shenyang: Liaoning University of Traditional Chinese Medicine (2022).

[185] Zhao Z Chen G Bai M Jin Y Tian M Song B . To explore the mechanism of action of Zhenwu Decoction in improving diabetic nephropathy of spleen and kidney Yang deficiency type in mice based on the Nrf2/HO-1/GPX4 signaling pathway J/OL. Chin J Exp Formulas Chin Med. (2023) 30 (01):29–37. doi: 10.13422/j.carol.carroll.nki.syfjx.20230941

[186] Wei H Zhao R Li S Duo H Huo H . Mechanism of action of Tongluo Jiedu Decoction on antioxidant pathways in rats with diabetic nephropathy J. Shaanxi J Tradit Chin Med. (2022) 43:1669–72. doi: 10.3969/j.issn.1000-7369.2022.12.003

[187] Zhao X Li Y Yu J Teng H Wu S Wang Y . Role of mitochondria in pathogenesis and therapy of renal fibrosis. Metabolism. (2024) 155:155913. doi: 10.1016/j.metabol.2024.155913, PMID: 38609039

[188] Liu Y Wang J . Ferroptosis, a rising force against renal fibrosis. Oxid Med Cell Longev. (2022) 2022:7686956. doi: 10.1155/2022/7686956, PMID: 36275899 PMC9581688

[189] Zhang HY Cheng M Zhang L Wang YP . Ferroptosis and renal fibrosis: A new target for the future (Review). Exp Ther Med. (2022) 25:13. doi: 10.3892/etm.2022.11712, PMID: 36561607 PMC9748635

[190] Yang SQ Zhao X Zhang J Liao DY Wang YH Wang YG . Ferroptosis in renal fibrosis: a mini-review. J Drug Targeting. (2024) 32:785–93. doi: 10.1080/1061186X.2024.2353363, PMID: 38721679

[191] Wang J Wang Y Liu Y Cai X Huang X Fu W . Ferroptosis, a new target for treatment of renal injury and fibrosis in a 5/6 nephrectomy-induced CKD rat model. Cell Death Discov. (2022) 8:127. doi: 10.1038/s41420-022-00931-8, PMID: 35318301 PMC8941123

[192] Feng X Wang S Sun Z Dong H Yu H Huang M . Ferroptosis Enhanced Diabetic Renal Tubular Injury via HIF-1α/HO-1 Pathway in db/db Mice. Front Endocrinol (Lausanne). (2021) 12:626390. doi: 10.3389/fendo.2021.626390, PMID: 33679620 PMC7930496

[193] Ide S Kobayashi Y Ide K Strausser SA Abe K Herbek S . Ferroptotic stress promotes the accumulation of pro-inflammatory proximal tubular cells in maladaptive renal repair. Elife. (2021) 10:e68603. doi: 10.7554/eLife.68603, PMID: 34279220 PMC8318592

[194] Zhou Y Zhang J Guan Q Tao X Wang J Li W . The role of ferroptosis in the development of acute and chronic kidney diseases. J Cell Physiol. (2022) 237:4412–27. doi: 10.1002/jcp.30901, PMID: 36260516

[195] Li XT Song JW Zhang ZZ Zhang MW Liang LR Miao R . Sirtuin 7 mitigates renal ferroptosis, fibrosis and injury in hypertensive mice by facilitating the KLF15/Nrf2 signaling. Free Radic Biol Med. (2022) 193(Pt 1):459–73. doi: 10.1016/j.freeradbiomed.2022.10.320, PMID: 36334846

[196] Martínez-Klimova E Aparicio-Trejo OE Tapia E Pedraza-Chaverri J . Unilateral ureteral obstruction as a model to investigate fibrosis-attenuating treatments. Biomolecules. (2019) 9:141. doi: 10.3390/biom9040141, PMID: 30965656 PMC6523883

[197] Kuppe C Ibrahim MM Kranz J Zhang X Ziegler S Perales-Patón J . Decoding myofibroblast origins in human kidney fibrosis. Nature. (2021) 589:281–6. doi: 10.1038/s41586-020-2941-1, PMID: 33176333 PMC7611626

[198] Huang X Song Y Wei L Guo J Xu W Li M . The emerging roles of ferroptosis in organ fibrosis and its potential therapeutic effect. Int Immunopharmacol. (2023) 116:109812. doi: 10.1016/j.intimp.2023.109812, PMID: 36746022

[199] Wang B Yang LN Yang LT Liang Y Guo F Fu P . Fisetin ameliorates fibrotic kidney disease in mice via inhibiting ACSL4-mediated tubular ferroptosis. Acta Pharmacol Sin. (2024) 45:150–65. doi: 10.1038/s41401-023-01156-w, PMID: 37696989 PMC10770410

[200] Dixon SJ Olzmann JA . The cell biology of ferroptosis. Nat Rev Mol Cell Biol. (2024) 25:424–42. doi: 10.1038/s41580-024-00703-5, PMID: 38366038 PMC12187608

[201] Sun L Hao M Sheng M Lü J Weng Y Yu W . The role of AMPK in berberine-alleviating renal fibrosis in mice with renal ischemia-reperfusion: Its relationship with ferroptosis J. Chin J Anesthesiol. (2020) 40:1392–6. doi: 10.3760/cma.j.cn131073.20200313.01129

[202] Li J Yang J Zhu B Fan J Hu Q Wang L . Tectorigenin protects against unilateral ureteral obstruction by inhibiting Smad3-mediated ferroptosis and fibrosis. Phytother Res. (2022) 36:475–87. doi: 10.1002/ptr.7353, PMID: 34964161

[203] Balzer MS Doke T Yang YW Aldridge DL Hu H Mai H . Single-cell analysis highlights differences in druggable pathways underlying adaptive or fibrotic kidney regeneration. Nat Commun. (2022) 13:4018. doi: 10.1038/s41467-022-31772-9, PMID: 35821371 PMC9276703

[204] Liu J Tang D Kang R . Targeting GPX4 in ferroptosis and cancer: chemical strategies and challenges. Trends Pharmacol Sci. (2024) 45:666–70. doi: 10.1016/j.tips.2024.05.006, PMID: 38866667

[205] Miguel V Tituaña J Herrero JI Herrero L Serra D Cuevas P . Renal tubule Cpt1a overexpression protects from kidney fibrosis by restoring mitochondrial homeostasis. J Clin Invest. (2021) 131:e140695. doi: 10.1172/JCI140695, PMID: 33465052 PMC7919728

[206] Friedmann Angeli JP Schneider M Proneth B Tyurina YY Tyurin VA Hammond VJ . Inactivation of the ferroptosis regulator Gpx4 triggers acute renal failure in mice. Nat Cell Biol. (2014) 16:1180–91. doi: 10.1038/ncb3064, PMID: 25402683 PMC4894846

[207] Leonarduzzi G Scavazza A Biasi F Chiarpotto E Camandola S Vogel S . The lipid peroxidation end product 4-hydroxy-2,3-nonenal up-regulates transforming growth factor beta1 expression in the macrophage lineage: a link between oxidative injury and fibrosclerosis. FASEB J. (1997) 11:851–7. doi: 10.1096/fasebj.11.11.9285483, PMID: 9285483

[208] Brigelius-Flohé R Flohé L . Regulatory phenomena in the glutathione peroxidase superfamily. Antioxid Redox Signal. (2020) 33:498–516. doi: 10.1089/ars.2019.7905, PMID: 31822117

[209] Wenk J Schüller J Hinrichs C Syrovets T Azoitei N Podda M . Overexpression of phospholipid-hydroperoxide glutathione peroxidase in human dermal fibroblasts abrogates UVA irradiation-induced expression of interstitial collagenase/matrix metalloproteinase-1 by suppression of phosphatidylcholine hydroperoxide-mediated NFkappaB activation and interleukin-6 release. J Biol Chem. (2004) 279:45634–42. doi: 10.1074/jbc.M408893200, PMID: 15308634

[210] Gong Y Wang N Liu N Dong H . Lipid peroxidation and GPX4 inhibition are common causes for myofibroblast differentiation and ferroptosis. DNA Cell Biol. (2019) 38:725–33. doi: 10.1089/dna.2018.4541, PMID: 31140862

[211] Arbitman L Furie R Vashistha H . B cell-targeted therapies in systemic lupus erythematosus. J Autoimmun. (2022) 132:102873. doi: 10.1016/j.jaut.2022.102873, PMID: 35963808

[212] Siegel CH Sammaritano LR . Systemic lupus erythematosus: A review. JAMA. (2024) 331:1480–91. doi: 10.1001/jama.2024.2315, PMID: 38587826

[213] Hoi A Igel T Mok CC Arnaud L . Systemic lupus erythematosus. Lancet. (2024) 403:2326–38. doi: 10.1016/S0140-6736(24)00398-2, PMID: 38642569

[214] Chen Q Wang J Xiang M Wang Y Zhang Z Liang J . The potential role of ferroptosis in systemic lupus erythematosus. Front Immunol. (2022) 13:855622. doi: 10.3389/fimmu.2022.855622, PMID: 35529869 PMC9068945

[215] Gao X Song Y Wu J Lu S Min X Liu L . Iron-dependent epigenetic modulation promotes pathogenic T cell differentiation in lupus. J Clin Invest. (2022) 132:e152345. doi: 10.1172/JCI152345, PMID: 35499082 PMC9057600

[216] Li P Jiang M Li K Li H Zhou Y Xiao X . Glutathione peroxidase 4-regulated neutrophil ferroptosis induces systemic autoimmunity. Nat Immunol. (2021) 22:1107–17. doi: 10.1038/s41590-021-00993-3, PMID: 34385713 PMC8609402

[217] Seiler A Schneider M Förster H Roth S Wirth EK Culmsee C . Glutathione peroxidase 4 senses and translates oxidative stress into 12/15-lipoxygenase dependent- and AIF-mediated cell death. Cell Metab. (2008) 8:237–48. doi: 10.1016/j.cmet.2008.07.005, PMID: 18762024

[218] Chen Q Xiang M Gao Z Lvu F Sun Z Wang Y . The role of B-cell ferroptosis in the pathogenesis of systemic lupus erythematosus. Clin Immunol. (2023) 256:109778. doi: 10.1016/j.clim.2023.109778, PMID: 37730009

[219] Arazi A Rao DA Berthier CC Davidson A Liu Y Hoover PJ . The immune cell landscape in kidneys of patients with lupus nephritis. Nat Immunol. (2019) 20:902–14. doi: 10.1038/s41590-019-0398-x, PMID: 31209404 PMC6726437

[220] Cheng Q Mou L Su W Chen X Zhang T Xie Y . Ferroptosis of CD163+ tissue-infiltrating macrophages and CD10+ PC+ epithelial cells in lupus nephritis. Front Immunol. (2023) 14:1171318. doi: 10.3389/fimmu.2023.1171318, PMID: 37583695 PMC10423811

[221] Chen J Chen P Song Y Wei J Wu F Sun J . STING upregulation mediates ferroptosis and inflammatory response in lupus nephritis by upregulating TBK1 and activating NF-κB signal pathway. J Biosci. (2024) 49:9. doi: 10.1007/s12038-023-00381-z, PMID: 38186000

[222] Latunde-Dada GO . Ferroptosis: Role of lipid peroxidation, iron and ferritinophagy. Biochim Biophys Acta Gen Subj. (2017) 1861:1893–900. doi: 10.1016/j.bbagen.2017.05.019, PMID: 28552631

[223] Zhou R Yazdi AS Menu P Tschopp J . A role for mitochondria in NLRP3 inflammasome activation. Nature. (2011) 469:221–5. doi: 10.1038/nature09663, PMID: 21124315

[224] Fan Y Ma K Lin Y Ren J Peng H Yuan L . Immune imbalance in Lupus Nephritis: The intersection of T-Cell and ferroptosis. Front Immunol. (2024) 15:1520570. doi: 10.3389/fimmu.2024.1520570, PMID: 39726588 PMC11669548

[225] Morel L ScIndia Y . Functional consequence of Iron dyshomeostasis and ferroptosis in systemic lupus erythematosus and lupus nephritis. Clin Immunol. (2024) 262:110181. doi: 10.1016/j.clim.2024.110181, PMID: 38458303 PMC11672638

[226] Cirillo L Innocenti S Becherucci F . Global epidemiology of kidney cancer. Nephrol Dial Transpl. (2024) 39:920–8. doi: 10.1093/ndt/gfae036, PMID: 38341277

[227] Zhou Q Meng Y Li D Yao L Le J Liu Y . Ferroptosis in cancer: From molecular mechanisms to therapeutic strategies. Signal Transduct Target Ther. (2024) 9:55. doi: 10.1038/s41392-024-01769-5, PMID: 38453898 PMC10920854

[228] Zhang Y Shi J Liu X Feng L Gong Z Koppula P . BAP1 links metabolic regulation of ferroptosis to tumour suppression. Nat Cell Biol. (2018) 20:1181–92. doi: 10.1038/s41556-018-0178-0, PMID: 30202049 PMC6170713

[229] Affar EB Carbone M . BAP1 regulates different mechanisms of cell death. Cell Death Dis. (2018) 9:1151. doi: 10.1038/s41419-018-1206-5, PMID: 30455474 PMC6242853

[230] Zhang L Hobeika CS Khabibullin D Yu D Filippakis H Alchoueiry M . Hypersensitivity to ferroptosis in chromophobe RCC is mediated by a glutathione metabolic dependency and cystine import via solute carrier family 7 member 11. Proc Natl Acad Sci U S A. (2022) 119:e2122840119. doi: 10.1073/pnas.2122840119, PMID: 35867762 PMC9651629

[231] Kerins MJ Milligan J Wohlschlegel JA Ooi A . Fumarate hydratase inactivation in hereditary leiomyomatosis and renal cell cancer is synthetic lethal with ferroptosis induction. Cancer Sci. (2018) 109:2757–66. doi: 10.1111/cas.13701, PMID: 29917289 PMC6125459

[232] Yang WH Ding CC Sun T Rupprecht G Lin CC Hsu D . The hippo pathway effector TAZ regulates ferroptosis in renal cell carcinoma. Cell Rep. (2019) 28:2501–2508.e4. doi: 10.1016/j.celrep.2019.07.107, PMID: 31484063 PMC10440760

[233] Lai Y Zeng T Liang X Wu W Zhong F Wu W . Cell death-related molecules and biomarkers for renal cell carcinoma targeted therapy. Cancer Cell Int. (2019) 19:221. doi: 10.1186/s12935-019-0939-2, PMID: 31462894 PMC6708252

[234] Yu R Zhou Y Shi S Wang X Huang S Ren Y . Icariside II induces ferroptosis in renal cell carcinoma cells by regulating the miR-324-3p/GPX4 axis. Phytomedicine. (2022) 102:154182. doi: 10.1016/j.phymed.2022.154182, PMID: 35636172

[235] Markowitsch SD Schupp P Lauckner J Vakhrusheva O Slade KS Mager R . Artesunate inhibits growth of sunitinib-resistant renal cell carcinoma cells through cell cycle arrest and induction of ferroptosis. Cancers (Basel). (2020) 12:3150. doi: 10.3390/cancers12113150, PMID: 33121039 PMC7692972

[236] Kang R Kroemer G Tang D . The tumor suppressor protein p53 and the ferroptosis network. Free Radic Biol Med. (2019) 133:162–8. doi: 10.1016/j.freeradbiomed.2018.05.074, PMID: 29800655 PMC6251771

[237] Huang Z Gan S Zhuang X Chen Y Lu L Wang Y . Artesunate inhibits the cell growth in colorectal cancer by promoting ROS-dependent cell senescence and autophagy. Cells. (2022) 11:2472. doi: 10.3390/cells11162472, PMID: 36010550 PMC9406496

[238] Ma Z Chen W Liu Y Yu L Mao X Guo X . Artesunate Sensitizes human hepatocellular carcinoma to sorafenib via exacerbating AFAP1L2-SRC-FUNDC1 axis-dependent mitophagy. Autophagy. (2024) 20:541–56. doi: 10.1080/15548627.2023.2261758, PMID: 37733919 PMC10936616

[239] Efferth T . From ancient herb to modern drug: Artemisia annua and artemisinin for cancer therapy. Semin Cancer Biol. (2017) 46:65–83. doi: 10.1016/j.semcancer.2017.02.009, PMID: 28254675

[240] Lee HM Moon A . Amygdalin regulates apoptosis and adhesion in hs578T triple-negative breast cancer cells. Biomol Ther (Seoul). (2016) 24:62–6. doi: 10.4062/biomolther.2015.172, PMID: 26759703 PMC4703354

[241] Du Y Zhao HC Zhu HC Jin Y Wang L . Ferroptosis is involved in the anti-tumor effect of lycorine in renal cell carcinoma cells. Oncol Lett. (2021) 22:781. doi: 10.3892/ol.2021.13042, PMID: 34594422 PMC8456505

[242] Xiao H Xu X Du L Li X Zhao H Wang Z . Lycorine and organ protection: Review of its potential effects and molecular mechanisms. Phytomedicine. (2022) 104:154266. doi: 10.1016/j.phymed.2022.154266, PMID: 35752077

[243] Qi J Meng M Liu J Song X Chen Y Liu Y . Lycorine inhibits pancreatic cancer cell growth and neovascularization by inducing Notch1 degradation and downregulating key vasculogenic genes. Biochem Pharmacol. (2023) 217:115833. doi: 10.1016/j.bcp.2023.115833, PMID: 37769714

[244] Li Z Zhou Q Liu X Li Y Fan X Liu G . Lycorine upregulates the expression of RMB10, promotes apoptosis and inhibits the proliferation and migration of cervical cancer cells. Int J Mol Med. (2022) 50:145. doi: 10.3892/ijmm.2022.5201, PMID: 36367172 PMC9662161

[245] Han S Lin F Qi Y Liu C Zhou L Xia Y . HO-1 contributes to luteolin-triggered ferroptosis in clear cell renal cell carcinoma via increasing the labile iron pool and promoting lipid peroxidation. Oxid Med Cell Longev. (2022) 2022:3846217. doi: 10.1155/2022/3846217, PMID: 35656025 PMC9153929

[246] Chen YH Wu JX Yang SF Hsiao YH . Synergistic combination of luteolin and asiatic acid on cervical cancer *in vitro* and *in vivo*. Cancers (Basel). (2023) 15:548. doi: 10.3390/cancers15020548, PMID: 36672499 PMC9857275

[247] Gupta PB Onder TT Jiang G Tao K Kuperwasser C Weinberg RA . Identification of selective inhibitors of cancer stem cells by high-throughput screening. Cell. (2009) 138:645–59. doi: 10.1016/j.cell.2009.06.034, PMID: 19682730 PMC4892125

[248] Zhou J Liu S Wang Y Dai W Zou H Wang S . Salinomycin effectively eliminates cancer stem-like cells and obviates hepatic metastasis in uveal melanoma. Mol Cancer. (2019) 18:159. doi: 10.1186/s12943-019-1068-1, PMID: 31718679 PMC6852970

[249] Wang Z Zhang H Cheng Q . PDIA4: The basic characteristics, functions and its potential connection with cancer. BioMed Pharmacother. (2020) 122:109688. doi: 10.1016/j.biopha.2019.109688, PMID: 31794946

[250] Zou Y Palte MJ Deik AA Li H Eaton JK Wang W . A GPX4-dependent cancer cell state underlies the clear-cell morphology and confers sensitivity to ferroptosis. Nat Commun. (2019) 10:1617. doi: 10.1038/s41467-019-09277-9, PMID: 30962421 PMC6453886

[251] Juengel E Thomas A Rutz J Makarevic J Tsaur I Nelson K . Amygdalin inhibits the growth of renal cell carcinoma cells *in vitro*. Int J Mol Med. (2016) 37:526–32. doi: 10.3892/ijmm.2015.2439, PMID: 26709398

[252] Rutz J Maxeiner S Juengel E Bernd A Kippenberger S Zöller N . Growth and proliferation of renal cell carcinoma cells is blocked by low curcumin concentrations combined with visible light irradiation. Int J Mol Sci. (2019) 20:1464. doi: 10.3390/ijms20061464, PMID: 30909499 PMC6471746

[253] Escudier B Eisen T Stadler WM Szczylik C Oudard S Siebels M . Sorafenib in advanced clear-cell renal-cell carcinoma. N Engl J Med. (2007) 356:125–34. doi: 10.1056/NEJMoa060655, PMID: 17215530

[254] Dixon SJ Patel DN Welsch M Skouta R Lee ED Hayano M . Pharmacological inhibition of cystine-glutamate exchange induces endoplasmic reticulum stress and ferroptosis. Elife. (2014) 3:e02523. doi: 10.7554/eLife.02523, PMID: 24844246 PMC4054777

[255] Tang Z Li J Shen Q Feng J Liu H Wang W . Contribution of upregulated dipeptidyl peptidase 9 (DPP9) in promoting tumoregenicity, metastasis and the prediction of poor prognosis in non-small cell lung cancer (NSCLC). Int J Cancer. (2017) 140:1620–32. doi: 10.1002/ijc.30571, PMID: 27943262 PMC5324565

[256] Gao R Kalathur RKR Coto-Llerena M Ercan C Buechel D Shuang S . YAP/TAZ and ATF4 drive resistance to Sorafenib in hepatocellular carcinoma by preventing ferroptosis. EMBO Mol Med. (2021) 13:e14351. doi: 10.15252/emmm.202114351, PMID: 34664408 PMC8649869

[257] Park JS Koo KC Chung DY Kim SI Kim J Oh CK . Visceral adiposity as a significant predictor of sunitinib-induced dose-limiting toxicities and survival in patients with metastatic clear cell renal cell carcinoma. Cancers (Basel). (2020) 12:3602. doi: 10.3390/cancers12123602, PMID: 33276522 PMC7761595

[258] Wang Q Gao S Shou Y Jia Y Wei Z Liu Y . AIM2 promotes renal cell carcinoma progression and sunitinib resistance through FOXO3a-ACSL4 axis-regulated ferroptosis. Int J Biol Sci. (2023) 19:1266–83. doi: 10.7150/ijbs.79853, PMID: 36923928 PMC10008700

[259] Gan B . ACSL4, PUFA, and ferroptosis: new arsenal in anti-tumor immunity. Signal Transduct Target Ther. (2022) 7:128. doi: 10.1038/s41392-022-01004-z, PMID: 35459217 PMC9033814

[260] Yangyun W Guowei S Shufen S Jie Y Rui Y Yu R . Everolimus accelerates Erastin and RSL3-induced ferroptosis in renal cell carcinoma. Gene. (2022) 809:145992. doi: 10.1016/j.gene.2021.145992, PMID: 34648917

[261] Jaiswal S Ayyannan SR . Anticancer potential of small-molecule inhibitors of fatty acid amide hydrolase and monoacylglycerol lipase. ChemMedChem. (2021) 16:2172–87. doi: 10.1002/cmdc.202100120, PMID: 33834617

[262] Fiore D Proto MC Pisanti S Picardi P Pagano Zottola AC Butini S . Antitumor effect of pyrrolo-1,5-benzoxazepine-15 and its synergistic effect with Oxaliplatin and 5-FU in colorectal cancer cells. Cancer Biol Ther. (2016) 17:849–58. doi: 10.1080/15384047.2015.1078028, PMID: 26392056 PMC5004676

[263] van Egmond N Straub VM van der Stelt M . Targeting endocannabinoid signaling: FAAH and MAG lipase inhibitors. Annu Rev Pharmacol Toxicol. (2021) 61:441–63. doi: 10.1146/annurev-pharmtox-030220-112741, PMID: 32867595

[264] Hao J Chen Q Feng Y Jiang Q Sun H Deng B . Combination treatment with FAAH inhibitors/URB597 and ferroptosis inducers significantly decreases the growth and metastasis of renal cell carcinoma cells via the PI3K-AKT signaling pathway. Cell Death Dis. (2023) 14:247. doi: 10.1038/s41419-023-05779-z, PMID: 37024452 PMC10079857

[265] Gao Z Zhang Z Gu D Li Y Zhang K Dong X . Hemin mitigates contrast-induced nephropathy by inhibiting ferroptosis via HO-1/Nrf2/GPX4 pathway. Clin Exp Pharmacol Physiol. (2022) 49:858–70. doi: 10.1111/1440-1681.13673, PMID: 35598290

[266] Fang D Wang Y Zhang Z Yang D Gu D He B . Calorie Restriction Protects against Contrast-Induced Nephropathy via SIRT1/GPX4 Activation. Oxid Med Cell Longev. (2021) 2021:2999296. doi: 10.1155/2021/2999296, PMID: 34712381 PMC8548166

[267] Li LX Fan LX Zhou JX Grantham JJ Calvet JP Sage J . Lysine methyltransferase SMYD2 promotes cyst growth in autosomal dominant polycystic kidney disease. J Clin Invest. (2017) 127:2751–64. doi: 10.1172/JCI90921, PMID: 28604386 PMC5490754

[268] Lu Y Sun Y Liu Z Lu Y Zhu X Lan B . Activation of NRF2 ameliorates oxidative stress and cystogenesis in autosomal dominant polycystic kidney disease. Sci Transl Med. (2020) 12:eaba3613. doi: 10.1126/scitranslmed.aba3613, PMID: 32727915

[269] Fan LX Zhou X Sweeney WE Jr Wallace DP Avner ED Grantham JJ . Smac-mimetic-induced epithelial cell death reduces the growth of renal cysts. J Am Soc Nephrol. (2013) 24:2010–22. doi: 10.1681/ASN.2013020176, PMID: 23990677 PMC3839552

[270] Zhang X Li LX Ding H Torres VE Yu C Li X . Ferroptosis promotes cyst growth in autosomal dominant polycystic kidney disease mouse models. J Am Soc Nephrol. (2021) 32:2759–76. doi: 10.1681/ASN.2021040460, PMID: 34716241 PMC8806097

[271] Maser RL Vassmer D Magenheimer BS Calvet JP . Oxidant stress and reduced antioxidant enzyme protection in polycystic kidney disease. J Am Soc Nephrol. (2002) 13:991–9. doi: 10.1681/ASN.V134991, PMID: 11912258

[272] Schreiber R Buchholz B Kraus A Schley G Scholz J Ousingsawat J . Lipid peroxidation drives renal cyst growth *in vitro* through activation of TMEM16A. J Am Soc Nephrol. (2019) 30:228–42. doi: 10.1681/ASN.2018010039, PMID: 30606785 PMC6362630

[273] Wu J Shao X Shen J Lin Q Zhu X Li S . Downregulation of PPARα mediates FABP1 expression, contributing to IgA nephropathy by stimulating ferroptosis in human mesangial cells. Int J Biol Sci. (2022) 18:5438–58. doi: 10.7150/ijbs.74675, PMID: 36147466 PMC9461665

[274] Yuan T Xia Y Li B Yu W Rao T Ye Z . Gut microbiota in patients with kidney stones: a systematic review and meta-analysis. BMC Microbiol. (2023) 23:143. doi: 10.1186/s12866-023-02891-0, PMID: 37208622 PMC10197343

[275] Song Q Liao W Chen X He Z Li D Li B . Oxalate activates autophagy to induce ferroptosis of renal tubular epithelial cells and participates in the formation of kidney stones. Oxid Med Cell Longev. (2021) 2021:6630343. doi: 10.1155/2021/6630343, PMID: 34659638 PMC8514920

[276] Jin Q Liu T Qiao Y Liu D Yang L Mao H . Oxidative stress and inflammation in diabetic nephropathy: role of polyphenols. Front Immunol. (2023) 14:1185317. doi: 10.3389/fimmu.2023.1185317, PMID: 37545494 PMC10401049

[277] Wang WJ Jiang X Gao CC Chen ZW . Salusin-β participates in high glucose-induced HK-2 cell ferroptosis in a Nrf-2-dependent manner. Mol Med Rep. (2021) 24:674. doi: 10.3892/mmr.2021.12313, PMID: 34296310 PMC8335735

[278] Rogacka D Audzeyenka I Rychłowski M Rachubik P Szrejder M Angielski S . Metformin overcomes high glucose-induced insulin resistance of podocytes by pleiotropic effects on SIRT1 and AMPK. Biochim Biophys Acta Mol Basis Dis. (2018) 1864:115–25. doi: 10.1016/j.bbadis.2017.10.014, PMID: 29032153

[279] Motonishi S Nangaku M Wada T Ishimoto Y Ohse T Matsusaka T . Sirtuin1 maintains actin cytoskeleton by deacetylation of cortactin in injured podocytes. J Am Soc Nephrol. (2015) 26:1939–59. doi: 10.1681/ASN.2014030289, PMID: 25424328 PMC4520160

[280] Su H Cantrell AC Chen JX Gu W Zeng H . SIRT3 Deficiency Enhances Ferroptosis and Promotes Cardiac Fibrosis via p53 Acetylation. Cells. (2023) 12:1428. doi: 10.3390/cells12101428, PMID: 37408261 PMC10217433

[281] Rochette L Dogon G Rigal E Zeller M Cottin Y Vergely C . Lipid peroxidation and iron metabolism: two corner stones in the homeostasis control of ferroptosis. Int J Mol Sci. (2022) 24:449. doi: 10.3390/ijms24010449, PMID: 36613888 PMC9820499

[282] Wei M Liu X Tan Z Tian X Li M Wei J . Ferroptosis: a new strategy for Chinese herbal medicine treatment of diabetic nephropathy. Front Endocrinol (Lausanne). (2023) 14:1188003. doi: 10.3389/fendo.2023.1188003, PMID: 37361521 PMC10289168

[283] Chen M Chen Y Zhu W Yan X Xiao J Zhang P . Advances in the pharmacological study of Chinese herbal medicine to alleviate diabetic nephropathy by improving mitochondrial oxidative stress. BioMed Pharmacother. (2023) 165:115088. doi: 10.1016/j.biopha.2023.115088, PMID: 37413900

[284] Belavgeni A Meyer C Stumpf J Hugo C Linkermann A . Ferroptosis and necroptosis in the kidney. Cell Chem Biol. (2020) 27:448–62. doi: 10.1016/j.chembiol.2020.03.016, PMID: 32302582

[285] Gao H Jin Z Bandyopadhyay G Wang G Zhang D Rocha KCE . Aberrant iron distribution via hepatocyte-stellate cell axis drives liver lipogenesis and fibrosis. Cell Metab. (2022) 34:1201–1213.e5. doi: 10.1016/j.cmet.2022.07.006, PMID: 35921818 PMC9365100

[286] Lichtnekert J Anders HJ . Lupus nephritis-related chronic kidney disease. Nat Rev Rheumatol. (2024) 20:699–711. doi: 10.1038/s41584-024-01158-w, PMID: 39317803

[287] Jiang X Stockwell BR Conrad M . Ferroptosis: mechanisms, biology and role in disease. Nat Rev Mol Cell Biol. (2021) 22:266–82. doi: 10.1038/s41580-020-00324-8, PMID: 33495651 PMC8142022

[288] Berndt C Alborzinia H Amen VS Ayton S Barayeu U Bartelt A . Ferroptosis in health and disease. Redox Biol. (2024) 75:103211. doi: 10.1016/j.redox.2024.103211, PMID: 38908072 PMC11253697

