# Correction: Targeting ferroptosis: novel therapeutic approaches and intervention strategies for kidney diseases

**DOI:** 10.3389/fimmu.2025.1765613

**Published:** 2025-12-19

**Authors:** Yanfang Luo, Muyang Long, Xueqin Wu, Liuting Zeng

**Affiliations:** 1 Department of Nephrology, The Central Hospital of Shaoyang, Shaoyang, Hunan, China; 2 Department of Rheumatology and Clinical Immunology, Peking Union Medical College Hospital, Chinese Academy of Medical Sciences and Peking Union Medical College, National Clinical Research Center for Dermatologic and Immunologic Diseases (NCRC-DID), Key Laboratory of Rheumatology and Clinical Immunology, Ministry of Education, Beijing, China

**Keywords:** ferropotosis, AKI, CKD, renal tumor, therapeutic translation, oxidative stress, iron metabolism

Author “Yanfang Luo and Xueqin Wu” was erroneously assigned to affiliation “Department of Nephrology, The Third Xiangya Hospital, Central South University, Changsha, Hunan, China”. This affiliation has now been removed for author “Yanfang Luo and Xueqin Wu”.

The **Conflict of interest** statement has been correct to “No changes to **Conflict of interest:** The authors declare that the research was conducted in the absence of any commercial or financial relationships that could be construed as a potential conflict of interest”.

The original version of this article has been updated.

